# 4-Aminoquinoline-Based Adamantanes as Potential Anticholinesterase Agents in Symptomatic Treatment of Alzheimer’s Disease

**DOI:** 10.3390/pharmaceutics14061305

**Published:** 2022-06-20

**Authors:** Katarina Komatović, Ana Matošević, Nataša Terzić-Jovanović, Suzana Žunec, Sandra Šegan, Mario Zlatović, Nikola Maraković, Anita Bosak, Dejan M. Opsenica

**Affiliations:** 1 Faculty of Chemistry, University of Belgrade, Studentski trg 16, 11000 Belgrade, Serbia; katarinabogojevic@chem.bg.ac.rs (K.K.); mario@chem.bg.ac.rs (M.Z.); 2 Institute for Medical Research and Occupational Health, Ksaverska cesta 2, HR-10 000 Zagreb, Croatia; amatosevic@imi.hr (A.M.); suzana@imi.hr (S.Ž.); nmarakovic@imi.hr (N.M.); 3 Institute of Chemistry, Technology and Metallurgy, National Institute of the Republic of Serbia, University of Belgrade, Njegoševa 12, 11000 Belgrade, Serbia; nterzic@chem.bg.ac.rs (N.T.-J.); sgaica@chem.bg.ac.rs (S.Š.); 4 Centre of Excellence in Environmental Chemistry and Engineering, ICTM, 11000 Belgrade, Serbia

**Keywords:** acetylcholinesterase, butyrylcholinesterase, quinoline, adamantane, selectivity, BBB penetration, drug-likeness, flexible docking, Alzheimer’s disease

## Abstract

Considering that acetylcholinesterase (AChE) inhibition is the most important mode of action expected of a potential drug used for the treatment of symptoms of Alzheimer’s disease (AD), our previous pilot study of 4-aminoquinolines as potential human cholinesterase inhibitors was extended to twenty-two new structurally distinct 4-aminoquinolines bearing an adamantane moiety. Inhibition studies revealed that all of the compounds were very potent inhibitors of AChE and butyrylcholinesterase (BChE), with inhibition constants (*K_i_*) ranging between 0.075 and 25 µM. The tested compounds exhibited a modest selectivity between the two cholinesterases; the most selective for BChE was compound **14**, which displayed a 10 times higher preference, while compound **19** was a 5.8 times more potent inhibitor of AChE. Most of the compounds were estimated to be able to cross the blood–brain barrier (BBB) by passive transport. Evaluation of druglikeness singled out fourteen compounds with possible oral route of administration. The tested compounds displayed modest but generally higher antioxidant activity than the structurally similar AD drug tacrine. Compound **19** showed the highest reducing power, comparable to those of standard antioxidants. Considering their simple structure, high inhibition of AChE and BChE, and ability to cross the BBB, 4-aminoquinoline-based adamantanes show promise as structural scaffolds for further design of novel central nervous system drugs. Among them, two compounds stand out: compound **5** as the most potent inhibitor of both cholinesterases with a *K_i_* constant in low nano molar range and the potential to cross the BBB, and compound **8**, which met all our requirements, including high cholinesterase inhibition, good oral bioavailability, and antioxidative effect. The QSAR model revealed that AChE and BChE inhibition was mainly influenced by the ring and topological descriptors MCD, Nnum, RP, and RSIpw3, which defined the shape, conformational flexibility, and surface properties of the molecules.

## 1. Introduction

Alzheimer’s disease (AD) is a complex neurological disorder, the aetiology of which is associated with clinical hallmarks, such as a decline in neurotransmitter acetylcholine (ACh) levels, amyloid-β (Aβ) peptide deposits, oxidative stress, dyshomeostasis of biometals, and tau protein hyperphosphorylation and accumulation [1,2,3]. Although great efforts have been made over the past several years to develop drugs to treat AD [2,3], they are still limited to alleviating symptoms and improving patients’ quality of life. The U.S. Food and Drug Administration (FDA) has approved five drugs for the treatment of AD: rivastigmine, galantamine, donepezil, memantine, and memantine combined with donepezil [2]. Tacrine, the first centrally acting drug approved for the treatment of AD was discontinued in the United States in 2013. None of these drugs can cure or stop structural and functional neuron damage that causes AD, but they improve the condition of AD patients by increasing ACh levels in the brain [2] as they inhibit the activity of enzymes responsible for ACh hydrolysis or act as *N*-methyl-D-aspartate (NMDA) receptor antagonists [1,2,3]. The effectiveness of these drugs is patient specific, and the effects are only temporary.

As AD is a multifactorial disease that involves several pathophysiological changes, there are other features that could be targeted by drugs [2,3]. One drug that targets Aβ plaques in the brain and reduces them is aducanumab, a monoclonal IgG1 antibody, that was approved by the FDA in an accelerated procedure in June 2021. It is the only drug that has the potential to retard the progression of AD in individuals with mild cognitive impairment or early AD dementia. However, the European Medicines Agency (EMA) has not approved it in the EU, due to the lack of scientific evidence that would confirm clinical benefits of reducing amyloid plaques in the brain [4]. Furthermore, all current FDA-approved drugs target only one AD feature, whereas the multifactorial nature of AD calls for drugs capable of interacting with several targets at the same time and of producing a cumulative treatment effect [3,5,6].

Such drugs could target both enzymes that hydrolyse ACh, i.e., acetylcholinesterase (AChE) and butyrylcholinesterase (BChE). In fact, three currently available AD drugs target either AChE or are nonselective cholinesterase inhibitors [2,7,8]. Recent studies have pointed to BChE as another promising target [8,9], although its physiological role is not completely clear. It seems to act as a backup for AChE and to protect it from neurotoxic agents [7]. Increased BChE activity was reported in certain brain regions affected by AD by up to 120% [8] and in amyloid plaques of AD-affected brains [9]. Selective BChE inhibition (i.e., preference over AChE) has been evidenced to improve learning and lower Alzheimer-amyloid peptide levels in rodents [10]. As AChE and BChE share almost the same backbone structure, with a more than 50% identical amino acid sequence and an active site located in a 20 Å deep gorge [11,12,13], it is possible to target both enzymes. The active site of AChE and BChE is divided into two sub-sites. The first is the catalytic anionic site (CAS), located at the bottom of the gorge and composed of a catalytic triade, oxyanione hole, and a choline-binding site. The second sub-site is the peripheral anionic site (PAS), located at the entrance of the gorge. The peripheral site of AChE consists of Tyr72, Tyr124, Asp74, Tyr341, and Trp286, while Tyr332 and Asp70 are considered as BChE peripheral site [8,14]. Differences in amino acid composition at the CAS and PAS dictate the selectivity of compounds, such as phosphonates, acetates, alcohols, and carbamates [8,15,16,17,18,19], for either cholinesterase. The AChE PAS is involved in the formation of the stable AChE-Aß complex that is more toxic than age-related Aß peptide aggregates [20,21,22,23]. For this reason, inhibitors able to interact with CAS and PAS at the same time (dual site binding inhibitors) could be classified as multi-target drugs worthy of our attention.

Furthermore, as oxidative stress contributes a large part to pathophysiological mechanisms associated with AD [24,25,26], multi-target AD drugs could provide an effective defence against oxidative stress and maintain the redox balance.

In respect to cholinesterase inhibition, quinoline-based compounds have yielded some promising results, as they turn out to be potent inhibitors of both AChE and BChE [27,28,29,30,31,32,33,34,35]. This is particularly true for 4-aminoquinolines, considering their simple structure and high inhibitory potency against AChE [35,36]. Previous studies on aminoquinoline- and adamantyl-based compounds revealed their antioxidant activity through different mechanisms, including chelating ability, electron and hydrogen atom transfer, and reducing power [37,38,39,40,41].

**Figure 1:**
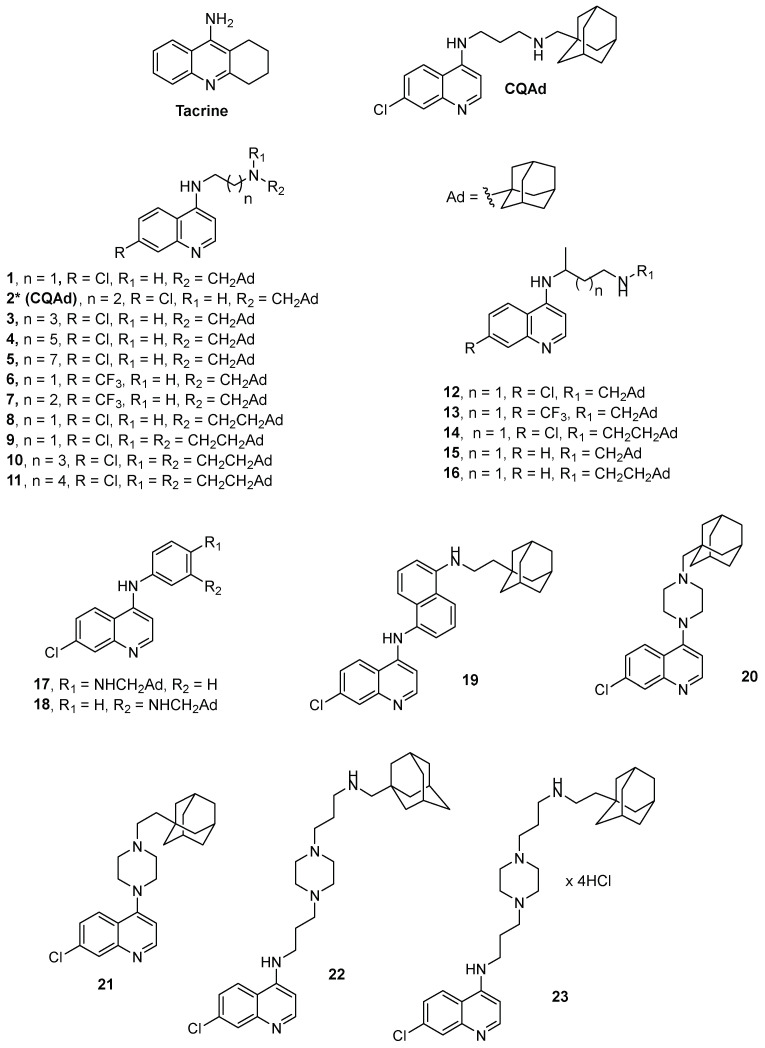
Structure of tacrine, CQAd, and the 4-aminoqiunolines synthesized in this study. * compound **2** was previously reported as CQAd in [36].

Encouraged by our previous findings with 4-aminoquinoline adamantane (**CQAd**, Figure 1) as a promising inhibitor of AChE and BChE [36], twenty-two new 4-aminoquinolines were synthesized with structurally diverse side chains as spacers between the 4-aminoquinoline and adamantyl moieties (Figure 1) to evaluate them as AChE and/or BChE inhibitors and ROS scavengers. Since these compounds are considered CNS active, an in silico analysis of their physicochemical properties was run to estimate their ability to cross the blood–brain barrier (BBB) by passive transport and interpreted the obtained kinetic results through molecular modelling.

## 2. Materials and Methods

### 2.1. Synthesis of Compounds

All chemicals, reagents, and solvents for the preparation of the 4-aminoquinoline derivatives were purchased from commercial sources and were not additionally purified. The compounds (Figure 1) were synthesised according to Schemes S1–S6 in Supplementary Materials File S1. In short, derivatives **1**–**19** and **21** were synthesised following procedures described previously in moderate to good yields [42,43]. The key intermediaries were obtained by heating 4,7-dichloroquinoline (4,7-DCQ) in neat 1,n-diamine in an inert atmosphere (Ar). Otherwise, using 4,7-DCQ and monoprotected diamines in a solvent (phenol or *N*-methylpyperidone (NMP)) at a higher temperature under an inert atmosphere (Ar). In the final step, terminal *N*-adamantyl substituents were introduced via reductive amination using the corresponding adamantyl aldehyde and NaBH_4_. Derivative **20** was obtained by coupling the previously prepared 1-(1-adamantylmethyl)piperazine and 4,7-DCQ at 130 °C in NMP under an inert atmosphere. Compounds **12**, **14–16**, **22** and **23** were described previously [43,44]. All experimental details about the synthesis, spectral characterisation, used instruments and HPLC purity determination are provided in Supplementary Materials File S1.

### 2.2. Inhibition of AChE and BChE

#### 2.2.1. Chemicals

Acetylthiocholine (ATCh) and 5,5′-dithiobis(2-nitrobenzoic acid) (DTNB) were purchased from Sigma-Aldrich, St. Louis, MO, USA. ATCh was dissolved in water and DTNB in 0.1 M sodium phosphate buffer (pH 7.4). The 4-aminoquinilines were dissolved in DMSO and all further dilutions were made in water.

#### 2.2.2. Enzyme Sources

The sources of AChE and BChE were purified human BChE and recombinant human AChE that was kindly provided by Dr Florian Nachon (Département de Toxicologie, Armed Forces Biomedical Research Institute, Brétigny-sur-Orge, France). The concentration of the stock solution of enzymes (BChE: 5.6 µM; AChE: 0.20 µM) was determined as described previously [16]. The enzymes were diluted in sodium phosphate buffer 0.1 M (pH 7.4) containing 0.1% BSA.

#### 2.2.3. Evaluation of Inhibition Constants

Enzyme activities were measured spectrophotometrically using a slightly modified Ellman method, as described earlier [45,46]. Briefly, the AChE and BChE activities were measured at different ATCh concentrations (0.050–0.50 mM) in the absence (*v*_0_) and presence (*v_i_*) of different aminoquinoline concentrations selected to inhibit enzyme activity from 20% to 80% (*i*; final concentrations of 0.001–50 µM, depending on the compound). At least three inhibitor concentrations for each substrate concentration were used in at least three experiments. The apparent inhibition constant (*K_i,app_*) was calculated using the Hunter–Downs Equation and linear regression analysis [47]:

(1) $$K_{i,app}=\frac{v_{i}\cdot i}{v_{0}-v_{i}}=K_{i}+\frac{K_{i}}{K_{S}}\cdot s$$

where the y-intercept determines the enzyme-inhibitor dissociation constant (*K_i_*), while the x-intercept determines the enzyme-substrate dissociation constant, *K_S_*.

The Hunter–Downs Equation was used under the assumption that the substrate, due to relatively low concentrations used in experiments, would bind only to the catalytic site of the enzymes, while the inhibitors would bind to both CAS and/or PAs. This allowed the type of inhibition to be determined from *K_i,app_* vs. substrate concentration ([S]) dependency on Hunter–Downs plot. There where *K_i,app_* is proportional to substrate concentration (i.e., the slope was higher than zero), the compound prevents access to the catalytic site of either AChE or BChE [47]. If the value of the slope is close to zero, the compound does not compete with the substrate for binding to the CAS, which suggests binding to PAS.

The final content of DMSO in the measurements was up to 0.2%. No side interactions of the tested compounds with ATCh or DTNB were detected. The measurements were performed at 25 °C on a Tecan Infinite M200Pro plate reader (Tecan Austria GmbH, Salzburg, Austria).

All kinetic parameters were calculated using the statistical package *GraphPadPrism 8* (Graph Pad Inc., San Diego, CA, USA).

#### 2.2.4. Inhibition Selectivity Evaluation

Inhibition selectivity (IS) was evaluated from the BChE to AChE *K_i_* constant ratio (*K_i_*_(BChE)_/*K_i_*_(AChE)_) where IS > 1 presumes that compound is selective to AChE, while IS < 1 presume BChE selectivity. Compounds with IS ranging from 0.5 to 2 were considered not to selective for either cholinesterase.

### 2.3. pKa Calculation

The p*K*a value of all ionisable sites of the tested compounds was predicted in silico using the Chemicalize 2018 platform [48].

### 2.4. Docking Studies

To rationalize the experimentally determined inhibition potency of the selected compounds and suggest a binding mode, ligands were docked into the enzyme receptors using a flexible docking protocol described elsewhere [49], whereby selected residues outlining the active site gorge of AChE and BChE were allowed to rotate. Ligands to be docked in the enzyme structures were created with ChemBio3D Ultra 13.0 (PerkinElmer, Inc., Waltham, MA, USA) and minimized using the CHARMm force field and Smart Minimizer minimization method of the Minimize Ligands protocol implemented in Biovia Discovery Studio Client v18.1. (Dassault Systèmes, Vélizy-Villacoublay, France). Before the molecular docking was started, the ligands were prepared using the Prepare Ligands protocol with regard to possible different protonation states, isomers and tautomers at pH 7.4.

The enzyme structures were prepared starting from the crystal structures of free AChE (PDB ID: 4EY4) [50] and BChE (PDB ID: 1P0I) [12]. The binding site within AChE and BChE was defined by the sphere surrounding the residues that outline the active site gorge [14,51,52]. The representative pose of each of the docked ligands was chosen based on the highest Consensus score calculated from the scoring functions estimating binding affinity, as implemented in the Biovia Discovery Studio Client v18.1. Score Ligand Poses protocol. A more detailed description of parameters applied in the docking protocol is available in Supplementary Materials File S2.

### 2.5. Antioxidant Activity

The in vitro antioxidant activity of the tested 4-aminoquinolines was evaluated using the ferric reducing antioxidant power (FRAP) assay, in which trolox (water-soluble derivative of vitamin E) and butylated hydroxytoluene (BHT) were used as standard antioxidants, and tacrine as the reference compound. All the chemicals were purchased from Sigma-Aldrich (St. Louis, MO, USA), except for tripyridyltriazine (TPTZ) (Fluka, Buchs, Switzerland) and FeCl_3_ (Kemika, Zagreb, Croatia). The FRAP assay was assessed according to Benzie and Strain [53] with slight procedure adjustments for 96-well microplates [54]. The method is based on the reduction of the ferric-tripyridyltriazine (Fe^3+^-TPTZ) complex to ferrous tripyridyltriazine (Fe^2+^-TPTZ) by the action of electron-donating compounds at a low pH. Briefly, the FRAP reagent was prepared by mixing acetate buffer (300 mM, pH 3.6), a solution of 10 mM TPTZ in 40 mM HCl, and 20 mM FeCl_3_ at 10:1:1 (*v*/*v*/*v*). Then, 240 μL of FRAP reagent and 10 μL of sample solution were added to a 96-well plate and incubated at 37 °C for 60 min. The absorbance was measured at 593 nm using a microplate reader (Infinite M200PRO, Tecan Austria GmbH, Salzburg, Austria) against a blank. The reducing capacity was determined for 10 and 100 µM compound concentrations. The antioxidant activity of tacrine was also tested for comparison due to its structural similarity to the newly synthesized compounds. All measurements were performed in three independent experiments. FRAP values, denominating the reduction of ferric-tripyridyltriazine (Fe^3+^-TPTZ) to ferrous tripyridyltriazine (Fe^2+^-TPTZ) by 4-aminoquinolines were calculated based on a standard curve obtained using Fe_2_SO_4_·7H_2_O.

### 2.6. In Silico Prediction of Druglikeness

The druglikeness of the tested compounds was evaluated under the assumption that the oral route of administration is preferred and that the entry of drugs into cells occurs by passive diffusion. The tested 4-aminoquinolines were evaluated for druglikeness in terms of physicochemical properties rendering them appropriate for oral human use [55,56]: molecular weight (MW), partition coefficient (log*P*), hydrogen bonds donors (HBD), hydrogen bond acceptors (HBA), number of rotatable bonds (RB), and polar surface area (PSA). The topological polar surface area (TPSA) [57], important for the compound to be passively transferred through the interface between blood and an organ (particularly intestinal), was also determined (Table S1 in Supplementary Materials File S2). Generally, compounds with an MW from 180 to 500, *c*log*P* range from 3 to 5, HB ˂ 5, HBA ˂ 1 0, RB ≤ 10, PSA ˂ 120 Å^2^, and those that fail to meet no more than one of the above requirements were considered orally active compounds [55,56]. All physicochemical properties were calculated using the Chemicalize 2018 platform [47] and compared with recommended values [55,56].

### 2.7. In Silico Prediction of Blood–Brain Barrier (BBB) Penetration

Blood–brain barrier (BBB) penetration was predicted using the ADME descriptors protocol for Biovia Discovery Studio Client v18.1. This protocol contains a quantitative linear regression model for the prediction of blood–brain penetration, as well as 95% and 99% confidence ellipses derived from the correlation between polar surface area (PSA-2D) and atom-based Log*P* (AlogP98) parameters derived from over 800 compounds known to enter the CNS after oral administration [57]. BBB penetration is predicted in terms of logBB values as base 10 logarithm of brain concentration/blood concentration. There are four prediction levels within the 95% and 99% confidence ellipsoids with logBB values: 0 (very high penetrants, with logBB ≥ 0.7, where the concentration of a compound in the brain is at least five times higher than in the blood), 1 (high penetrants, with 0 ≤ logBB < 0.7), 2 (medium penetrants with −0.52 < logBB < 0), 3 (low penetrants, with logBB ≤ −0.52, where the brain-blood ratio is less than 0.3:1), and 4 (undefined, outside the confidence ellipsoids).

### 2.8. Chromatographic Determination of Lipophilicity

The lipophilicity of the synthesized compounds was evaluated under reverse-phase thin-layer chromatographic conditions using a vertical developing chamber (CAMAG, Muttenz, Switzerland) on 10 × 10 cm aluminium plates covered with octadecyl-modified silica RP-18W F254s (Art. 5559, Merck, Darmstadt, Germany). The mobile phase contained an organic modifier (methanol, acetone or dioxane), water, and hydrochloric acid. The content of hydrochloric acid was kept constant at 5% *v*/*v*, while the portions of organic modifier and water varied. The dependences of the *R_F_* values on the composition of the mobile phase are given in Tables S1–S3 (Supplementary Materials File S3). The statistical details of retention and corresponding *R_M_*^0^ are summarized in Tables S4–S6 (Supplementary Materials File S3). Mobile phase MeOH/H_2_O/HCl, 70:25:5 (vol%) was used to determine the partition coefficients, log*D*_exp_, of the tested compounds at pH 0.5 (Supplementary Materials File S3 Table S7). Other details about the lipophilicity and log*D*_exp_ determination with the corresponding *Rf*, *R_M_*^0^ and log*D*_exp_ values are provided in the Supplementary Materials File S2 and Supplementary Materials File S3 (Tables S1–S7).

### 2.9. Multivariate Statistical Analysis and Modelling

For multivariate statistical analysis and modelling, the PLS Toolbox software package (v. 5.7 Eigenvectors Inc., Manson, WA USA) for MATLAB (v. 7.8.0 R2009) (MathWorks, Natick, MA, USA) was used. To obtain a data overview, first the input variables were autoscaled [58] and then a principal component analysis (PCA) of both chromatographic data and calculated structural descriptors was run using the singular value decomposition algorithm (SVD) and the 0.95 confidence level fo r Q and T^2^ hotelling limits for outliers. Descriptors with VIP > 1 were chosen as the most contributing descriptors to particular structural characteristic of a compound or biological activity. Descriptors could contribute with a positive or negative sign of the regression coefficient. A positive sign of the regression coefficient indicates that an increase in the value of the corresponding descriptor leads to an increase in particular molecular property, while a negative sign of the regression coefficient indicates that an increase in the value of the corresponding descriptor will lead to a decrease in molecular property. In developing QSAR models, structural descriptors were calculated for molecules in their neutral form and were used as independent variables. Inhibition potency toward human AChE and BChE, expressed as log(*K_i_*/µM), respectively, was used as the dependent variable.

### 2.10. Molecular Descriptors Calculation

Molecules were prepared and their descriptors calculated using the Schrödinger Suite 2021-2 [59]. Molecule structures were built using the Maestro [59] interface, and the Epik [59] module was used to calculate p*K*a values at an experimental pH of 5.0 ± 2 with water as solvent in sequential p*K*a mode, which predicts p*K*a for successive protonation-deprotonation of the molecule. Conformational search was performed with water as the solvent using the MCMM/low-mode conformational search method [60]. Each conformation was minimized in the OPLS 2005 force field using the Polak–Ribiere conjugate gradient method [60] with 2500 iterations or until the 0.05 convergence threshold was obtained, whichever came first. All duplicates were removed and structures within an energy window of 10 kJ mol^−1^ saved. The best conformers were selected and submitted to the calculation of the molecular descriptors using the QikProp module [59] (Supplementary Materials File S3).

## 3. Results and Discussion

Twenty-two compounds are designed to explore the impact of length, conformational flexibility, steric constraints and basicity of the side chain on inhibition towards both AChE and BChE. For that purpose, we used 1,n-diaminoalkanes, normal or branched alkyl-chain, 1,4- or 1,3-diaminobenzene, 1,5-diaminonaphtalene, and different piperazine derivatives as linkers. Additionally, we used methylene or ethylenadamantane as substituents on the terminal amino group to investigate the contribution of steric hindrance in the encirclement of an amine on the cholinesterase inhibition. Finally, the influence of the substituent on the C(7) position of the quinoline ring was also examined.

### 3.1. Inhibition of Cholinesterases

The ability of compounds to inhibit the action of AChE and BChE is expressed with dissociation constants (±standard errors) of the enzyme-ligand complex (*K_i_*) (Table 1).

All of the tested compounds reversibly inhibited AChE activity with *K_i_* constants in the range 0.075–9.0 µM (Table 1). Analysis of the impact of the length of a spacer on the inhibition potency of five unbranched compounds (**1**, **3**, **4**, **6** and **7**) revealed that the elongation of the *n*-alkyl chain from two to six methylene groups did not affect the inhibition potency of those derivatives since they display similar inhibition constants in the 0.7–1.2 µM range, which is not significantly different from the previously tested derivative **2**. However, adding *n*-octylene as a spacer in compound **5** increased its inhibition potency by about 12 times. These findings underline the importance of the shape and length of compounds in terms of the ability of a compound to bind simultaneously to AChE CAS and PAS, as compounds with *n*-octylene as spacer seem to be long and flexible enough to interact with CAS and PAS amino acids.

Replacing chlorine on the C(7) position of the quinoline ring with a more voluminous and stronger electron-withdrawing group, such as the trifluoromethyl group in compounds **6** and **7**, did not change the inhibition potency toward AChE compared to respective reference compounds **1** and **2**.

Replacing the methyleneadamantane group in compound **1** with the ethyleneadamantane group (**8**) also did not change inhibition potency, which suggests that longer distance between terminal nitrogen and the adamantane group does not improve binding with the active site of AChE. The branching of the terminal amino group by adding an extra ethyleneadamantane group, which produced the more voluminous compounds **9** and **10**, lowered the inhibition potency to almost a third of that of compound **8**. In contrast, elongating the spacer in compound **9** by adding three methylene groups (compound **11**) increased the inhibition potency by about 16 times. This increase may be due to the flexibility of *n*-penthyl that allows compound **11** to enter the AChE active site and position itself there more easily.

In the series with branched side chain (**12**–**16**), the effect of replacing methyleneadamantane with ethyleneadamantane on the inhibition potency seemed to have depended on the substituent on C(7) of quinoline, while with Cl-C(7) derivatives, such a change led to a three-fold decrease in the inhibition potency (**12** vs. **14**), in the H-C(7) series it led to a smooth increase (**15** vs. **16**).

Furthermore, replacing diaminoalkanes as spacers with benzene, naphthalene, or piperazine (compounds **17**–**23**) lowered the inhibition potency 28–125 times compared to compound **5**. Generally, introducing a rigid planar aromatic moiety or piperazine with short linkers reduced the flexibility of the conformation and the ability of the compounds to bind to the active site of the enzyme; compound **19** with *meta*-diaminonaphthalene or compound **20** with piperazine were found to be the least active derivatives. The difference in inhibition potency between compounds **17** and **18** clearly demonstrated the importance of dispositioning of the quinoline ring toward the adamantyl moiety.

Compared to the inhibition potency of donepezil, tacrine, and galantamine, AD drugs that reversibly inhibit both cholinesterases, compound **5** stood out as being an only three times less potent AChE inhibitor than donepezil [61]. The inhibition potency of other tested aminoquinolines was in the same range as that of galantamine [61]. Although the tested aminoquinolines could be considered structural analogues of tacrine, their inhibition potency was about 1.9 to 235 times lower. The inhibition constant of tacrine determined in this study for recombinant human AChE confirmed previously reported findings [61,62].

As for BChE, the *K_i_* constants ranged from 0.091 to 25 µM (Table 1). Analysis of the impact of the length of spacer on the inhibition potency revealed that the inhibition potency increased with elongation of the linker.

Derivatives with unbranched ethylene (compound **1**) or propylene (compound **2**) spacers were about 3.2 times less potent inhibitors of BChE than compounds with butylene (**3**) or hexylene (**4**) groups in the spacer. Adding *n*-octylene as a spacer (compound **5**) increased the inhibition by about 30 and 20 times compared to compounds **1** and **2**, respectively, and about 9 times compared to compounds **3** and **4**. This increase may be due to greater conformational freedom resulting from the gradual elongation of the spacer, which consequently allowed more favourable interactions with amino acids in the active site gorge.

Replacing chlorine on the C(7) position on the quinoline ring with a trifluormethyl group in compounds **6** and **7** did not change the inhibition potency compared to reference compounds **1** and **2**.

Replacing the methyleneadamantane group in compound **1** with an ethylenedamantane group (**8**) increased the inhibition by about two times. Branching of the terminal amino group and additional elongation of the spacer in compounds **9**, **10**, and **11** did not change inhibition potency compared to that of compound **3.** The same is true for introducing isobutene as a spacer in compounds **12**, **13**, and **15**. However, adding the methylene group in compound **14** increased BChE inhibition by seven times compared to that of compound **12**.

In contrast, replacing diaminoalkanes as spacers with benzene or naphthalene rings in compounds **17**, **18**, and **19** or piperazine in compound **20** decreased the inhibition by up to 275 times compared to compound **5**, the most potent BChE inhibitor in the present study. It seems that the size and rigidity of the spacer plays a significant role in how quinoline-based adamantyls enter and position themselves in the BChE active site. However, in the piperazine series, increasing the distance between key structural moieties, i.e., adamantane, piperazine, and quinoline, resulted in a gradual increase in the inhibitory potency from *K_i_* = 25 µM (compound **20**) to *K_i_* = 1.8 µM (compound **23**).

In conclusion, the obtained results suggest that the length, volume, rigidity, and number of rotatable bonds in the spacer play a significant role in the inhibition of both cholinesterases, even greater than the addition of basic nitrogen to the side chain (compounds **22** and **23** vs. **5**).

Compared to tacrine, the inhibition potency values of the tested 4-aminoquinolines were about 14–4000 times lower, but were similar to that of donepezil and galantamine [63]. Moreover, compound **5** was 12 and 26 times more potent BChE inhibitor than galantamine and donepezil, respectively. The inhibition constant of tacrine determined in this study for purified human BchE correlated well with the one previously determined for human serum BchE [62,63].

Considering inhibition selectivity, our compounds generally exhibited no pronounced preference for either cholinesterase. Ten compounds were more selective for AchE and three for BchE The most selective was compound **14**, with a 10 times higher preference for BChE, while compound **19** showed a 5.8 times higher preference for AChE than for BChE.

In terms of binding to CAS and/or PAS, most tested compounds that inhibited AChE displayed mixed and competitive type of inhibition without clear connections with their structure. A non-competitive inhibition was displayed by compounds having the trifluoromethyl group (**6** and **7**) on C(7) position and compounds having two methylene groups in the spacer between 4-aminoquinoline and terminal amino group (compound **3**) or combined with two methylene groups between the terminal amino group and adamantly substituent (compound **9**). With BchE, most compounds exhibited a mixed type of inhibition. The exceptions were compound **10**, which showed non-competitive inhibition, and compounds **7**, **9**, **11**, **12**, **19**, **20**, and **22** which showed competitive inhibition.

### 3.2. pKa and Distribution of Protonated Species

The ionization constants of the tested aminoquinolines at physiological pH 7.4 are given in Table 2. Generally, the compounds exist with different ratios of their monoprotonated (protonated terminal amino-group alone) and diprotonated (protonated both the terminal amino-group and the quinoline nitrogen) forms. Nineteen compounds had a protonated terminal amino-group (p*K*_a2,calc_), and their p*K*_a1,calc_ (quinoline nitrogen) was in the 6.90–8.13 range. The dynamic equilibrium between mono- and diprotonated forms of molecules ranged from 76% in favour of the mono protonated form, as in compound **20**, to 26% in compound **15**. Exceptions were compounds **17**, **18**, and **19** in which the non-protonated form dominated.

### 3.3. Docking Results

**Figure 2:**
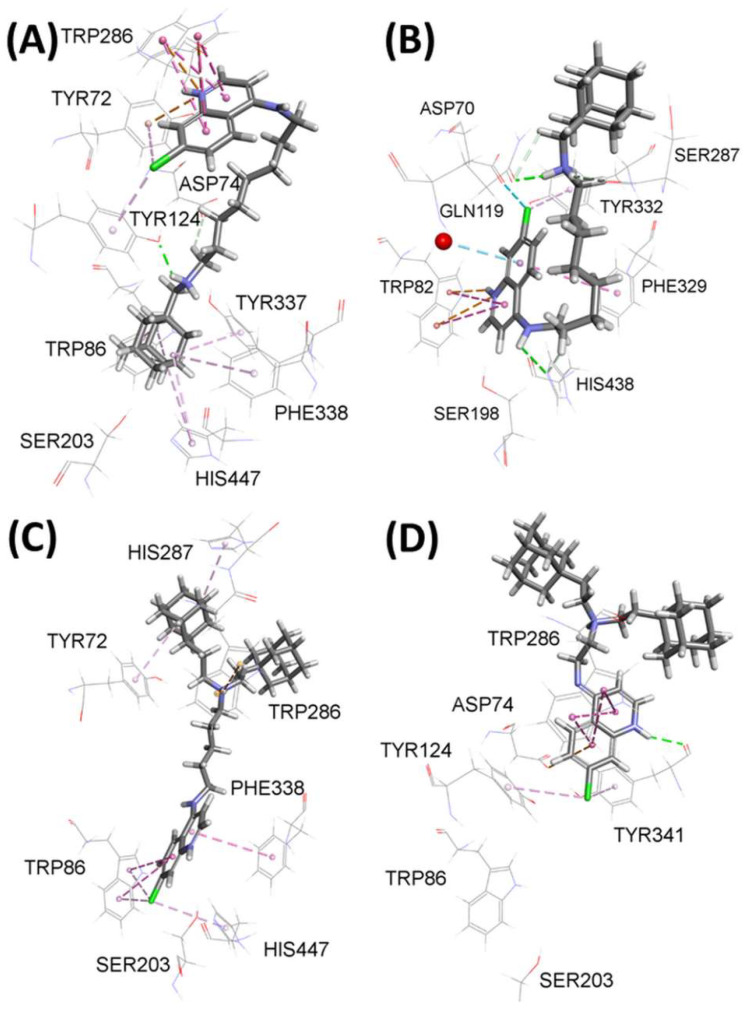
Active site of model complexes between compound **5** and AChE (**A**) and BChE (**B**), compound **11** and AChE (**C**), and compound **9** and AChE (**D**). Dashed lines represent different types of non-binding interactions.

All the tested compounds simultaneously interacted with the AchE amino acids from CAS and/or PAS (Supplementary Materials File S2, pp. S3–S13), that is, they bound to AchE as dual binding site inhibitors. With BchE, they all interacted with amino acids from CAS, but some did not interact with the amino acids from PAS (Supplementary Materials File S2, pp. S13–S21). Figure 2 (panels A and B) shows the docking predicted interactions in AChE or BChE with compound **5**, the most potent inhibitor of both cholinesterases. Compound **5** seems to bind in a bent conformation with an aminoquinoline ring clung to the alkyl spacer. The crucial difference between the two complexes is the orientation of compound **5**. With AChE, its aminoquinoline ring is located in the PAS region (Figure 2, panel A), while with BChE, it is located at the bottom of the active site, near the choline-binding region. This is expected, as BChE has a markedly less defined PAS region, which, in turn, dictates the placement of the aminoquinoline ring in the choline-binding region, where a protonated quinoline ring engages in multiple hydrophobic-π-π T-shaped interactions and additional electrostatic-π-cation interaction with the Trp82 residue (Figure 2, panel B). With AChE, the protonated quinoline ring is located in the PAS region where it is involved in multiple hydrophobic π-π stacked interactions with the distinct PAS residue Trp286 and additional multiple electrostatic-π-cation interactions with another PAS residue Trp72 (Figure 2, panel A). Furthermore, compound **5** adamantane engages in multiple hydrophobic π-alkyl interactions with the surrounding choline binding region residues Trp86, Tyr337, and Phe338 but also with His447 of the catalytic triad (Figure 2, panel A). Besides being involved in an extensive network of aromatic interactions, compound **5** forms hydrogen bonds with neighbouring residues. With AChE, these are Asp74 and Tyr124 (Figure 2, panel A), and with, BChE Gln119, Pro285, Ser287, and His438 (Figure 2, panel B). In addition, its Cl-atom on C(7) is involved in hydrophobic π-alkyl interactions with AChE residues Tyr72 and Tyr123 and BChE residue Tyr332, where an additional halogen type of interaction with residue Asp70 is also present.

As compound **11** exhibited a 16 times higher AChE inhibition potency than compound **9**, the spacer of which is shorter by three methylene units, a better look at their binding modes was undertaken. Figure 2 panel C shows that compound **11** binds at the AChE active site in an elongated conformation spanning the distance between the CAS and PAS subsites. Its aminoquinoline ring is placed in the choline-binding region and is engaged in hydrophobic π-π T-shaped interactions with residues Trp86 and Phe338, and π-alkyl interactions with residues Trp86 and His447 via its aromatic ring and Cl substituent. At the same time, its adamantane ring is located in the PAS region and stabilized through an electrostatic π-cation; π-donor hydrogen bond with Trp286 and π-alkyl interactions with Tyr72 and His287. On the other hand, the three methylene units shorter spacer of compound **9** (Figure 2D) prevents it from protruding deeper into the active site gorge. Instead, its aminoquinoline ring is placed at the edge of the active site gorge where it makes a conventional hydrogen bond with Tyr341, an electrostatic π-anion hydrogen bond with Asp74, a π-alkyl with Tyr124 and Tyr341, and π-π stacked interactions with Trp286.

### 3.4. Predicted Druglikeness and Blood–Brain Barrier (BBB) Penetration

**Figure 3:**
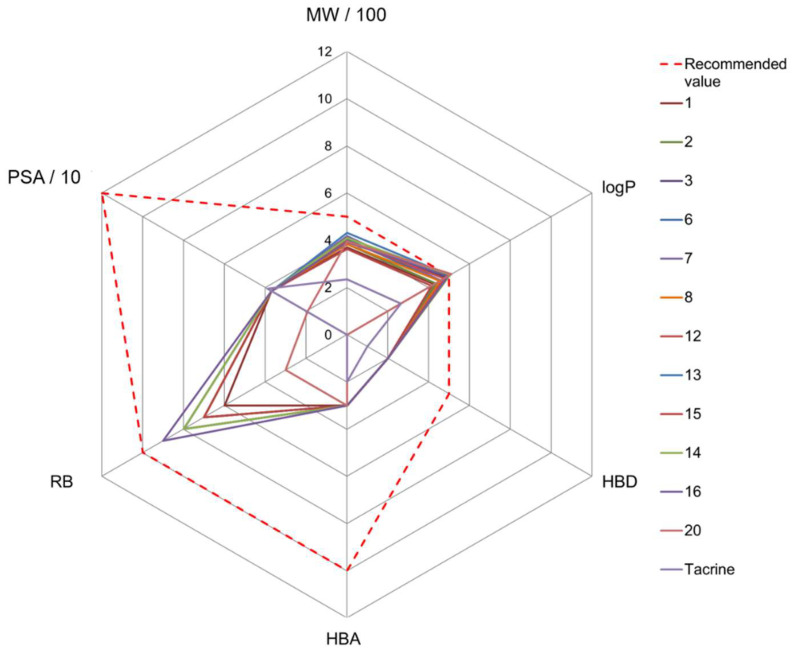
Radar plot of the physicochemical properties (molecular weight, MW; partition coefficient, logP; number of hydrogen bonds donors, HBD, and acceptors HBA; rotatable bonds, RB; polar surface area, PSA) of the tested aminoquinolines. The recommended values for the CNS-active drugs are presented by a dashed red line [55,56]. Presented are only compounds that meet all requirements for oral human use.

All compounds were within the HBD, HBA, and TPSA ranges [64,65,66]. Nine compounds had a higher *c*log*P* value than that recommended, which suggests that they would be retained in the lipid interior of membranes [66] and were therefore excluded from further analysis as CNS-active compounds. Compounds **22** and **23** had higher Mw and RB values than recommended and were also excluded as potentially CNS-active compounds. The twelve compounds remaining met all requirements for oral use in humans, but compounds **13**, **14**, and **20** have limited potential to passively cross lipid membranes due to their high *c*log*P* values bordering the upper recommended limit (Figure 3).

Table 3 shows that 20 of the 23 tested compounds have a very high or high ability to penetrate the BBB. Nine compounds are predicted to be very high BBB penetrants with a logBB range of 0.885–1.487 and eleven to be high BBB penetrants with a logBB in the range of 0.366–0.623. The ADMET_BBB predictions for three compounds were outside the 99% confidence ellipsoids, excluding them from further evaluation as potentially CNS-active compounds.

### 3.5. Lipophilicity and Quantitative Structure-Property Relationship (QSPR)

Increasing the number of methylene groups in the spacer led to an increase in the *R_M_*^0^ and log*D* values in compounds **1**–**5**. Change toward the voluminous and strong electron-withdrawing CF_3_-C(7) group on the quinoline ring led to higher lipophilicity of compounds **6** and **7** in comparison to compounds **1** and **2**. Compounds **9**, **10**, and **11** were the most lipophilic due to the presence of two lipophilic ethyleneadamantane moieties, and their *R_M_*^0^ and log*D*_exp_ values increased in the order **9** < **10** < **11**. Compounds **12**–**16** with a branched spacer had higher lipophilicity than the unbranched analogues. However, H-C(7) analogues **15** and **16** were less lipophilic than their Cl-C(7) analogues **12** and **14**, respectively.

The introduction of aromatic rings in the spacer between the aminoquinoline and adamantane part also increased lipophilicity, while the introduction of piperazine moiety in compounds **20**–**23** had the opposite effect (detailed description of chromatographic determination of lipophilicity and *R_M_*^0^ and log*D* is available in Supplementary Materials Files S2 and S3).

Experimentally determined values of lipophilicity *R_M_*^0^ and log*D*_exp_ were correlated with calculated structural descriptors [66] obtained for the studied compounds in their protonated form (Supplementary Materials File S3). Statistical performances of the derived PLS models and the most contributing descriptors (VIP > 1) are given in Table S3 (Supplementary Materials File S2) in decreasing order with regression coefficients. All of the obtained models are with good predictive abilities and include similar descriptors, which belong to several groups. Ring descriptors are used in the description of ring systems, topological descriptors encode different types of branching and the descriptors from the physicochemical group encode permeation abilities and solubility of the compounds. The best obtained PLS model correlated log*D* values with the degree of ring fusion (RF), ring bridge count (RBC), topological charge indices of order 9 and 3 (TCIO9, TCIO3), valence connectivity index chi-3 (VCIC3), octanol-water partition coefficient (QPlogPo/w), predicted binding for human serum albumin (QPlogKhsa), with a positive sign of regression coefficient, and predicted water/gas partition coefficient (QPlogPw), a normalized number of ring systems (Nnrs), and eccentric (ECC) with a negative sign of the regression coefficients. The most lipophilic compounds, **9**, **10**, and **11**, have the highest values of descriptors, which contribute to higher values of log*D*. Simultaneously, the descriptors that contribute to the decrease in log*D,* are lower for these compounds compared to those of the others.

### 3.6. In Vitro Antioxidative Potential of 4-Aminoquinolines

**Figure 4:**
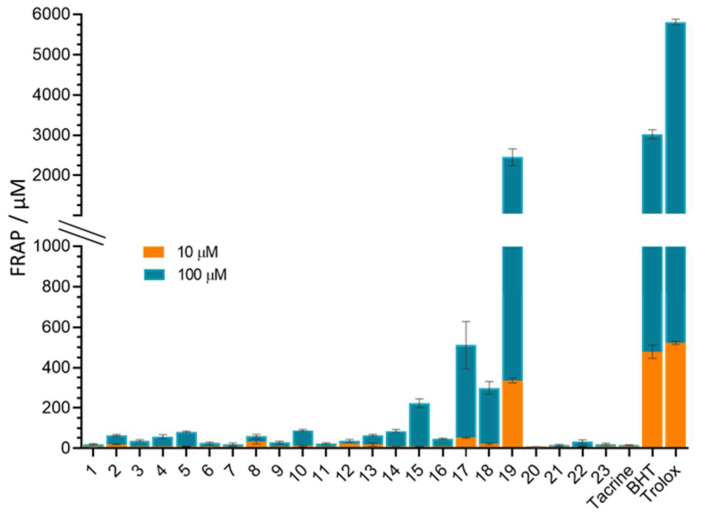
FRAP values (±SE) of the tested aminoquinolines. FRAP values denominate the reduction of ferric-tripyridyltriazine (Fe^3+^ ÷ TPTZ) to ferrous tripyridyltriazine (Fe^2+^-TPTZ) by 4-aminoquinolines and were calculated based on a standard curve obtained using Fe_2_SO_4_·7H_2_O. Blue columns refer to 10 µM and orange to 100 µM compound concentrations.

The reducing capacity of the newly synthetized 4-aminoquinoline-based adamantanes was tested at 10 and 100 µM and expressed as FRAP values listed in Table S2 in Supplementary Materials File S2. Generally, the compounds were very weak reductants compared to standard antioxidants (Figure 4). The exception was compound **19**, which showed very good reducing power comparable to that of BHT and Trolox. However, almost all compounds possessed a certain antioxidant activity compared to tacrine, which showed a negligible reducing power. Compounds **17** and **18** showed a certain antioxidant power, which was on average 10 and 20 times lower than that of Trolox.

It seems that the reduction was improved by replacing alkanes in the spacer with aromatic naphthalene in compound **19** and benzene in compounds **17** and **18**, which corroborates previous reports of very good antioxidant activity accompanied by other neuroprotective activities of tacrine-melatonin, tacrine-8-hydroxyquinoline, and tacrine-caffeic acid hybrids [67,68,69,70,71].

### 3.7. Quantitative Structure–Activity Relationship (QSAR)

Most of the molecular descriptors that contributed to AChE and BChE inhibition are given in Table 4. They include the groups of ring and topological descriptors, which define the shape, conformational flexibility, and surface properties of the molecules. Additionally, BChE inhibition turned out to be highly dependent on potential energy descriptors and less on physicochemical descriptors.

The QSAR model showed that the inhibition potency of the tested compounds toward human AChE is mainly influenced by molecule cyclized degree (MCD), average connectivity index chi-3 (ACIX3), path/walk 3-Randic shape index (RSIpw3) and the second Mohar (2M) descriptor. Among them, MCD has the highest contribution to AChE inhibition, while the contributions of the descriptors ACIX3, RSIpw3 and 2M were lower.

For BChE inhibition, the potency is mostly influenced by potential energy-S-OPLS (PE-S-OPLS), molecule cyclized degree (MCD), path/walk 3-Randic shape index (RSIpw3), topological charge index of order 6 (TCIO6), ring perimeter (RP), normalized number of ring systems (Nnrs), predicted central nervous system activity (CNS), number of ring systems (Nnum) ring complexity index (RCI), ring fusion density (RFD), average valence connectivity index chi-1-5 (AVCIC11-5), π (carbon and attached hydrogen) component of SASA (PISA) and ring fusion degree (RF). Among them, PE-S-OPLS and MCD have the highest contribution to BChE inhibition, while the others have somewhat smaller contributios.

The obtained model confirmed the experimental results that conformationally flexible molecules with more rotatable bonds exhibit higher inhibition potency toward both AChE and BChE. Probably the best illustration is the correlation of MCD descriptors with the *K_i_* values of the compounds. The derivative with highest inhibition potency, compound **5**, had the lowest MCD value (0.625), while conformationally more rigid and least active compound **20**, had the highest MCD value (0.928).

Table 4 also clearly shows no correlation between descriptors related to lipophilicity (QPlogPo/w, logDcalc., log*D*_exp_, and *R_M_*^0^) and inhibition of either AChE or BChE. This suggests that the investigated compounds will be transported through the cell membrane by facilitated transport rather than passive diffusion, especially since they are ionized at the physiological pH.

## 4. General Discussion

We have shown that 4-aminoquinoline-based adamantanes are promising structural scaffolds for the design of novel AD drugs aimed to elevate the symptoms of disease. This is supported by the main favourable features of these compounds: their simple structure, high inhibitory potency toward both cholinesterases, and the ability to cross the BBB as the main requirements for potentially CNS-active compounds. Moreover, the majority of the tested aminoquinolies bind to the AChE active site through simultaneous interactions with amino acids from PAS and CAS as dual binding site inhibitors and could therefore interfere with the formation of an AChE-Aß complex, pointing on those compounds as potential multi-target drugs. The additional feature is their low AChE/BChE selectivity, which indicate their potential to be used in early to late stages of AD, considering that ACh is mainly hydrolysed by AChE in the early stage and by BChE in the late. In this respect, this study has identified fifteen non-selective or BChE-selective compounds as candidates for AD treatment in the middle and late stages of the disease. Considerable antioxidant power of compounds **17**, **18** and **19** to attenuate adverse effects of oxidative stress associated with AD could be considered as an additional target in terms of the design and development of 4-aminoquinolines as multi target drugs This is especially interesting in terms of the ferroptosis, an iron-dependent mechanism of regulated cell death associated with the increase in oxidative stress generated by free radicals formed via the Fenton reaction. Due to its correlation to the etiopathology of AD, ferroptosis is proposed as a promising new target for the treatment of AD [72].

Considering all the beneficial features, this study has singled out compound **5** with the *n*-octyl spacer between the C(4)-amino group on aminoquinoline and methyleneadamantane group on the terminal amino group as the most promising candidate for further evaluation as a potential AD drug. It strongly inhibits both cholinesterases, binds to both PAS and CAS, and has the potential to cross the BBB. Compared to donepezil and tacrine, it is only 2 to 3 times less potent AChE inhibitor, and up to 26 times more potent BChE inhibitor.

## 5. Conclusions

4-aminoquinoline-based adamantanes are promising structural scaffolds for the design of novel anticholinesterase agents in primarily symptomatic treatment of AD, thanks to their simple structure, ability to cross the blood–brain barrier, high inhibition of both cholinesterases, and dual binding to AChE PAS and CAS. Thanks to these features, they have the potential not only to protect against acetylcholine hydrolysis but also against the formation of AChE-Aß complexes, an additional feature that we consider important for our future research of 4-aminoquinolines as potential multi-target-directed ligands in AD treatment. It would also be interesting to see how introducing heteroatoms would improve linker flexibility and, consequently, inhibition potency towards AChE and BChE.

## Figures and Tables

**Table 1:** Acetylcholinesterase (AChE) and butyrylcholinesterase (BChE) inhibition by the tested aminoquinolines expressed as dissociation constants (±standard errors) of the enzyme-ligand complex (*K_i_*).

Compound	*K_i_*/µM		IS
	AChE	BChE	
**1**	1.2 ± 0.1 (c)	2.1 ± 0.2 (m)	1.8
**2** *	0.77 ± 0.09 (m)	3.2 ± 0.4 (m)	4.2
**3**	1.0 ± 0.0 (n)	0.92 ± 0.04 (m)	0.9
**4**	0.67 ± 0.02 (m)	0.76 ± 0.06 (m)	1.1
**5**	0.075 ± 0.06 (m)	0.091 ± 0.007 (m)	1.2
**6**	1.2 ± 0.1 (n)	3.5 ± 0.3 (m)	2.9
**7**	1.1 ± 0.0 (n)	2.6 ± 0.2 (c)	2.4
**8**	1.6 ± 0.1 (n)	0.92 ±0.07 (m)	0.6
**9**	5.2 ± 0.2 (n)	1.5 ± 0.2 (c)	0.3
**10**	2.4 ± 0.2 (n)	1.0 ± 0.1 (n)	0.4
**11**	0.33 ± 0.01 (m)	0.82 ± 0.04 (c)	2.5
**12**	0.56 ± 0.02 (m)	1.2 ± 0.1 (c)	2.1
**13**	0.91 ± 0.05 (m)	1.8 ± 0.3 (c)	1.5
**14**	1.9 ± 0.1 (m)	0.15 ± 0.01 (m)	0.1
**15**	0.74 ± 0.03 (m) C	0.65 ± 0.04 (m) M	0.9
**16**	0.52 ± 0.02 (c)	0.38 ± 0.03 (m)	0.7
**17**	3.3 ± 0.4 (m) M	2.5 ± 0.5 (m)	0.8
**18**	9.0 ± 0.8 (m)	5.5 ± 0.6 (m)	0.6
**19**	3.8± 0.3 (c)	22 ± 2 (c)	5.8
**20**	9.4± 0.4 (c)	25± 1 (c)	2.7
**21**	2.1± 0.4 (m)	5.6 ± 0.6 (m)	2.7
**22**	0.69 ± 0.02 (c)	3.3 ± 0.2 (c)	4.8
**23**	0.44 ± 0.09 (c)	1.8 ± 0.2 (m)	4.1
Tacrine	0.040 ± 0.006 (m)	0.0063 ± 0.0010 (m)	

* [38]; IS = *K_i_*_(BChE)_/*K_i_*_((AChE)_; letters c, n, and m stand for competitive, non-competitive, and mixed type of inhibition, respectively.

**Table 2:** In silico p*K*a_,calc_ values of the tested aminoquinolines.

Compound	p*K*_a1 calc_ (Quinoline)	p*K*_a2 calc_ (Terminal Amino-Group)	p*K*_a3 calc_/p*K*_a4 calc_ (Side Chain)
**1**	7.25	9.92	-
**2** *	7.31	10.55	-
**3**	7.31	10.85	-
**4**	7.31	10.86	-
**5**	7.31	10.86	-
**6**	7.48	9.93	-
**7**	7.53	10.55	-
**8**	7.25	9.33	-
**9**	7.23	10.05	-
**10**	7.31	10.98	-
**11**	7.31	10.98	-
**12**	7.29	10.58	-
**13**	7.51	10.58	-
**14**	7.28	10.50	-
**15**	8.13	10.58	-
**16**	8.13	10.78	-
**17**	6.22	6.85	-
**18**	6.49	5.35	-
**19**	6.45	4.71	-
**20**	6.90	9.13	-
**21**	6.91	8.92	-
**22**	7.12	10.50	8.07/1.24
**23**	7.12	10.42	8.06/1.23
Tacrine	8.95	-	-

* Compound **2** was previously reported as CQAd in [36].

**Table 3:** In silico-determined ability of the tested aminoquinolines to pass the blood–brain barrier by passive transport.

	ADMET_BBB Level				
	0	1	2	3	4
Compounds	**4, 5, 9, 14, 16–18, 20, 21**	**1–3, 6–8, 12, 13, 15, 22, 23, Tacrine**	-	-	**10, 11, 19**

**Table 4:** QSAR models for the correlation of molecule descriptors and inhibition potency of compounds towards AChE and BChE (*K_i_*) *.

Dependent Variable	Statistical Performance of the Model	Structural Descriptors Included in the QSAR Model **
*log (K_i_/µM, AChE)*	RMSEC = 0.222, RMSECV = 0.367, RMSEP = 0.408 R^2^_cal_ = 0.708, R^2^_CV_ = 0.333, R^2^_pred_ = 0.603 PLS1: 62.13% and 55.44% PLS2: 5.16% and 15.39%	MCD (+),2M (−), ACIX3 (+), ACIX5 (+), RSIpw3 (+)
*log (K_i_/µM, BChE)*	RMSEC = 0.132, RMSECV = 0.233, RMSEP = 0.333 R^2^_cal_ = 0.943, R^2^_CV_ = 0.825, R^2^_pred_ = 0.777 PLS1: 40.06% and 79.81% PLS2: 17.79% and 8.76% PLS1: 11.44% and 3.62% PLS2: 12.57% and 2.13%	PE-S-OPLS (+),MCD (+), RSIpw3 (+), TCIO6 (+) RCI (−), RFD (−), RP (+), AVCIC5 (−), RF (−), AVCIC4 (−), Nnrs (+), AVCIC3 (−), AVCIC2 (−), CNS (+), PISA (+), AVCIC1 (−), Nnum (+), QPPMDCK (+)

* Details of the obtained QSAR models and corresponding graphics that illustrate the contribution of structural descriptors to AChE and BChE inhibition are provided in Graphics S5 and S6 (Supplementary Materials File S3). ** For abbreviations and a complete list of molecular descriptors see Supplementary Materials File S3.

## Data Availability

Data sharing is not applicable to this article.

## Supplementary Materials

The following supporting information can be downloaded at: https://www.mdpi.com/article/10.3390/pharmaceutics14061305/s1, References [73,74,75,76,77,78,79] are cited in the supplementary materials. Supplementary Materials File S1: Synthesis, spectral data, NMR spectra and HPLC purity chromatograms. Supplementary Materials File S2: molecular modelling, ligand-enzyme interaction diagrams, predictions of drug-likeness and BBB penetration, multivariate statistical analysis PCA and PLS modelling, Supplementary Materials File S3: List of molecular descriptors, compounds molecular descriptors data matrix, details of PLS models: RM0 MeOH, RM0 Diox, RM0 AC, log PYO, logD.
