# Supplementary material for: 4-Aminoquinoline-Based Adamantanes as Potential Anticholinesterase Agents in Symptomatic Treatment of Alzheimer’s Disease
Source: Pharmaceutics. 2022 Jun 20;14(6):1305. doi: 10.3390/pharmaceutics14061305 (PMC9229919; doi:10.3390/pharmaceutics14061305)
Supplement: Supplementary file 1 [file pharmaceutics-14-01305-s001.zip › Supplementary Materials File S1_final_Proof 2_Opsenica.pdf]

Supplementary Material 1

4-aminoquinolines-based adamantanes as potential anticholinesterase agents in symptomatic treatment of Alzheimer's disease

Katarina Komatović[1], Ana Matošević[2], Suzana Žunec[2], Nataša Terzić-Jovanović[3], Sandra Šegan[3], Mario Zlatović,[1] Nikola Maraković[2], Anita Bosak[2,*], Dejan Opsenica[3,4,*]

[1] University of Belgrade - Faculty of Chemistry, Studentski trg 12 -16, 11158 Belgrade, Serbia,

[2] Institute for Medical Research and Occupational Health, Ksaverska cesta 2, Zagreb, Croatia,

[3] University of Belgrade – Institute of Chemistry, Technology and Metallurgy (IHTM), Njegoševa 12, 11000 Belgrade, Serbia,

[4] Centre of Excellence in Environmental Chemistry and Engineering, ICTM, 11000 Belgrade, Serbia

* Correspondence: abosak@imi.hr (A.B.); dopsen@chem.bg.ac.rs (D.O.)

Content:

Synthesis and spectral data S2

References S19

Synthesis and spectral data

All of the chemicals, reagents and solvents for the preparation of 4-aminoquinoline derivatives were purchased from commercial sources.

IR spectra were recorded on a Perkin-Elmer spectrophotometer FT-IR 1725X (Perkin-Elmer, Waltham, MA 02451, USA). Positions of absorbance band are expressed in cm^{-1} and intensity is labelled as w (weak), m (medium) or s (strong). ^{1}H and ^{13}C NMR spectra were recorded on a Varian Gemini-200 spectrometer (Agylent Technology, Santa Clara, CA 95051, USA) (at 200 and 50 MHz, for a ^{1}H and ^{13}C NMR spectra, respectively), on a Varian /Agilent spectrometer (Agylent Technology, Santa Clara, CA 95051, USA) (at 400 and 100 MHz, for a ^{1}H and ^{13}C NMR spectra, respectively) and on a Bruker Ultrashield Advance III spectrometer (Bruker Scientific Instruments, Billerica, MA 01821, USA) (at 500 and 125 MHz, for a ^{1}H and ^{13}C NMR spectra, respectively) employing indicated solvents using TMS as the internal standard. Chemical shifts are expressed in ppm (δ) values and coupling constants (J) in Hz. ESI-MS spectra were recorded on Agilent Technologies 6210 Time-Of-Flight LC-MS instrument (Agylent, Santa Clara, CA 95051, USA) in positive ion mode with CH_3CN/H_2O-0.2 % HCOOH as eluent. Samples were dissolved in CH_3CN or MeOH (HPLC grade purity). TOF-MS spectra were recorded on 4800 Plus MALDI TOF/TOF Analyzer (Applied Biosystems, US instrument, Mundelein, USA) with positive ion mode (fixed laser intensity 4280) using CH_3CN/H_2O gradient with 0.2% HCOOH as the carrying solvent solution. Samples were dissolved in MeOH (HPLC grade purity) mixed with CHCA (α-Cyano-4-hydroxycinnamic acid) MALDI matrix (5 mg/mL in 50% acetonitrile (v/v)). Internal calibrants: Azithromycin and Azithromycin fragments (m/z 156-749). Dry-flash chromatography was performed on silica gel 40-63 μ m and thin-layer chromatography (TLC) on precoated Merck silica gel 60 F254 and RP-18 F254 plates using UV light, cerium-ammonium molibdate (CAM) and Co(II)-thiocyanate for chromatogram visualization. Compounds were analysed for purity using an Agilent 1260 Infinity HPLC system equipped with Quat Pump (G1311B), Injector (G1329B) 1260 ALS, TCC 1260 (G1316A) and Detector 1260 DAD VL+ (G1315C) (Agylent, Santa Clara, CA 95051 United States). Compounds were dissolved in MeOH, final concentrations were ~ 0.5mg/mL. Flow rate was 0.5 ml/min. Compounds were eluted using gradient protocol: 0 – 1 min 95% solvent A, 1 - 6 min 95% solvent A→ 5% solvent A, 6 - 11 min 5% solvent A, 11 – 14 min 5% solvent A→ 95% solvent A, 14 – 15 min 95% solvent A. HPLC analysis was performed by four different methods:

- *Method I* - Zorbax Eclipse Plus C18 4.6 x 100mm, 1.8μ, was used as the stationary phase. The eluent was made from the following solvents: solvent A=0.2% HCOOH in H_2O and solvent B=CH_3CN. The analysis was performed at the UV max of the compounds to maximize selectivity;

- *Method II* - Zorbax Eclipse Plus C18 4.6 x 100mm, 1.8µ was used as the stationary phase. The eluent was made from the following solvents: solvent A=0.2% HCOOH in H_2O and solvent B=MeOH. The analysis was performed at the UV max of the compounds to maximize selectivity;

- *Method III* - Zorbax Eclipse Plus C18 4.6 x 150mm, 1.8µ, S.N. USWKY01594 was used as the stationary phase. The eluent was made from the following solvents: solvent A=0.2% HCOOH in H_2O and solvent B=CH_3CN;

- *Method V* - Zorbax Eclipse Plus C18 4.6 x 150mm, 1.8µ, S.N. USWKY01594 was used as stationary phase. The eluent was made of solvent A=0.2% HCOOH in H_2O and solvent B=MeOH.

Synthesis of the derivatives **1**, **2**, **3**, **8**, **9**, **12**, **14**, **15**, **16**, **22** and **23** (Schemes S1–S3) were reported previously [73,1, 2]. Derivatives **1**, **12**, **14**, **15**, **16**, **22** and **23** were used from a laboratory set of compounds, while others were synthesized during this research.

Diaminoquinolines **26** – **33** (Scheme S1) were synthesized according to the procedure described in the literature, starting from 4,7-dichloroquinoline (**24**) or 4-chloro-7-trifluoroquinoline (**25**), and their spectra were identical to those published [74,75].

Scheme S1

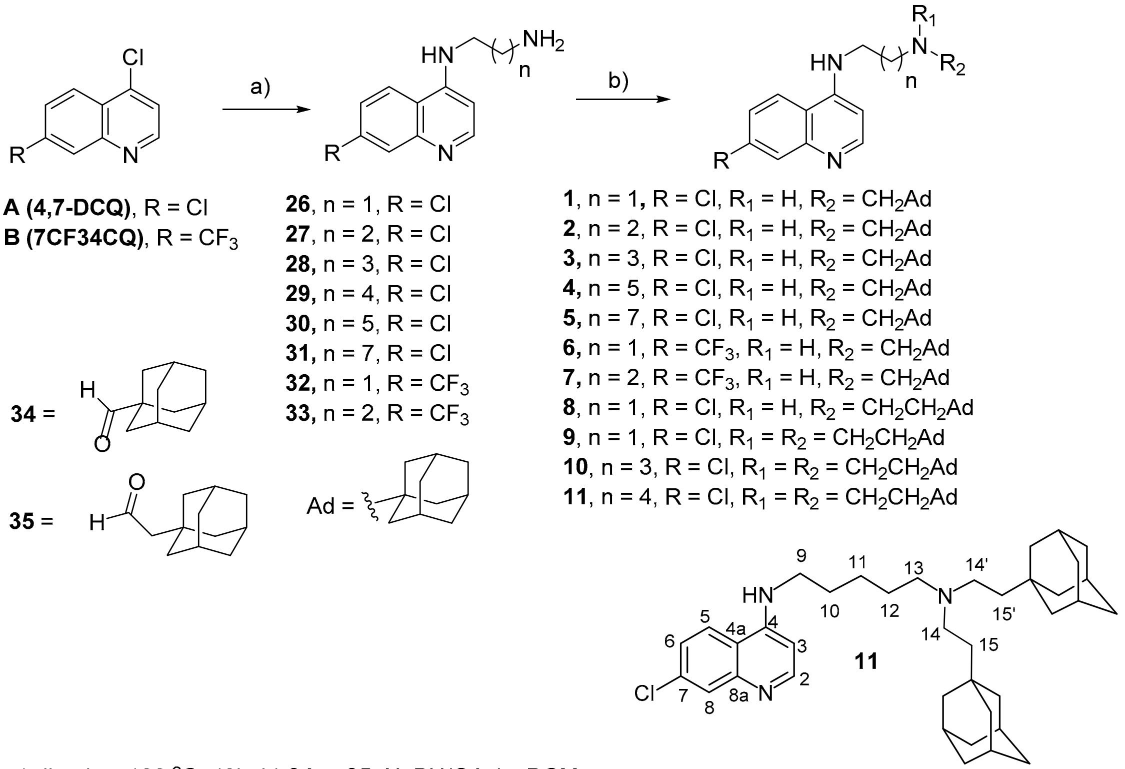

a) diamine, 130 °C, 48h; b) **34** or **35**, $NaBH(OAc)_3$, DCM, r.t.

*General procedure for the synthesis of derivatives **3 – 11***:

N^1-(1-adamantylmethyl)-N^2-(7-chloro-4-quinolinyl)-1,4-buthanediamine (3).

Into the solution of **28** (120 mg, 0.48 mmol) and the aldehyde **34** (78.9 mg, 0.48 mg) in CH_2Cl_2 (10 mL), using $NaHB(OAc)_3$ (203.7 mg, 0.96 mmol) was added. After stirring the mixture at room temperature for 24 h, the aqueous NaOH (1 M) was introduced. The organic layer was separated, and the aqueous layer was washed with CH_2Cl_2. The combined organic layers were washed once with brine and dried over anh. Na_2SO_4, the solvent was evaporated, and the residue was purified by dry-flash chromatography (silica-gel, gradient CH_2Cl_2/MeOH/NH_3(aq). Yield 95.7 mg (50%) pale yellow oil. IR (ATR): 3235m, 3140w, 3099w, 3058w, 3004w, 2901s, 2844s, 2807m, 2778w, 2741w, 2656w, 1608m, 1572s, 1540m, 1472w, 1451m, 1434m, 1369m, 1327m, 1344w, 1285w, 1251w, 1215w, 1196w, 1168w, 1100w, 1056w, 1017w, 905w, 875w, 851w, 826w, 804w, 765w. ^{1}H NMR (500 MHz, mixture of CD_3OD and $CDCl_3$, δ): 8.32 (d, J = 5.6, 1H, H-C(2)), 8.07 (d, J = 9.0, 1H, H-C(5)), 7.76 (d, J = 2.1, 1H, H-C(8)), 7.36 (dd, J_1 = 9.0, J_2 = 2.2, 1H, H-C(6)), 6.47 (d, J = 5.6, 1H, H-C(3)), 3.41 – 3.33 (m, 2H, H-C(9)), 2.66 – 2.56 (m, 2H, H-C(12)), 2.22 (s, 2H, H-C(13)), 1.98 – 1.88 (m, 3H, 3x*CH*(Ad)), 1.81 – 1.56 (m, 10H, H-C(10), H-C(11), 3xCH_2(Ad)), 1.51 (d, J = 2.9, 6H, 3xCH_2(Ad)). ^{13}C NMR (126 MHz, mixture of CD_3OD and $CDCl_3$, δ): 151.21(C4), 150.91(C2), 148.27(C8a), 134.90(C7), 126.27(C8), 124.56(C5), 122.81(C6), 117.38(C4a), 98.21(C3), 62.25(C13), 50.05(C12), 42.38(C9), 40.44(3xCH_2(Ad)), 36.73(3xCH_2(Ad)), 32.78(C(Ad)), 28.36(3xCH(Ad)), 26.19(C11), 25.61(C10). (+)TOF-HRMS (*m/z*): calc. for $[C_{24}H_{32}ClN_3 + H]^+$ 398.2363, found 398.2350. HPLC purity: Method I: RT 8.657, area 97.49 %; Method II: RT 11.190, area 97.94 %.

N^1-(1-adamantylmethyl)-N^2-(7-chloro-4-quinolinyl)-1,6-hexanediamine (4).

Compound **4** was obtained from amine **30** (95.4 mg, 0.34 mmol) and aldehyde **34** (56.4 mg, 0.34 mmol), using $NaHB(OAc)_3$ (145.6 mg, 0.69mmol). Yield 62.7 mg (43 %), as pale yellow powder. IR (ATR): 3238m, 3060m, 2900s, 2847s, 1609m, 1575s, 1540m, 1452m, 1431m, 1368m, 1333m, 1281w, 1249w, 1207w, 1136w, 1098w, 180w, 899w, 874w, 850w, 808w, 768w. ^{1}H NMR (500 MHz, CD_3OD, δ): 8.41 (d, J = 5.6, 1H, H-C(2)), 8.16 (d, J = 9.0, 1H, H-C(5)), 7.84 (d, J = 2.3, 1H, H-C(8)), 7.45 (dd, J_1 = 9.0, J_2 = 2.2, 1H, H-C(6)), 6.56 (d, J = 5.6, 1H, H-C(3)), 3.42 (t, J = 7.2, 2H, H-C(9)), 2.60 (dd, J_1 = 8.5, J_2 = 6.7, 2H, H-C(14)), 2.27 (s, 2H, H-(C15)), 2.08 – 1.95 (m, 3H, 3xCH(Ad)), 1.90 – 1.69 (m, 8H, CH_2 and 3xCH_2(Ad)), 1.67 – 1.51 (m, 10H, 2xCH_2 and 3xCH_2(Ad)), 1.47 (q, J = 7.9, 2H, CH_2). ^{13}C NMR (125 MHz, CD_3OD, δ): 151.56(C4), 151.23(C2), 148.56(C8a), 135.08(C7), 126.45(C8), 124.71(C5), 123.12(C6), 117.61(C4a), 98.40(C3), 62.67(C15), 50.73(C14), 42.73(C9), 40.69(3xCH_2(Ad)), 36.99(3xCH_2(Ad)), 33.02(C(Ad)), 28.77(C13), 28.70(3xCH(Ad)), 28.09(C10), 26.97(C12), 26.88(C11). (+)TOF-HRMS (*m/z*): calc. for $[C_{26}H_{36}ClN_3 + H]^+$ 426.2676,

found 426.2677. HPLC purity: Method I: RT 6.684, area 95.07 %; Method II: RT 8.729, area 95.02 %.

***N*[1]-(1-adamantylmethyl)-*N*[2]-(7-chloro-4-quinolinyl)-1,8-octanediamine (5).**

Compound **5** was obtained from amine **31** (93.1 mg, 0.30 mmol) and aldehyde **34** (50.0 mg, 0.30 mmol), using $NaHB(OAc)_3$ (129.0 mg, 0.61mmol). Yield 31.7 mg (23 %), as yellow oil. IR (ATR): 3277m, 3064w, 2905s, 2849s, 1610m, 1581s, 1541w, 1453m, 1368w, 1332m, 1282w, 1250w, 1137w, 878w, 851w, 806w. ^{1}H NMR (500 MHz, mixture of CD_3OD and $CDCl_3$, δ): 8.31 (d, J = 5.7, 1H, H-C(2)), 8.07 (d, J = 9.0, 1H, H-C(5)), 7.75 (d, J = 2.2, 1H, H-C(8)), 7.35 (dd, J_1 = 9.0, J_2 = 2.3, 1H, H-C(6)), 6.45 (d, J = 5.5, 1H, H-C(3)), 3.35 – 3.28 (m, 2H, H-C(9), overlapped with CH_3OD), 2.64 – 2.52 (m, 2H, H-C(16)), 2.29 (s, 2H, H-C(17)), 2.02 – 1.88 (m, 3H, 3xCH(Ad)), 1.79 – 1.60 (m, 8H, CH_2 and 3xCH_2(Ad)), 1.60 – 1.18 (m, 16H, 5xCH_2 and 3xCH_2(Ad)). ^{13}C NMR (125 MHz, mixture of CD_3OD and $CDCl_3$, δ) 151.31(C4), 150.93(C2), 148.28(C8a), 134.84(C7), 126.19(C8), 124.48(C5), 122.89(C6), 117.36(c4a), 98.13(C3), 61.76(C17), 50.27(C16), 42.59(C9), 40.20(3xCH_2(Ad)), 36.60(3xCH_2(Ad)), 32.58(C(Ad)), 29.03(CH_2), 28.95(CH_2), 28.33(3xCH(Ad)), 27.92(CH_2), 27.89(CH_2), 26.78(C13), 26.71(C12). (+)TOF-HRMS (*m/z*): calc. for $[C_{28}H_{40}ClN_3 + H]^+$ 454.2989, found 454.2987. HPLC purity: Method I: RT 6.855, area 95.01 %; Method II: RT 8.573, area 95.08 %.

***N*[1]-(1-adamantylmethyl)-*N*[2]-(7-trifluoromethyl-4-quinolinyl)-1,2-etanediamine (6).**

Compound **6** was obtained from amine **32** (120 mg, 0.47 mmol) and aldehyde **34** (77.2 mg, 0.47 mmol), using $NaHB(OAc)_3$ (199.3 mg, 0.94mmol). Yield 152.9 mg (81 %), pale yellow oil. IR (ATR): 3339m, 3080w, 2904s, 2848s, 2677w, 1591s, 1542m, 1457w, 1377m, 1328s, 1277w, 1204w, 1161m, 1131s, 1072w, 903w, 812w. ^{1}H NMR (500 MHz, CD_3OD, δ): 8.47 (d, J = 5.7, 1H, H-C(2)), 8.28 (d, J = 8.7, 1H, H-C(5)), 8.09 (d, J = 1.9, 1H, H-C(8)), 7.63 (dd, J_1 = 8.9, J_2 = 2.0, 1H, H-C(6)), 6.68 (d, J = 5.6, 1H, H-C(3)), 3.52 (t, J = 6.5, 2H, H-C(9)), 2.94 (t, J = 6.5, 2H, H-C(10)), 2.32 (s, 2H, H-C(10)), 2.01 – 1.86 (m, 3H, 3xCH(Ad)), Ad), 1.79 – 1.60 (m, 6H, 3xCH_2(Ad)), 1.56 (d, J = 2.9, 6H, 3xCH_2(Ad)). ^{13}C NMR (125 MHz, CD_3OD, δ) 151.48(C2), 151.13(C4), 146.80(C8a), 130.75 (q, J = 32.6, C7), 125.41 – 124.66 (m, C8), 124.0 (q, J = 268.7, CF_3), 122.69(C5), 120.75(C4a), 119.54 – 119.21 (m, C6), 99.41(C3), 62.09(C11), 48.14(C10), 41.59(C9), 40.37(3xCH_2(Ad)), 36.70(3xCH_2(Ad)), 32.92(C(Ad)), 28.41(3xCH(Ad)).). (+)TOF-HRMS (*m/z*): calc. for $[C_{23}H_{28}F_3N_3 + H]^+$ 404.2314, found 404.2294. HPLC purity: Method I: RT 6.529, area 96.01 %; Method II: RT 8.137, area 95.78 %.

N^1-(1-adamantylmethyl)-N^2-(7-trifluoromethyl-4-quinolinyl)-1,3-propanediamine (7).

Compound **7** was obtained from amine **33** (120 mg, 0.44 mmol) and aldehyde **34** (73.2 mg, 0.44 mmol), using $NaHB(OAc)_3$ (188.93 mg, 0.94mmol). Yield 112.0 mg (59 %), as pale yellow oil. IR (ATR): 3358m, 3079w, 2903s, 2848s, 2677w, 1597s, 1573m, 1557w, 1454w, 1373m, 1327s, 1282m, 1244w, 1201w, 1152s, 1113s, 1066w, 900w, 858w, 823w, 803w, 739w. ^{1}H NMR (500 MHz, CD_3OD, δ): 8.48-8.43 (m, 1H, H-C(2)), 8.31-8.26 (m, 1H, H-C(5)), 8.08 (bs, 1H, H-C(8)), 7.65 – 7.56 (m, 1H, H-C(6)), 6.67-6.61 (m, 1H, H-C(3)), 3.49-3.41 (m, 2H, H-C(9)), 2.79-2.71 (m, 2H, H-C(11)), 2.30-2.24(m, 2H, H-(C11)), 2.03 – 1.87 (m, 5H, H-C(10), 3xC*H*(Ad)), 1.79 – 1.58 (m, 6H, Ad), 1.54 (bs, 6H, Ad). ^{13}C NMR (125 MHz, CD_3OD, δ) 151.46(C2), 151.07(C4), 146.81(C8a), 130.70 (q, J = 32.5, C7), 124.94 (q, J = 3.7, C8), 123.95 (q, J = 282.5, CF_3), 122.82(C5), 120.73(C4a), 119.30 – 119.12 (m, C6), 99.27(C3), 62.32(C12), 48.39(C11), 41.07(C9), 40.37(3xCH_2(Ad)), 36.65(3xCH_2(Ad)), 32.75(C(Ad)), 28.30(3xCH(Ad)), 26.97C(10). (+)TOF-HRMS (*m/z*): calc. for $[C_{24}H_{30}F_3N_3 + H]^+$ 418.2470, found 418.2465. HPLC purity: Method I: RT 6.654, area 96.16 %; Method II: RT 8.246, area 96.34 %.

N^1-(1-adamantylethyl)-N^2-(7-chloro-4-quinolinyl)-1,2-ethanediamine (8) and N^1,N^1-di(1-adamantylethyl)- N^2-(7-chloro-4-quinolinyl)-1,2-ethanediamine (9) [73].

Compound **8** and **9** were obtained from amine **26** (110 mg, 0.50 mmol) and aldehyde **35** (88.4 mg, 0.50 mmol), using $NaHB(OAc)_3$ (210.3 mg, 0.99 mmol). Yield 23mg (12 %) **8** and 51.0 mg (19%) **9**, as pale yellow oils. **8**: IR (ATR): 3300m, 3114m, 3068m, 2904s, 2846s, 2677w, 1612m, 1583s, 1543m, 1451m, 1430w, 1402w, 1372w, 1332w, 1282w, 1250w, 1142w, 1081w, 902w, 877w, 850w, 808w. ^{1}H NMR (500 MHz, CD_3OD, δ): 8.40 – 8.38 (m, 1H, H-C(2)), 8.14 - 8.06 (m, 1H, H-C(5)), 7.81 – 7.75 (m, 1H, H-C(8)), 7.45 – 7.38 (m, 1H, H-C(6)), 6.62 – 6.55 (m, 1H, H-C(3)), 3.61 (t, J = 6.3, 2H, H-C(9)), 3.11 (t, J = 6.3, 2H, H-C(10)), 2.87 – 2.78 (m, 2H, H-(C11)), 1.92 (d, J = 2.1, 3H, 3xCH(Ad)), 1.77 – 1.60 (m, 6H, 3xCH_2(Ad)), 1.52 (d, J = 3.3 Hz, 6H, 3xCH_2(Ad)), 1.41 – 1.33 (m, 2H, H-C(12)). ^{13}C NMR (125 MHz, CD_3OD, δ) 151.13(C4), 150.90(C2), 148.03(C8a), 135.12 (C7), 126.07 (m, C8), 124.83 (C5), 122.98 (C6), 117.40(C4a), 98.37(C3), 46.35(C10), 43.37(C9), 41.95(3xCH_2(Ad)), 32.92(C(Ad)), 40.60 (C11), 36.58(3xCH_2(Ad)), 31.32(C12), 28.57(3xCH(Ad)). (+)TOF-HRMS (*m/z*): calc. for $[C_{23}H_{30}ClN_3 + H]^+$ 384.2207, found 384.2221. HPLC purity: method III: RT 1.986, area 95.97 %; method IV: RT 3.308, area 97.79 %. **9**: IR (ATR): 3241w, 3109w, 3067w, 3010w, 2898s, 2843s, 2675w, 1612m, 1577s, 1547m, 1492w, 1471w, 1450m, 1377w, 1331w, 1310w, 1280w, 1253w, 1199w, 1103s, 1080w, 1013w, 965w, 910w, 880w, 849w, 811w. ^{1}H NMR (500 MHz, mixture of CD_3OD and $CDCl_3$, δ): 8.35 (d, J = 5.6, 1H, H-C(2)), 7.90 (dd, J_1 = 9.0, J_2 = 1.9, 1H, H-C(5)), 7.80 (d, J = 2.2, 1H, H-C(8)), 7.37 (dd, J_1 = 9.1, J_2 = 2.2, 1H, H-C(6)), 6.42 (d, J = 5.6, 1H, H-C(3)), 3.37 (t, J = 6.0, 2H, H-C(9)), 2.79 (t, J = 6.0, 2H, H-C(10)), 2.60 – 2.50 (m, 4H,

H-(C11) and H-(C11')), 1.95 – 1.82 (m, 6H, 6H, 6xCH(Ad)), 1.73 – 1.54 (m, 12, 6xCH_2(Ad)), 1.46 (d, J = 2.8 Hz, 12H, 6xCH_2(Ad)), 1.28 – 1.18 (m, 4H, H-(C12) and H-(C12)). ^{13}C NMR (125 MHz, mixture of CD_3OD and $CDCl_3$, δ) 150.91(C2), 150.80(C4), 148.03(C8a), 135.29 (C7), 126.66 (m, C8), 125.19 (C5), 122.27 (C6), 117.32(C4a), 98.56(C3), 51.51(C10), 43.43(C9), 42.34(6xCH_2(Ad)), 40.04(C(Ad)), 39.89 (C11) and (C11'), 36.86(6xCH_2(Ad)), 31.61 (C12) and (C12'), 28.51(6xCH(Ad)). (+)TOF-HRMS (*m/z*): calc. for [$C_{35}H_{48}ClN_3$ + H]$^+$ 546.3615, found 546.3633. HPLC purity: Method I: RT 7.273, area 95.17 %; Method II: RT 9.427, area 96.03 %.

N^1,N^1-bis[2-(1-adamantyl)ethyl]-N^2-(7-chloroquinolin-4-yl)butane-1,4-diamine (10)

Compound **10** were obtained from amine **28** (300.0 mg, 1.20 mmol) and aldehyde **35** (214.0 mg, 1.20 mmol), using $NaHB(OAc)_3$ (509.0 mg, 2.40 mmol). Yield 55 mg (8%) as colourless powder, softens at 84-86 °C. IR (ATR): 2897s, 2843m, 1611w, 1579s, 1538w, 1448m, 1367m, 1344w, 1331w, 1136m, 1098w, 877w, 852w, 805m cm^{-1}. ^{1}H-NMR (200 MHz, $CDCl_3$, δ): 8.49 (d, 1H, J = 5.6 Hz, H-C(2)), 7.92 (d, 1H, J = 1.7 Hz, H-C(8)), 7.79 (d, 1H, J = 9.0 Hz, H-C(5)), 7.30 (dd, J_1 = 9.0, J_2 = 1.7, H-C(6)), 6.37 (d, 1H, J = 5.1 Hz, H-C(3)), 6.11 (bs, 1H, -N*H*), 3.38-3.24 (m, 2H, H-C(9)), 2,58-2,40 (m, 6H, H-C(12), H-C(13) and H-C(13')), 2,04-1,42 [m, 34H, H-C(10), H-C(11), 6xCH(Ad), 6xCH_2(Ad), 6xCH_2(Ad), 6xCH_2(Ad)], 1,30-1,16 (m, 4H, H-C(14) and H-C14')). ^{13}C-NMR (50 MHz, $CDCl_3$, δ): 151.83, 150.08, 149.05, 134.61, 128.34, 124.79, 121.79, 117.31, 98.81, 53.11, 47.34, 43.01, 42.35, 39.69, 36.96, 31.63, 28.48, 26.46, 24.82. **(+)**ESI-HRMS (m/z (%)): 574.39419 ([M+H]$^+$, 100), calculated 574.39225 (+3.38 ppm). HPLC purity: method III: RT 7.973 min., area 98.88%; method IV: RT 10.566 min., area 95.79%.

***N^1*,*N^1*-di(1-adamantylethyl)- *N^2*-(7-chloro-4-quinolinyl)-1,5-pentanediamine (11).**

Compound **11** were obtained from amine **29** (145.0 mg, 0.55 mmol) and aldehyde **35** (98.0 mg, 0.55 mmol), using using $NaHB(OAc)_3$ (233.0 mg, 1.10 mmol). Yield 35.6mg (11 %) **11** as pale yellow oils. IR (ATR): 3263w, 2896m, 2843m, 1610w, 1577s, 1539w, 1367m, 1344w, 1330w, 1263w, 1202w, 1166w, 1136w, 1098w, 1078w, 1014w, 987w, 970w, 899w, 877w, 850w, 804w. ^{1}H NMR (500 MHz, $CDCl_3$, δ): 8.53 (d, J = 5.4, 1H, H-C(2)), 7.95 (d, J = 2.2, 1H, H-C(8)), 7.70 (d, J = 8.9, 1H, H-C(5)), 7.36 (dd, J_1 = 8.9, J_2 = 2.2, 1H, H-C(6)), 6.41 (d, J = 5.4, 1H, H-C(3)), 5.30 (bs, N-H), 3.33 (td, J_1 = 7.0, J_2 = 5.1 Hz, 2H, H-C(9)), 2.53 – 2.40 (m, 6H, H-C(13), H-C(14) and H-C(14')), 1.96 – 1.89 (m, 6H, 6xCH(Ad)), 1.79 (p, J = 7.1 Hz, 2H, H-C(10)), 1.73 – 1.58 (m, 12H, 6xCH_2(Ad)), 1.58 – 1.42 (m, 16H, H-C(11), H-C(12) and 6CH_2(Ad)), 1.25 – 1.18 (m, 4H) H-C(15) and H-C(15')). ^{13}C NMR (125 MHz, $CDCl_3$, δ) 152.08 (C2), 149.73 (C8a), 134.81 (C7), 128.87 (C8), 125.23 (C6), 120.96 (C5), 117.16 (C4a), 99.04 (C3), 53.71 (C13), 47.56 (C14 and C14'), 43.11 (C9), 42.55 (6xCH_2(Ad)), 40.22 (C15 and C15'), 37.16 (6xCH_2(Ad)), 31.83 (C(Ad)), 28.66 (6xCH(Ad) and

C(10)), 26.72 [C(12) or C(11)], 25.03 [C(11) or C(12)]. (+)TOF-HRMS (*m/z*): calc. for [$C_{38}H_{54}ClN_3$ + H]$^+$ 588.4085, found 588.4063. HPLC purity: Method I: RT 7.273, area 95.17 %; Method II: RT 9.427, area 96.03 %.

Scheme S2

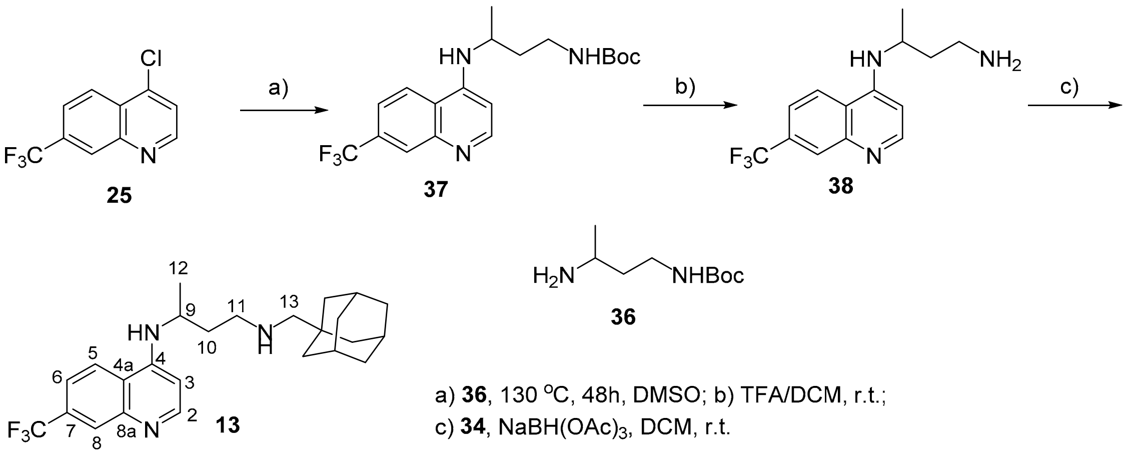

a) **36**, 130 °C, 48h, DMSO; b) TFA/DCM, r.t.; c) **34**, $NaBH(OAc)_3$, DCM, r.t.

***tert*-butyl (3-((7-(trifluoromethyl)quinolin-4-yl)amino)butyl)carbamate (37).**

Solution of **25** (96.7 mg, 0.42 mmol) and **36** (117.9 mg, 0.63 mmol) in DMSO (0.2 mL) was stirred for 6 hours at 130 °C in inert atmosphere (Ar). The reaction mixture was cold to room temperature., cold water was added and the crude product was extracted with DCM (3x15 mL). Combined organic layers were washed once with brine, dried over anhydrous Na_2SO_4 and solvent was evaporated under reduced pressure. The product **37** was isolated after chromatographic purification (dry-flash, SiO_2, gradient hexane-ethyl acetate). Yield, 80.3mg (50%). IR (ATR): 3320m, 3199m, 2969m, 2931m, 1681s, 1590s, 1546s, 1456w, 1435w, 1373m, 1326s, 1285s, 1253w, 1231w, 1157s, 1125s, 1019w, 955w, 903w, 867w, 812w, 776w, 739w. ^{1}H NMR (500 MHz, $CDCl_3$, δ): 8.59 (d, J = 5.4, 1H, H-C(2)), 8.25 (s, 1H, H-C(8)), 7.99 (d, J = 8.7, 1H, H-C(5)), 7.57 (dd, J_1 = 8.6, J_2 = 1.9, 1H, H-C(6)), 6.48 (d, J = 5.4, 1H, H-C(3)), 5.56 (bs, N-H), 4.83 (bs, 1H, N-H), 3.80 (hep, 1H, H-C(9)), 3.40 – 3.16 (m, 2H, H-C(11)), 1.96 – 1.83 (m, 2H, H-C(10)), 1.45 (s, 9H, *t*-Bu), 1.36 (d, 3H, CH_3-C(9)). ^{13}C NMR (125 MHz, $CDCl_3$, δ): 156.21(CO), 152.09(C2), 148.83(C4), 147.83 (C8a), 131.42 – 130.31 (m, C7), 127.52 (m, C8), 124.0 (q, J = 270.0, CF_3), 121.19 (C6), 120.62(C4a), 119.96 (C5), 100.05(C3), 79.75 (*C*-$(CH_3)_3$), 46.53 (C9), 37.45 (C11), 36.75 (C10), 28.35 (3xMe), 20.07 (C12).

***N*^{2}-(7-(trifluoromethyl)quinolin-4-yl)butane-1,3-diamine (38).**

Into the solution of **37** (134 mg, 0.35 mmol) in DCM (3.2 mL) TFA was added (0.32 mL) and the reaction mixture was stirred at room temperature for 5 hours. The reaction was quenched with a water solution of NaOH (2.5 M), the organic layer was separated, and the water was extracted with DCM (2x10 mL). Combined organic layers were washed with brine, dried over anhydrous Na_2SO_4 and

solvent was evaporated under reduced pressure. The product **38** [91.5 mg, yellow oil, contained 15% impurity (NMR purity)] was used in the next step without additional purification. ^{1}H NMR (500 MHz, CD_3OD, δ): 8.49 (d, *J* = 5.8, 1H, H-C(2)), 8.43 (d, *J* = 8.8, 1H, H-C(5)), 8.10 (bs, 1H, H-C(8)), 7.68 (dd, J_1 = 8.9, J_2 = 2.1, 1H, H-C(6)), 6.75 (d, *J* = 5.8, 1H, H-C(3)), 4.04 (hep, 1H, H-C(9)), 3.15 – 3.01 (m, 2H, H-C(11)), 2.22 – 1.89 (m, 2H, H-C(10)), 1.41 (d, 3H, CH_3-C(9)). ^{13}C NMR (125 MHz, CD_3OD, δ): 151.02(C4), 150.47(C2), 147.83 (C8a), 131.85 – 131.74 (m, C7), 124.24-123.82 (m, C8), 123.19 (C5), 123.87 (q, *J* = 270.0, CF_3), 120.62(C4a), 119.79 (C6), 99.77(C3), 46.09 (C9), 36.57 (C11), 33.57 (C10), 18.44 (C12).

***N*^{1}-((adamantan-1-yl)methyl)-*N*^{2}-(7-(trifluoromethyl)quinolin-4-yl)butane-1,3-diamine (13):** Applying the general procedure for reductive amination, derivative **13** was obtained from amine **38** (91.0 mg, 0.32 mmol) and aldehyde **34** (56.9 mg, 0.34 mmol), using $NaHB(OAc)_3$ (136.2 mg, 0.64 mmol). Yield 38.1 mg (23 %) **13** as pale yellow oil. IR (ATR): 3271w, 3078w, 2900s, 2845s, 1583s, 1541s, 1451w, 1376m, 1325s, 1277w, 1203w, 1156s, 1125s, 1070w, 902w, 877w, 854w, 810w. ^{1}H NMR (500 MHz, CD_3OD, δ): 8.45 (d, *J* = 5.6, 1H, H-C(2)), 8.37 (d, *J* = 8.7, 1H, H-C(5)), 8.08 (s, 1H, H-C(8)), 7.61 (d, J_1 = 8.9, J_2 = 1.9, 1H, H-C(6)), 6.70 (d, *J* = 5.7, 1H, H-C(3)), 3.94 (hep, 2H, H-C(9)), 2.76 (t, *J* = 7.3, 2H, H-C(11)), 2.28 (s, 2H, H-C(13)), 2.02 – 1.84 (m, 5H, H-C10 and 3xCH(Ad)), 1.76 – 1.56 (m, 6, 3xCH_2(Ad)), 1.50 (bs, 6H, 3xCH_2(Ad)), 1.36 (d, 3H, CH_3-C(9)). ^{13}C NMR (125 MHz, CD_3OD δ): 152.61(C2), 151.60(C4), 148.14(C8a), 132.49 – 131.31 (m, C7), 126.21 – 125.91 (m, C8), 124.12 (C5), 124.07 (C4a), 123.04 (q, J = 270, CF_3), 120.32 (C6), 100.71(C3), 63.32 (H-C(13)), C11 and C9 are overlaped with CH_3OD, , 43.39 (3x*C*H_2(Ad)), 37.72 (3xCH_2(Ad)), 35.65 (C(Ad)), 39.89 (C10), 29.47(3xCH(Ad)), 19.99 (*C*H_3-C(9)). (+)TOF-HRMS (*m/z*): calc. for $[C_{25}H_{32}F_3N_3 + H]^+$ 432.2627, found 432.2645. HPLC purity: Method I: RT 6.833, area 96.16 %; Method II: RT 8.312, area 95.53 %.

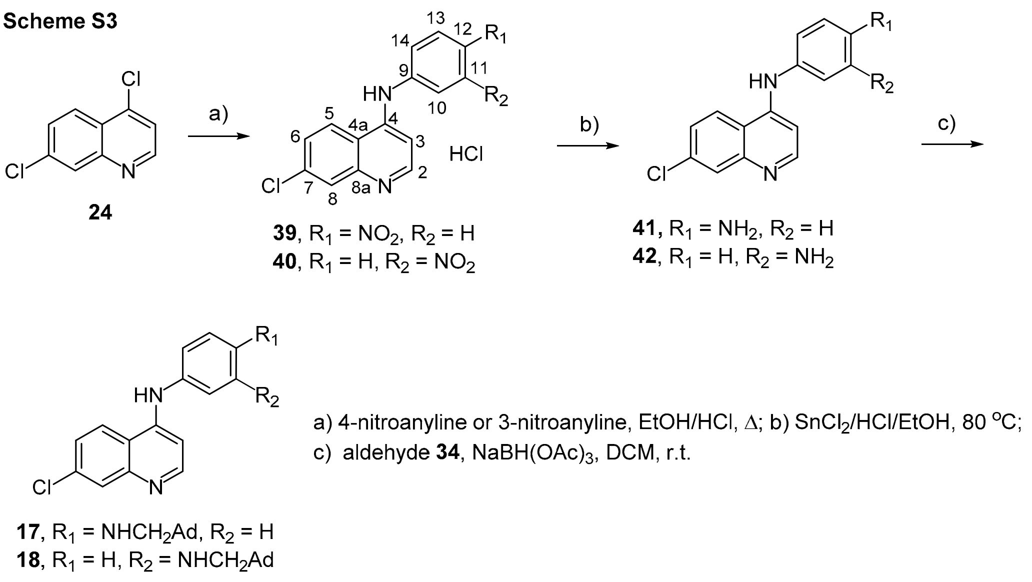

7-chloro-*N*-(4-nitrophenyl)quinoline-4-amine hydrochloride 39 [76]:

Solution of **24** (250.0 mg, 1.26 mmol) and 4-nitro-aniline (174.3 mg, 1.26 mmol), in ethanol (10 mL) with a catalytic amount of 37% hydrochloric acid were refluxed for 2 h. The reaction mixture cooled to room temperature, and the precipitated solid was filtered off, washed with cold MeOH (3 × 5 mL), dried under reduced pressure and afford **39** as a hydrochloride salt. Yield 312.6 mg [contained 7% of starting 4-nitro-aniline (NMR purity)].

IR (ATR): 3195m, 3087m, 3003m, 1619m, 1581s, 1542s, 1501s, 1447m, 1377w, 1344s, 1299m, 1238m, 1209m, 1177m, 1093m, 1000w, 917w, 854w, 811w, 744.1w, 693w, 647w. ^{1}H NMR (500 MHz, DMSO-d6, δ): 11.46 (bs, 1H, H-N), 8.68 (d, J = 7.0, 1H, H-C2), 8.36 (d, J = 8.5, 2H, H-C(11) and H-C(11')), 8.22 (d, J = 2.2, 1H, H-C(8)), 7.89 (dd, J_1 = 9.2, J_2 = 2.1, 1H, H-C(6)), 7.78 (d, J = 8.6, 2H, H-C(10) and H-C(10')), 7.21 (d, J = 6.8 Hz, 1H, H-C(3)). ^{13}C NMR (125 MHz, CD_3OD δ): 154.12 (C2), 145.22 (C8a), 144.78 (C9), 144.40 (C4), 139.93 (C12), 139.01 (C7), 128.21 (C8), 126.90 (C6), 125.79 (C11 and C11'), 124.85 (C10 and C10'), 120.04 (C5), 117.42(C4a), 102.62 (C3).

7-chloro-*N*-(3-nitrophenyl)quinoline-4-amine hydrochloride 40:

Following the procedure for the synthesis derivative **39**, derivative **40** was obtained from **24** (250.0 mg, 1.26 mmol) and 3-nitro-aniline (174.3 mg, 1.26 mmol). Yield 377.9 mg (89%). IR (ATR): 3195w, 3072s, 2980s, 2884s, 2698s, 1602s, 1536s, 1518s, 1444s, 1354s, 1237m, 1210m, 1171m, 1094m, 1060w, 972w, 909w, 886w, 851w, 814m, 776w, 736w, 719w. ^{1}H NMR (500 MHz, mixture of DMSO-d6 and CD_3OD, δ): 8.75 – 8.64 (m, 1H, H-C(2)), 8.60 – 8.49 (m, 1H, H-C5), 8.35 – 8.29 (m, 1H, H-C(10)), 8.26 – 8.19 (m, 1H, H-C(12)), 8.08 – 8.00 (m, 1H, H-C(8)), 7.96 (m, 1H, H-C(6)), 7.89 – 7.76 (m, 2H, H-C(14) and H-C13)). ^{13}C NMR (125 MHz, CD_3OD δ): 157.08, 151.07, 146.05, 141.37, 141.19, 140.48, 133.49, 130.06, 127.92, 124.14, 122.15, 121.60, 118.46, 102.94.

***N*¹-(7-chloroquinolin-4-yl)benzene-1,4-diamine 41 [76]:**

Into ice-cold suspension of **39** (35.0 mg, 0.12 mmol) and $SnCl_2$ (110.7 mg, 0.58 mmol) in EtOH (1.3 mL), 36% HCl was added in drops (30 μL) afterwards reaction mixture was warmed to 80 ºC and stirred 1 hour. After cooling at r.t. water solution of Na_2CO_3 (1M) was added, and the mixture was extracted with ethyl-acetate (3x 10mL). Combined organic layers were washed with brine and dried over Na_2SO_4 anh. The solvent was removed under reduced pressure and the product was isolated after column chromatography (dry-flash, SiO2, gradient DCM/MeOH) as a pale yellow oil. Yield, 22.5 mg (71%). IR (ATR): 3377s, 3194s, 1613s, 1578s, 1541s, 1515s, 1440m, 1416m, 1374m, 1332m, 1251m, 1201w, 1171w, 1118w, 1084w, 944w, 915w, 868w, 824w, 767w, 760w. ^{1}H NMR (500 MHz, CD_3OD, δ): 8.24 – 8.13 (m, 2H, H-C2 and H-C(5)), 7.75 (d, *J* = 2.2, 1H, H-C(8)), 7.40 (dd, J_1 = 9.0, J_2 = 2.1, 1H, H-C(6)), 7.09 – 6.98 (m, 2H, H-C(11) and H-C(11')), 6.81 – 6.72 (m, 2H, H-C(10) and H-C(10')), 6.53 (d, *J* = 5.7 Hz, 1H, 1H, H-C(3)). ^{13}C NMR (125 MHz, CD_3OD δ): 151.94 (C4), 150.50 (C2), 148.25 (C8a), 146.30 (C4), 135.65 (C8), 129.40C(5), 126.44 (C11 and C11'), 125.94 (C6), 125.23 (C11 and C11'), 125.23 (C10 and C10'), 117.67 (C4a), 116.08 (C11 and C11'), 100.41 (C3).

***N*¹-(7-chloroquinolin-4-yl)benzene-1,3-diamine 42:**

Following the procedure for synthesis of derivative **41**, compound **42** was obtained from **40** (150 mg, 0.45 mmol) and $SnCl_2$ (423.0 mg, 2.23 mmol). Yield: 88.8 mg (74%)., as pale yellow oil. IR (ATR): 3472m, 3380m, 3171m, 3053m, 3009m, 2937m, 1610s, 1566s, 1540s, 1491s, 1457m, 1418m, 1371m, 1328m, 1282m, 1246m, 1198m, 1160m, 1115w, 1082w, 993w, 960w, 906w, 882w, 854w, 820w, 761w. ^{1}H NMR (500 MHz, mixture of $CDCl_3$ and CD_3OD, δ): 8.35 (d, *J* = 5.6, 1H, H-C2), 8.28 (d, *J* = 9.1, 1H, H-(C5)), 7.86 (d, *J* = 2.2, 1H), H-C(8)), 7.49 (dd, J_1 = 9.0, J_2 = 2.2, 1H, H-C(6)), 7.18 (t, *J* = 7.9, 1H, H-C(10)), 6.95 (d, *J* = 5.7, 1H, H-(C3)), 6.75 (t, *J* = 2.1, 1H, H-C(10)), 6.69 (dd, J_1 = 7.8, J_2 = 2.0, 1H, H-C(14)), 6.62 (dd, J_1 = 8.0, J_2 = 2.1, 1H, H-C(12)). ^{13}C NMR (125 MHz, mixture of $CDCl_3$ and CD_3OD δ): 151.53 (C4), 151.38 (C2), 149.96 (C8a), 149.19 (C7), 141.29 (C9), 136.70(C11), 130.90 (C13), 126.93 (C8), 126.43 C(6), 124.48 (C5), 119.07 (C4a), 114.05 (10), 113.33 (C14), 111.25 (C12), 102.56 (C3).

***N¹*-((adamantan-1-yl)methyl)-*N²*-(7-chloroquinolin-4-yl)benzene-1,4-diamine (17):**

Applying the general procedure for reductive amination, derivative **17** was obtained from amine **41** (52.6 mg, 0.19 mmol) and aldehyde **34** (32.0 mg, 0.19 mmol), using $NaHB(OAc)_3$ (82.6 mg, 0.39 mmol). Yield: 29.3 mg (36 %), pale yellow oil. IR (ATR): 3420w, 3277w, 3192w, 3095w, 3051w, 2898s, 2844s, 1613s, 1581s, 1517s, 1449m, 1425m, 1370m, 1315m, 1283m, 1169m, 1116m, 914w,

867w, 819w, 777w. ^{1}H NMR (500 MHz, mixture of $CDCl_3$ and CD_3OD, δ): 8.28 (d, J = 5.8, 1H, H-C(2)), 8.19 (d, J = 8.9, 1H, H-C(5)), 7.89 (bs, 1H, H-C(8)), 7.43 (dd, J_1 = 9.0, J_2 = 2.2, 1H, H-C(6)), 7.13 – 7.05 (m, 2H, H-C(11) and H-C(11')), 6.78 – 6.69 (m, 2H, H-C(10) and H-C(10')), 6.61 (d, J = 5.8, 1H, H-C(3)), 2,83 (s, 2H, H-C(13)), 2.06 – 1.97 (m, 3H, 3xCH(Ad)), 1.83 – 1.67 (m, 6H, 3xCH_2(Ad)), 1.67 – 1.60 (m, 6H, 3xCH_2(Ad)). ^{13}C NMR (125 MHz, CD_3OD δ): 152.04 (C4), 150.65 (C2), 148.46 (C8a), 148.31 (C7), 135.85 (C9), 127.80 (C12), 126.68 (C10 and C10'), 126.31 (C8), 125.49 (C5), 123.28 (C6), 117.73 (C4a), 113.35 (C11 and C11'), 100.69 (C3), 56.64 C(13), 40.77 (3xCH_2(Ad)), 37.16 (3xCH_2(Ad)), 34.22 (C(Ad)), 28.64 (3xCH(Ad)). (+)TOF-HRMS (*m/z*): calc. for $[C_{26}H_{28}ClN_3 + H]^+$ 418.2050, found 418.2055. HPLC purity: Method I: RT 8.408, area 95.13 %; Method II: RT 9.757, area 95.94 %.

***N¹*-((adamantan-1-yl)methyl)-*N²*-(7-chloroquinolin-4-yl)benzene-1,3-diamine (18):**

Applying the general procedure for reductive amination, derivative **18** was obtained from amine **42** (49.1 mg, 0.18 mmol) and aldehyde **34** (29.9 mg, 0.18 mmol), using $NaHB(OAc)_3$ (77.1 mg, 0.36 mmol). Yield: 37.2 mg (49 %), pale yellow oil. IR (ATR): 3374s, 3073m, 2903s, 2847s, 2658m, 1613s, 1588s, 1544s, 1496s, 1450s, 1370m, 1314m, 1245w, 1215w, 1192w, 1163w, 1135m, 1099m, 1056m, 992w, 898w, 844w, 817w, 770w. ^{1}H NMR (500 MHz, mixture of $CDCl_3$ and CD_3OD, δ): 8.40 – 8.29 (m, 2H, H-C(2) and H-C(5)), 8.01 (bs, 1H, H-C(8)), 7.55 (d, J = 9.3, 1H, H-C(6)), 7.20 (t, J = 7.9, 1H, H-C(10)), 6.89 (d, 2H, H-C(3)), 6.66 – 6.54 (m, 3H, H-C(12), H-C(13) and H-C(14)), 2.80, (bs, H-C(15)), 2.01 (bs, 3H, 3xCH(Ad)), 1.81 – 1.64 (m, 6H, 3xCH_2(Ad)), 1.64 – 1.56 (m, 6H, 3xCH_2(Ad)). ^{13}C NMR (125 MHz, CD_3OD δ): 153.51 (C4), 150.92 (C2), 144.11 (C8a), 138.60 (C7), 138.12 (C11 and C9), 130.12 (C5), 127.13 (C6), 124.42 C(133), 121.30 (C4a), 115.87 (C14), 111.71 (C6), 110.42 (C3), 108.72 (C12), 100.57 (C10), 55.95 C(13), 40.54 (3xCH_2(Ad)), 36.91 (3xCH_2(Ad)), 33.92 (C(Ad)), 28.25 (3xCH(Ad)). (+)TOF-HRMS (*m/z*): calc. for $[C_{26}H_{28}ClN_3 + H]^+$ 418,2050, found 418,2059. HPLC purity: Method I: RT 7.246, area 98.49 %; Method II: RT 9.408, area 97.05 %.

Scheme S4

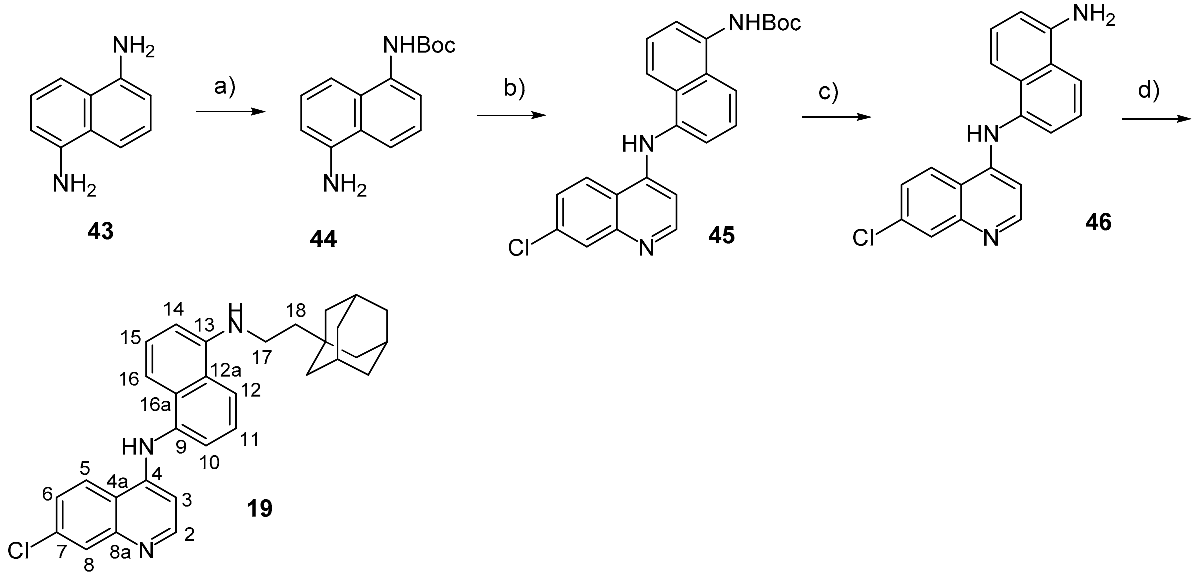

a) Boc_2O, DMF, 50 °C; b) **24**, EtOH, Δ; c) TFA/DCM, r.t.; d) **35**, $NaBH(OAc)_3$, DCM, r.t.

***tert*-butyl (5-amino-1-naphthyl)carbamate (44)**

Into the solution of 1,5-diaminonaphtalene (**43**, 25 mg, 0.16 mmol) in DMF (0.63 mL) solution of Boc_2O 34.5 mg, 0.16 mmol) in DMF (0.315 mL) was added in drops and the reaction mixture was stirred at 50 ºC for 3 days [77]. The reaction was quenched with NH_4Cl and Et_3N, the solvent was removed under reduced pressure. Remind oil was dissolved in MeOH, distilled water was added, the mixture was cooled in the ice bath (water/ice) and the amorphous solid was separated by filtration and dried under reduced pressure and the product **44** was obtained after chromatographic purification (dry-flash, SiO_2, gradient hexane/EA). Yield 11.7 mg (29%) as amorphous solid. IR (ATR): 3451s, 3373s, 3321s, 3076w, 2976s, 2939m, 2877w, 1723s, 1624s, 1595w, 1540s, 1502s, 1429s, 1391m, 1366m, 1343m, 1290w, 1236s, 1151s, 1069m, 1022m, 876m, 853m, 761s, 717w, 618m, 504w, 444w. ^1H NMR (500 MHz, $CDCl_3$, δ): 7.89 (d, J = 7.5, 1H, H-C(4)), 7.54 (d, J = 8.4, 1H, H-C(8)), 7.38 (t, J = 8.1, 1H, H-C(2)), 7.31 – 7.24 (m, 2H, H-C(7) and H-C(3)), 6.81 (bs, 1H, -NH), 6.74 (dd, J_1 = 5.9, J_2 = 2.5, 1H, H-C(6)), 4.34 (bs, 2H, NH_2), 1.51 (s, 9H, C-(CH_3)$_3$). ^{13}C NMR (126 MHz, $CDCl_3$, δ): 154.50 (C=O), 143.93 (C5), 134.53 (C4a), 128.27 (C8a), 127.60 (C3), 125.83 (C7), 125.27 (C8), 119.41 (C1), 117.95 (C2), 112.01 (C4), 110.92 (C6), 81.68 (*C*-(CH_3)$_3$), 29.48 (C-(*C*H_3)$_3$).

***tert*-butyl {5-[(7-chloroquinolin-4-yl)amino]-1-naphthyl}carbamate (45)**

Suspension of **24** (103.6 mg, 0.52 mmol) and **44** (135.2 mg, 0.52 mmol) in EtOH (5 mL) was stirred at the boiling temperature for 48h. The mixture was cooled to room temperature, the product was isolated by filtration and dried under reduced pressure. Yield: 119.8 mg, 54%. Obtained product was used in the next reaction without additional purification. IR (ATR): 3476w, 3169s, 3090m, 3054s, 2975s, 2876m, 2766s, 1986w, 1713s, 1630s, 1606s, 1562m, 1538s, 1498s, 1446s, 1414s, 1365s,

1333m, 1244s, 1207m, 1164s, 1101w, 1080m, 1048w, 1025w, 957w, 913w, 886m, 819m, 791s, 688m, 602m, 580w, 468,w 421w. ^{1}H NMR (500 MHz, mixture of CD_3OD and $CDCl_3$, δ): 8.72 (d, *J* = 9.1, 1H, H-C(2)), 8.25 – 8.20 (m, 2H, H-C(16) and H-C(5)), 7.99 (d, *J* = 2.0, 1H, H-C(8)), 7.82 (dd, J_1 = 9.1, J_2 = 2.1, 1H, H-C(6)), 7.75 – 7.65 (m, 3H, H-C(10)), H-C(14) and H-C(12)), 7.62 (dd, J_1 = 7.3, J_2 = 1.2, 1H, H-C(15)), 7.51 (dd, J_1 = 8.5, J_2 = 7.5, 1H, H-C(11)), 6.29 (d, *J* = 7.1, 1H, H-C(3)), 1.57 (s, 9H, C-(C*H*$_3$)$_3$). (126 MHz, mixture of CD_3OD and $CDCl_3$ δ): 157.37, 155.20, 142.64, 140.38, 139.30, 134.82, 132.85, 130.13, 129.89, 128.14, 127.07, 125.65, 125.03, 124.99, 123.93, 122.27, 119.36, 118.92, 115.71, 100.88, 80.20 (*C*-(CH$_3$)$_3$), 27.57 (C-(*C*H$_3$)$_3$).

***N*-(7-chloroquinolin-4-yl)naphthalene-1,5-diamine (46)**

Into the solution of **45** (105.5 mg, 0.25 mmol) in DCM (2.1 mL) TFA was added (0.21 mL) and the reaction mixture was stirred at room temperature for 5 hours. The reaction was quenched with a water solution of NaOH (2.5 M), the organic layer was separated, and the water was extracted with DCM (2x10 mL). Combined organic layers were washed with brine, dried over anhydrous Na_2SO_4 and solvent was evaporated under reduced pressure. The product **46** was obtained after chromatographic purification (dry-flash, SiO_2, gradient DCM/MeOH). Yiels 63.7 mg (79%), yellow oil. IR (ATR): 3390m, 3308m, 3206m, 3051m, 2921m, 2851m, 1903w, 1636m, 1609m, 1570s, 1512s, 1472m, 1450m, 1472m, 1409m, 1370s, 1324m, 1280m, 1254w, 1212w, 1162w, 1118m, 1077m, 942w, 885m, 848w, 828m, 809s, 772s.639w, 572w. ^{1}H NMR (500 MHz, mixture of CD_3OD and $CDCl_3$, δ): 8.27 (d, *J* = 9.0, 1H, H-C(5)), 8.08 (d, *J* = 5.7, H-C(2)), 7.89 (d, *J* = 8.1, 1H H-C(10)), 7.80 (d, *J* = 2.3 Hz, 1H, H-C(8)), 7.47 – 7.33 (m, 3H, H-C(6), H-C(12) and H-C(16)),7.23 (d, *J* = 8.3, 1H), H-C(11)), 7.13 (t, *J* = 7.9, 1H, H-C(15)), 6.77 (d, *J* = 7.3, 1H, H-C(17)), 6.05 (d, *J* = 5.5, 1H, H-C(3)). ^{13}C NMR (126 MHz, mixture of CD_3OD and $CDCl_3$, δ): 151.64, 150.54, 148.34, 143.44, 135.49, 135.44, 131.09, 126.96, 126.52, 125.49, 125.08, 124.29, 124.25, 123.10, 120.74, 117.47, 113.13, 110.21, 101.76.

***N*-[2-(1-adamantyl)ethyl]-*N'*-(7-chloroquinolin-4-yl)naphthalene-1,5-diamine (19)**

Applying the general procedure for reductive amination, derivative **19** was obtained from amine **46** (51.5 mg, 0.16 mmol) and aldehyde **34** (28.7 mg, 0.16 mmol), using $NaHB(OAc)_3$ (126.3 mg, 0.6 mmol). Yield 19.6 mg (25 %), pale yellow oil. IR (ATR): 3385m, 3336s, 3046w, 2892s, 2840s, 2658w, 1893w, 1604m, 1579s, 1531s, 1509s, 1467s, 1408m, 1365m, 1318s, 1277m, 1212m, 1156m, 1112s, 1072w, 850m, 876m, 813m, 776s, 644w, 606w, 555w, 479w, 432w. ^{1}H NMR (500 MHz, $CDCl_3$) δ 8.41 (d, *J* = 5.4, 1H, H-C(2)), 8.05 (d, *J* = 2.2, 1H, H-C(8)), 8.00 (d, *J* = 8.9, 1H, H-C(5)), 7.79 (d, *J* = 8.0, 1H, H-C(10)), 7.52 – 7.42 (m, 3H, H-C(6), H-C(12) and H-C(16)), 7.38 – 7.31 (m, 1H, H-C(11)), 7.27 – 7.22 (m, 1H, H-C(15), overlapped with $CDCl_3$), 6.66 (d, *J* = 7.5, 1H, H-C(17)), 6.40 (d, *J* = 5.4, 1H, H-C(3)), 3,35 – 2,25 (m, 2H, H-C(17)), 2.05 – 1.97 (m, 3H, 3xC*H*(Ad)), 1.80 –

1.65 (m, 6H, 3xCH2(Ad)), 1.65 – 1.60 (m, 6H, 3xCH2(Ad)), 1.60 – 1.55 (m, 2H, H-C(18)). ^{13}C NMR (126 MHz, $CDCl_3$, δ): 151.80, 149.40, 149.30, 144.48, 135.36, 135.25, 130.55, 128.92, 127.76, 125.98, 124.60, 124.31, 123.05, 121.21, 118.98, 117.54, 110.62, 104.80, 102.71, 43.94 (C17), 42.68 (3xCH2(Ad)), 38.90 (C18), 37.08 (3xCH2(Ad)), 32.13 (C(Ad)), 28.65 (3xCH(Ad)). (+)TOF-HRMS (*m/z*): calc. for $[C_{31}H_{32}ClN_3 + H]^+$ 482.2363, found 482.2344. HPLC purity: Method I: RT 8.904, area 95.10 %; Method II: RT 10.141, area 95.85 %.

Scheme S5

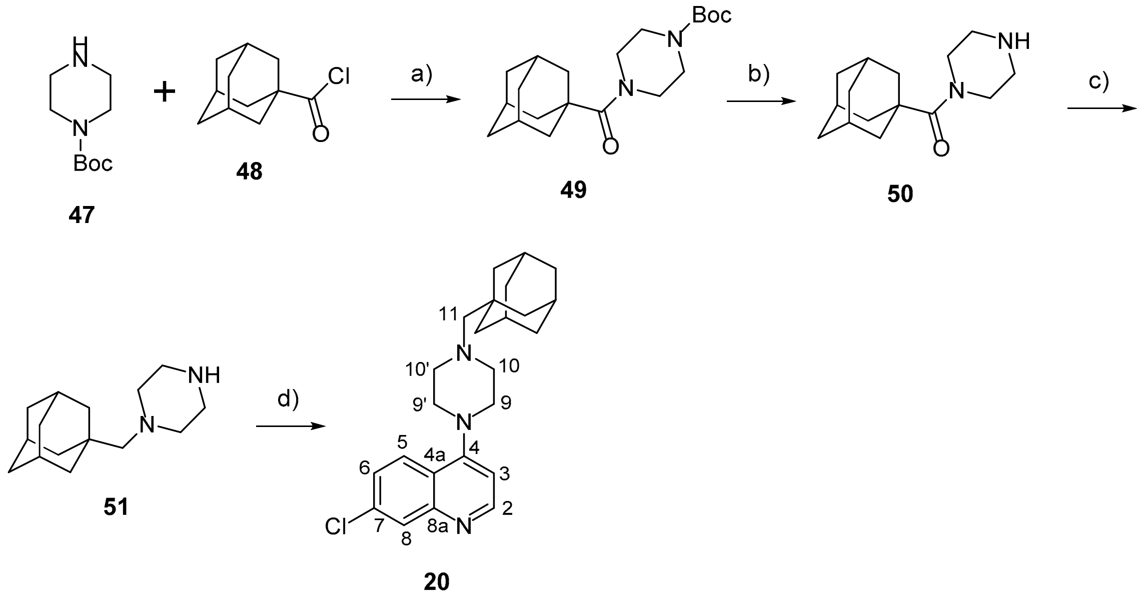

a) Et_3N, DCM, r.t.; b) TFA/DCM, r.t.; c) LAH, THF, Δ; d) 4,7-DCQ, NMP, Ar, 130 °C, 4 days

tert-butyl 4-(1-adamantylcarbonyl)piperazine-1-carboxylate (49)

Into the flame dried round bottom flask **47** (57.7 mg, 0.31 mmol) was dissolved in dry dichloromethane (0.9 mL) in the presence of Et_3N (70 μL, 0.50 mmol). The solution was cooled on the ice bath (ice/water) and the solution of **49** (47.3 mg, 0.24 mmol) in dry dichloromethane (0.3 mL) was added in drops (10 min). The mixture was stirred at room temperature overnight, saturated water solution $NaHCO_3$ (0.8 mL) was added, the mixture was transferred into a separatory funnel and layers were separated. The water layer was extracted with DCM (3x10 mL), organic layers were combined, washed with brine and dried over anhydrous Na_2SO_4. The product was obtained after chromatographic purification (dry-flash, SiO_2, gradient Hex/EA). Yield 57.0 mg (70%) as a pale-yellow oil. IR (ATR): 3064w, 3003w, 2975m, 2908s, 2852s, 2676w, 1685s, 1618s, 1450m, 1414s, 1362m, 1285m, 1238s, 1169s, 1120m, 1070m, 1046w, 1021m, 996m, 939m, 863m, 771m, 729m, 650w, 564m, 532w, 492w, 462w. ^{1}H NMR (400 MHz, $CDCl_3$, δ): 3.68 – 3.58 (m, 4H, Heq-piperazine), 3.42 – 3.35 (m, 4H, Hax- piperazine), 2.06– 1.99 (m, 3H, 3xC*H*(Ad)), 1.99 – 1.93 (m, 6H, 3xCH2(Ad)), 1.76 – 1.64 (m, 6H, 3xCH2(Ad)), 1.44 (bs, C-(C*H*3)3). ^{13}C NMR (101 MHz, $CDCl_3$,

δ): 175.98 (Ad-C=O), 154.65 (-C=O), 80.12 (-*C*(CH_3)$_3$), 45.18 (*C*$H_2$$CH_2$)$_2$NBoc), 41.69 C(Ad), 39.04 3xCH_2(Ad), 39.65 (-N(CH_2*C*H_2)$_2$NBoc), 36.56 (3xCH_2(Ad)), 36.41 (-N(CH_2*C*H_2)$_2$NBoc)), 28.40 (3xCH(Ad)), 28.36 (C-(*C*H_3)$_3$).

1-(1-adamantylcarbonyl)piperazine (50)

Into the solution of **49** (55.0 mg, 0.16 mmol) in DCM (1.4 mL) TFA was added (0.14 mL) and the reaction mixture was stirred at r.t. 5 hours. The reaction was quenched with a water solution of NaOH (2.5 M), the organic layer was separated, and the water was extracted with DCM (2x10 mL). Combined organic layers were washed with brine, dried over anhydrous Na_2SO_4 and solvent was evaporated under reduced pressure. The product **50** was used in the next reaction without additional purification. Yield: 35.4 mg (90%). IR (ATR): 3490m, 3302m, 3052w, 2906s, 2852s, 2744w, 2678w, 1617s, 1448m, 1416s, 1365w, 1342w, 1320w, 1259s, 1227s, 1181w, 1141w, 1103w, 1049w, 1023m, 831w. ^{1}H NMR (400 MHz, $CDCl_3$, δ): 3.70 – 3.57 (m, 4H, Heq-piperazine), 2.85 – 2.78 (m, 4H, Hax-piperazine), 2.04 – 1.97 (m, 3H, 3xC*H*(Ad)), 1.97 – 1.94 (m, 6H, 3xCH_2(Ad)), 1.88 (s, 1H, N-H), 1.74 – 1.62 (m, 6H, 3xCH_2(Ad)). ^{13}C NMR (101 MHz, $CDCl_3$, δ): 175.71 (C=O), 46.53 (-N(*C*$H_2$$CH_2$)$_2$NH), 46.38 (-N($CH_2$*C*$H_2$)$_2$NH)), 41.61 C(Ad)), 42.07 (C(Ad)), 39.01 (3xCH_2(Ad)), 36.61 (3xCH_2(Ad)), 28.45 (3xCH(Ad)).

1-(1-adamantylmethyl)piperazine (51)

In a flame-dried round bottom flask, under inert atmosphere (Ar) suspension of $LiAlH_4$ (61.1 mg, 1.61 mmol) in dry THF (2.8 mL) was cooled in the ice-bath (ice/water) and solution of **50** (200.0 mg, 0.8 mmol) in dry THF (4.3 mL) was added in drops, and the reaction mixture was refluxed 4 hours. The mixture was cooled to room temperature, quenched with a water solution of NaOH (2 M), the organic layer was separated, and the water was extracted with DCM (3x10 mL). Combined organic layers were washed with brine, dried over anhydrous Na_2SO_4 and solvent was evaporated under reduced pressure. The product **51** 174.7 mg yellow oil was used in the next reaction without additional purification. Yield: 174.7 mg (92%), yellow oil. IR (ATR): 3375m, 3306m, 3218m, 2904s, 2844s, 2813m, 2677w, 1628w, 1543w, 1454m, 1425w, 1361m, 1323w, 1275m, 1162m, 1129m, 1107w, 1011m, 856w, 811w, 791w, 739w, 603w. ^{1}H NMR (400 MHz, mixture of CD_3OD and $CDCl_3$, δ): 2.84 – 2.76 (m, 4H, Heq-piperazine), 2.51 – 2.38 (m, 4H, Hax-piperazine), 1.95 – 1.88 (m, 5H, -C*H*$_2$-Ad and 3xC*H*(Ad)), 1.74 – 1.58 (m, 6H, 3xCH_2(Ad)), 1.53 – 1.43 (m, 6H, 3xCH_2(Ad)). ^{13}C NMR (100 MHz, mixture of CD_3OD and $CDCl_3$, δ): 71.57 (*C*H_2-Ad), 56.18 (-N(*CH*$_2$$CH_2$)$_2$NH), 45.31 (-N($CH_2$*C*$H_2$)$_2$NH)), 40.78 (3x*C*$H_2$(Ad)), 36.99 (3x*C*$H_2$(Ad)), 34.73 (*C*(Ad)), 28.46 (3x*C*H(Ad)).

4-[4-(1-adamantylmethyl)piperazin-1-yl]-7-chloroquinoline (20)

Mixture of **24** (20.0 mg, 0.084 mmol) and **51** (16.8 mg, 0.084 mmol) in phenole (24.0 mg, 0.252 mmol) was stirred for 24 hours at 130 °C in inert atmosphere (Ar). The mixture was cooled to room temperature, dissolved in DCM and washed with water solution of NaOH (10%, 3x15 mL), once with brine and dried over Na_2SO_4 anhydrous. Compound **20** was isolated after column chromatography (dry-flash, DCM/MeOH). Yield 25.2 mg (75%), pale yellow oil. IR (ATR): 2908s, 2890s, 2807m, 2769w, 2681w, 1605m, 1578s, 1564m, 1497m, 1454m, 1423s, 1382s, 1359m, 1334m, 1324w, 1300m, 1287w, 1200w, 1189w, 1166w, 1152w, 1130m, 1098w, 1069w, 1025w, 1011m, 953w, 940w, 928w, 913w, 882w, 872s, 819s, 773w. ^{1}H NMR (400 MHz, mixture of CD_3OD and $CDCl_3$, δ): 8.67 (d, J = 5.0, 1H, H-C(2)), 8.0 (d, J = 2.2, 1H, H-C(8)), 7.92 (d, J = 9.0, 1H, H-C(5)), 7.38 (dd, J_1 = 9.0, J_2 = 2.2, 1H, H-C(6)), 6.79 (d, J = 5.0, 1H, H-C(3)), 3.22 – 3.14 (m, 4H, Heq-piperazine), 2.79 – 2.70 (m, 4H, Hax-piperazine), 2.06 (s, 2H, H-C(11)), 1.99 – 1.90 (m, 3H, 3xC*H*(Ad)), 1.75 – 1.56 (m, 6H, 3xCH2(Ad)), 1.54 – 1.45 (m, 6H, 3xCH2(Ad)). ^{13}C NMR (101 MHz, $CDCl_3$, δ): 157.27 (C4), 151.86 (C2), 150.12 (C8a), 134.76 (C7), 128.75 (C8), 125.91 (C6), 125.36 (C5), 121.98 (C4a), 108.86 (C3), 71,40 (C11), 56.28 (C10 and C10'), 53.03 (C9 and C9'), 41.0 (3xCH2(Ad)), 37.23 (3xCH2(Ad)), 35.10 (C(Ad)), 28.48 (3xCH(Ad)). (+)TOF-HRMS (*m/z*): calc. for $[C_{24}H_{30}ClN_3 + H]^+$ 396.2207, found 396.2201. HPLC purity: Method I: RT 9.948, area 97.30 %; Method II: RT 8.994, area 95.55 %.

Scheme S6

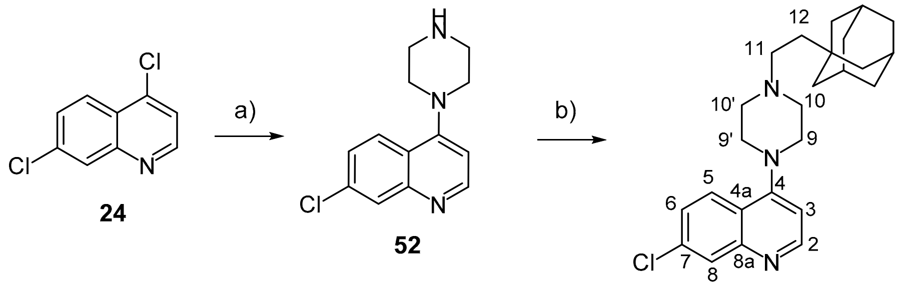

24 **52** **21 (KBK22)**

a) piperazine, neat Et_3N, 1h at 80 °C, than 6h at 130 °C; b) **35**, $NaBH(OAc)_3$, DCM, r.t.

4-{4-[2-(1-adamantyl)ethyl]piperazin-1-yl}-7-chloroquinoline (52) [78]

Solution of **24** (100 mg, 0.50 mmol), piperazine (130.5 mg, 1.52 mmol) and Et_3N (70 µL, 0.50 mmol) was stirred 1h at 80 °C, then 6h at 130 °C. The mixture was cooled to room temperature, dissolved in DCM, washed with water solution of $NaHCO_3$ (5%, 2 x 5mL), once with brine and dried over anhydrous Na_2SO_4. The product **52** was isolated after column chromatography (dry-flash, DCM/MeOH). Yield: 77.5 mg (62%, pale yellow oil. IR (ATR): 3252s, 3068w, 2996w, 2942m, 2880m, 2830s, 2804m, 2749m, 2639w, 1980w, 1667w, 1606m, 1569s, 1495m, 1456m, 1423s, 1375s,

1326w, 1292m, 1246m, 1228m, 1192w, 1147m, 1129m, 1106m, 1075m, 1019m, 944w, 913w, 869s, 840s, 820s, 771w, 712w, 647w, 565w, 514w, 499w, 451w. ^{1}H NMR (500 MHz, CD_3OD, δ): 8.66 (d, *J* = 5.2, 1H, H-C(2)), 8.08 (d, *J* = 9.0, 1H, H-C(5)), 7.94 (d, *J* = 2.1, 1H, H-C(8)), 7.53 (dd, J_1 = 9.1, J_2 = 2.1, 1H, H-C(6)), 7.02 (d, *J* = 5.2, 1H, H-C(3)), 3.29 – 3.21 (m, 4H, Heq-piperazine), 3.17 – 3.09 (m, 4H, Hax-piperazine). ^{13}C NMR (126 MHz, CD_3OD, δ): 158.35 (C4), 151.73 (C2), 149.44 (C8a), 135.32 (C7), 127.15 (C8), 126.17 (C6), 125.96 (5), 121.97 (C4a), 109.12(C3), 52.83 (C9 and C9'), 46.21 (C10 and C10').

4-{4-[2-(1-adamantyl)ethyl]piperazin-1-yl}-7-chloroquinoline (21)

Applying the general procedure for reductive amination, derivative **21** was obtained from amine **KBK19** (75.0 mg, 0.30 mmol) and aldehyde **N° 2** (54.0 mg, 0.30 mmol), using $NaHB(OAc)_3$ (128.4 mg, 0.6 mmol). Yield: 43.4 mg (35 %) pale yellow oil. IR (ATR): 3369w, 3034w, 2902s, 2844s, 2681w, 1606m, 1575s, 1498m, 1451s, 1425s, 1379s, 1299s, 1252m, 1192w, 1135s, 1071w, 1007m, 928w, 874s, 825s, 736m, 630w. ^{1}H NMR (500 MHz, $CDCl_3$, δ): 8.70 (d, *J* = 5.1, 1H, H-C(2)), 8.02 (d, *J* = 2.1, 1H, H-C(8)), 7.92 (d, *J* = 9.0, 1H, H-C(5)), 7.39 (dd, J_1 = 8.8, J_2 = 2.1, 1H, H-C(6)), 6.81 (d, *J* = 5.0, 1H, H-C(3)), 3.30 – 3.17 (m, 4H, Heq-piperazine), 2.78 – 2.65 (m, 4H, Hax-piperazine), 2.49 – 2.42 (m, 2H, H-C(11), 1.97 – 1.91 (m, 3H, 3xC*H*(Ad)), 1.74 – 1.58 (m, 6H, $3xCH_2(Ad)$), 1.56 – 1.46 (d, *J* = 2.9, 6H, $3xCH_2(Ad)$), 1.37 – 1.29 (m, 2H, H-C(12)). ^{13}C NMR (126 MHz, $CDCl_3$, δ): 157.07 (C4), 151.96 (C2), 150.07 (C8a), 134.88 (C7), 128.78 (C8), 126.05 (C6), 125.24 (C5), 121.90 (C4a), 108.90 (C3), 53.25 (C9) and C(9'), 52.82 (C11), 52.19 (C10) and (C10'), 42.58 ($3xCH_2(Ad)$), 41.01 (C12), 37.14 ($3xCH_2(Ad)$), 31.79 (C(Ad)), 28.66 (3xCH(Ad)). (+)TOF-HRMS (*m/z*): calc. for $[C_{24}H_{30}N_3Cl + H]^+$ 396.2207, found 396.2216. HPLC purity: Method I: RT 6.944, area 98.01 %; Method II: RT 8.980, area 97.38 %.

References

1. Terzić, N., Konstantinović, J., Tot, M., Burojević, J., Djurković-Djaković, O., Srbljanović, J., Štajner, T., Verbić, T., Zlatović, M., Machado, M., Albuquerque, I. S., Prudêncio, M., Sciotti, R. J., Pecic, S., D'Alessandro, S., Taramelli, D., Šolaja, B. A. Reinvestigating old pharmacophores: are 4-aminoquinolines and tetraoxanes potential two-stage antimalarials?. *J Med Chem* **2016**, *59*, 264−281.

2. Konstantinović, J., Videnović, M., Orsini, S., Bogojević, K., D'Alessandro, S., Scaccabarozzi, D., Terzić Jovanović, N., Gradoni, L., Basilico, N., Šolaja, B. A. Novel aminoquinoline deriva-tives significantly reduce parasite load in Leish-mania infantum infected mice. *ACS Med Chem Lett* **2018**, *9*, 629−634.
