# Supplementary material for: 4-Aminoquinoline-Based Adamantanes as Potential Anticholinesterase Agents in Symptomatic Treatment of Alzheimer’s Disease
Source: Pharmaceutics. 2022 Jun 20;14(6):1305. doi: 10.3390/pharmaceutics14061305 (PMC9229919; doi:10.3390/pharmaceutics14061305)
Supplement: Supplementary file 1 [file pharmaceutics-14-01305-s001.zip › Supplementary Materials File S2_final_Proof2_Opsenica.pdf]

Supplementary Information 2

4-aminoquinolines-based adamantanes as potential anticholinesterase agents in symptomatic treatment of Alzheimer's disease

Katarina Komatović[1], Ana Matošević[2], Suzana Žunec[2], Nataša Terzić-Jovanović[3], Sandra Šegan[3], Mario Zlatović,[1] Nikola Maraković[2], Anita Bosak[2,*], Dejan Opsenica[3,4,*]

[1] University of Belgrade - Faculty of Chemistry, Studentski trg 12 -16, 11158 Belgrade, Serbia,

[2] Institute for Medical Research and Occupational Health, Ksaverska cesta 2, Zagreb, Croatia,

[3] University of Belgrade – Institute of Chemistry, Technology and Metallurgy (IHTM), Njegoševa 12, 11000 Belgrade, Serbia,

[4] Centre of Excellence in Environmental Chemistry and Engineering, ICTM, 11000 Belgrade, Serbia

* Correspondence: abosak@imi.hr (A.B.); dopsen@chem.bg.ac.rs (D.O.)

Content:

**Detailed description of the docking procedure**	S2
**List of interactions between tested aminoquinolines and AChE**	S4
**List of interactions between tested aminoquinolines and BChE**	S13
**Prediction of drug-likeness and blood-brain barrier (BBB) penetration**	S21
**Table S1** *In silico* determined physicochemical characteristics of the tested aminoquinolines important for compounds to be orally active in humans and their *in silico* determined ability to pass the brain-blood barrier by passive transport.	S21
***In vitro* determined the antioxidative potential of 4-aminoquinolines**	S22
**Table S2** Determined FRAP values of tested aminoquinolines	S22
**Chromatographic determination of lipophilicity**	S23
**Multivariate statistical analysis and modelling**	S23
**Principal Component Analysis**	S24
**Figure S1.** PC1–PC2 score plots of retention parameters and descriptors (A and C respectively) and factor loadings (B and D respectively).	S26
**Quantitative Structure-Property Relationship (QSPR)**	S26
**Table S3.** The statistical performances of QSPR models connecting the most contributing molecular descriptors with molecular lipophilicity	S28
**References**	S29

Detailed description of docking procedure

Ligands to be docked in the enzyme structures were created with ChemBio3D Ultra 13.0 (PerkinElmer, Inc., Waltham, MA, USA) and minimized using the CHARMm force field and Smart Minimizer minimization method of Minimize Ligands protocol implemented in Biovia Discovery Studio Client v18.1. (Dassault Systèmes, Vélizy-Villacoublay, France). Before starting molecular docking protocol ligands were prepared to utilize Prepare Ligands protocol with regards to possible different protonation states, isomers and tautomers at pH 7.4. Flexible Docking protocol [1] was used for docking ligands into the enzyme receptors with flexibly selected residues. The flexible protocol consisted of the following steps: enzyme conformations calculation with ChiFlex, ligand conformations creation, ligand docking into active enzyme conformations with LibDock, pose clustering to remove similar ligand poses, enzyme conformations rebuilding by refining selected enzyme side-chains in the presence of the rigid ligand with ChiRotor, and final ligand refinement using CDOCKER.

The enzyme? structures were prepared starting from the crystal structures of free AChE (PDB ID: 4EY4) [2] and BChE (PDB ID: 1P0I) [3]. Next, a network of conserved water molecules identified through superposition of numerous water networks from X-ray structures of various AChE/BChE complexes available from PDB database was mapped onto AChE/BChE structures. The binding site within AChE and BChE model receptors was defined by a sphere (r = 13.5 Å and 14.0 Å, respectively) surrounding the residues that outline the active sites gorge. Following residues were selected as flexible: Tyr72, Trp86, Tyr124, Tyr133, Ser203, Trp236, Trp286, Phe297, Phe297, Glu334, Tyr337, Phe338, Tyr341, His447, and Tyr449 in case of AChE and Asn68, Asp70, Trp82, Gln119, Tyr128, Glu198, Ser198, Pro285, Leu286, Ser287, Trp231, Glu325, Phe329, Phe398, and His438 in case of BChE [4–6]. The rest of the parameters from the Flexible Docking protocol were set as follows: A maximum number of enzyme conformations processed for ligand docking was set to 100; Minimum angel, i.e. torsion angle cut off (degrees) to determine if two side-chain chi1 angles are the same or not, was set to 15; Confirmation Method, i.e. algorithm for generating ligand conformations, was set to BEST; a maximum number of conformations to be generated was set to 255; Energy Threshold, i.e. relative energy threshold (kcal/mol) within which conformations of separate isomers are created, was set to 20. Parameters for LibDock ligand docking were set as follows: Number of Hotspots, i.e. number of polar or apolar receptor hotspots for conformer matching, and Max Hits to Save were set to 100 and 20, respectively. Tolerance for a hotspot matching algorithm to dock ligands was set to 0.25; Maximum Number of Hits which specifies the maximum number of hits saved for each ligand during hotspots matching before the final pose minimization was set to 100 with Final Score Cutoff, i.e. fraction of the top-scoring poses reported set to 0.5; a maximum number of poses to be kept per conformation (i.e. Max Conformation Hits) was set to 30 with Maximum number of conformations

for each ligand (i.e. Max Start Conformations) set to 1000; Steric Fraction defining number of clashes before the pose-hotspot alignment is terminated was set to 0.10 within the 0.5 Å Final Cluster Radius; maximum values for the Apolar SASA Cutoff and Polar SASA Cutoff values were set to 15.0 Å and 5.0 Å, respectively. Finally, simulated annealing refinement was performed with 2000 heating phase steps during simulated annealing, 5000 steps of the cooling phase, and Heating Target Temperature and Cooling Target Temperature set to 700 K and 310 K, respectively.

The representative pose of each of the docked ligands was chosen based on the highest score as predicted by the scoring functions estimating binding affinities, implemented in the Biovia Discovery Studio Client v18.1. Score Ligand Poses protocol. The following scoring functions were calculated: CDOCKER Scores, LigScore1, LigScore2, PLP1, PLP2, Jain, PMF, PMF04, Ludi Energy Estimate 1, Ludi Energy Estimate 2, and Ludi Energy Estimate 3. To identify the poses of docked ligands that score high in more than one scoring function, the Biovia Discovery Studio Client v18.1 Consensus Score protocol was used. The Consensus Score protocol calculates the consensus scores of a series of docked ligands for which other scores have been previously computed. For each selected scoring function, the ligands are listed by score in descending order. The consensus score for a ligand is an integer between zero (none of the scores are in the top-ranking percentile) and the total number of scores (all of the scores are in the top-ranking percentile) listed in Input Properties. Thus, in the Parameters Explorer of Consensus Score protocol, following scoring functions were chosen to calculate the consensus score: -CDOCKER_ENERGY, -CDOCKER_INTERACTION_ENERGY, LigScore1, LigScore2, PLP1, PLP2, Jain, PMF, PMF04, Ludi Energy Estimate 1, Ludi Energy Estimate 2, and Ludi Energy Estimate 3. Consensus Percentage was set to 40 to specify the percentage of top molecules to include in the consensus. Use Best Pose only was set to False. Finally, the poses with highest consensus score were chosen as the representative poses of each of the docked ligands and their model complexes ranked by CDOCKER energy as a measure of binding energy.

List of interactions between aminoquinolines and AChE.

Interactions between compound **1** and AChE.

Amino acid	Non-bonding interactions
Tyr124	H52, hydrogen bond-conventional hydrogen bond
Tyr124	H49, hydrogen bond-carbon hydrogen bond
Tyr341	N25, electrostatic-π-cation
Trp86	**1**, hydrophobic-π-π T-shaped
Trp86	**1**, hydrophobic-π-π T-shaped
Phe338	**1**, hydrophobic-π-π T-shaped
His447	**1**, hydrophobic-π-π T-shaped
His447	**1**, hydrophobic-π-π T-shaped
Tyr72	C7, hydrophobic-π-alkyl
Tyr72	**1**, hydrophobic-π-alkyl
Trp86	Cl21, hydrophobic-π-alkyl
Trp86	Cl21, hydrophobic-π-alkyl
Tyr124	**1**, hydrophobic-π-alkyl
Trp286	C7, hydrophobic-π-alkyl
Trp286	**1**, hydrophobic-π-alkyl

Interactions between compound **2** and AChE.

Amino acid	Non-bonding interactions
Glu202	N17, electrostatic-attractive charge
Asp74	N17, electrostatic-attractive charge
His447	H46, hydrogen bond-conventional hydrogen bond
Tyr124	H57, hydrogen bond-conventional hydrogen bond
His447	H47, hydrogen bond-carbon hydrogen bond
Trp86	N17, electrostatic-π-cation
Trp86	N17, electrostatic-π-cation
Tyr337	N17, electrostatic-π-cation
Tyr341	N26, electrostatic-π-cation
Trp86	**2**, hydrophobic-π-π stacked
Trp86	**2**, hydrophobic-π-π stacked
Trp86	**2**, hydrophobic-π-π stacked
Trp86	**2**, hydrophobic-π-π stacked
Tyr337	**2**, hydrophobic-π-π T-shaped
Ile451	Cl21, hydrophobic-alkyl
Trp86	Cl21, hydrophobic-π-alkyl
Tyr124	**2**, hydrophobic-π-alkyl
Tyr133	Cl21, hydrophobic-π-alkyl
Trp286	**2**, hydrophobic-π-alkyl

Interactions between compound **3** and AChE.

Amino acid	Non-bonding interactions
Glu202	N17, electrostatic-attractive charge
His447	H47, hydrogen bond-conventional hydrogen bond
Tyr124	H56, hydrogen bond-conventional hydrogen bond
His447	H48, hydrogen bond-carbon hydrogen bond
Trp86	N17, electrostatic-π-cation
Trp86	N17, electrostatic-π-cation
Tyr337	N17, electrostatic-π-cation

Tyr341	N25, electrostatic-π-cation
Trp86	**3**, hydrophobic-π-π stacked
Trp86	**3**, hydrophobic-π-π stacked
Trp86	**3**, hydrophobic-π-π stacked
Trp86	**3**, hydrophobic-π-π stacked
Phe338	**3**, hydrophobic-π-π stacked
Ile451	Cl21, hydrophobic-alkyl
Tyr72	**3**, hydrophobic-π-alkyl
Trp86	Cl21, hydrophobic-π-alkyl
Tyr124	C7, hydrophobic-π-alkyl
Tyr124	**3**, hydrophobic-π-alkyl
Tyr133	Cl21, hydrophobic-π-alkyl

Interactions between compound **4** and AChE.

Amino acid	Non-bonding interactions
HOH708	H35, hydrogen bond-water hydrogen bond; carbon hydrogen bond
Ser203	H65, hydrogen bond-conventional hydrogen bond
His447	N29, electrostatic-π-cation
Trp86	H54, hydrophobic-π-sigma
His447	H68, hydrophobic-π-sigma
Tyr72	**4**, hydrophobic-π-π T-shaped
Tyr124	**4**, hydrophobic-π-π T-shaped
Leu289	Cl11, hydrophobic-alkyl
Trp86	C25, hydrophobic-π-alkyl
Trp86	**4**, hydrophobic-π-alkyl
Trp86	**4**, hydrophobic-π-alkyl
Trp86	C25, hydrophobic-π-alkyl
Trp86	**4**, hydrophobic-π-alkyl
Trp286	Cl11, hydrophobic-π-alkyl
Tyr337	**4**, hydrophobic-π-alkyl

Interactions between compound **5** and AChE.

Amino acid	Non-bonding interactions
Tyr124	H71, hydrogen bond-conventional hydrogen bond
Asp74	H45, hydrogen bond-carbon hydrogen bond
Tyr72	N7, electrostatic-π-cation
Trp286	N7, electrostatic-π-cation
Trp286	N7, electrostatic-π-cation
Trp286	**5**, hydrophobic-π-π stacked
Trp286	**5**, hydrophobic-π-π stacked
Trp286	**5**, hydrophobic-π-π stacked
Trp286	**5**, hydrophobic-π-π stacked
Tyr72	Cl11, hydrophobic-π-alkyl
Trp86	**5**, hydrophobic-π-alkyl
Trp86	C27, hydrophobic-π-alkyl
Trp86	**5**, hydrophobic-π-alkyl
Trp86	C27, hydrophobic-π-alkyl
Tyr124	Cl11, hydrophobic-π-alkyl
Tyr337	**5**, hydrophobic-π-alkyl
Phe338	**5**, hydrophobic-π-alkyl
His447	C27, hydrophobic-π-alkyl

His447	**5**, hydrophobic-π-alkyl

Interactions between compound **6** and AChE.

Amino acid	Non-bonding interactions
HOH752	F2, hydrogen bond; halogen-water hydrogen bond; conventional hydrogen bond; halogen (flourine)
HOH772	F2, hydrogen bond; halogen-water hydrogen bond; conventional hydrogen bond; halogen (flourine)
HOH809	H56, hydrogen bond-water hydrogen bond; conventional hydrogen bond
Ser203	H48, hydrogen bond-conventional hydrogen bond
Tyr124	H57, hydrogen bond-conventional hydrogen bond
Gly120	F4, halogen-halogen (fluorine)
Glu202	F3, halogen-halogen (fluorine)
Glu202	F4, halogen-halogen (fluorine)
Tyr341	N28, electrostatic-π-cation
Tyr124	H33, hydrophobic-π-sigma
Trp86	**6**, hydrophobic-π-π T-shaped
Trp86	**6**, hydrophobic-π-π T-shaped
Trp86	C1, hydrophobic-π-alkyl
Trp86	C1, hydrophobic-π-alkyl

Interactions between compound **7** and AChE.

Amino acid	Non-bonding interactions
Glu202	N21, electrostatic-attractive charge
Asp74	N29, electrostatic-attractive charge
Gly122	F1, hydrogen bond; halogen-conventional hydrogen bond; halogen (flourine)
Ala204	F1, hydrogen bond; halogen-conventional hydrogen bond; halogen (flourine)
Asp74	H59, hydrogen bond-conventional hydrogen bond
Asp74	H60, hydrogen bond-conventional hydrogen bond
Gly122	F1, hydrogen bond; halogen-carbon hydrogen bond; halogen (flourine)
Ser203	F1, hydrogen bond-carbon hydrogen bond
Tyr124	H54, hydrogen bond-carbon hydrogen bond
Tyr124	H58, hydrogen bond-carbon hydrogen bond
His447	F3, halogen-halogen (fluorine)
His447	N21, electrostatic-π-cation
Tyr341	N29, electrostatic-π-cation
Trp86	**7**, hydrophobic-π-π T-shaped
Trp86	**7**, hydrophobic-π-π T-shaped
Phe338	**7**, hydrophobic-π-π T-shaped
Phe338	**7**, hydrophobic-π-π T-shaped
His447	**7**, hydrophobic-π-π T-shaped
His447	**7**, hydrophobic-π-π T-shaped
Trp286	Cl1, hydrophobic-π-alkyl
Trp286	**7**, hydrophobic-π-alkyl
Trp286	Cl1, hydrophobic-π-alkyl
Trp286	**7**, hydrophobic-π-alkyl
Phe295	C2, hydrophobic-π-alkyl
Phe297	C2, hydrophobic-π-alkyl

Interactions between compound **8** and AChE.

Amino acid	Non-bonding interactions
Glu202	N15, electrostatic-attractive charge
HOH715	H32, hydrogen bond-water hydrogen bond; carbon hydrogen bond
Tyr124	H39, hydrogen bond-conventional hydrogen bond
Asp74	H37, hydrogen bond-carbon hydrogen bond
Asp74	H41, hydrogen bond-carbon hydrogen bond
Tyr341	N15, electrostatic-π-cation
Trp286	**8**, hydrophobic-π-π stacked
Trp286	**8**, hydrophobic-π-π stacked
Trp286	**8**, hydrophobic-π-π stacked
Trp286	**8**, hydrophobic-π-π stacked
Trp86	**8**, hydrophobic-π-alkyl
Tyr124	C24, hydrophobic-π-alkyl
Trp286	Cl11, hydrophobic-π-alkyl
Tyr337	**8**, hydrophobic-π-alkyl
Tyr337	**8**, hydrophobic-π-alkyl
Phe338	**8**, hydrophobic-π-alkyl
His447	**8**, hydrophobic-π-alkyl

Interactions between compound **9** and AChE.

Amino acid	Non-bonding interactions
Tyr341	H43, hydrogen bond-conventional hydrogen bond
Asp74	**9**, electrostatic-π-anion
Trp286	**9**, hydrophobic-π-π stacked
Trp286	**9**, hydrophobic-π-π stacked
Trp286	**9**, hydrophobic-π-π stacked
Trp286	**9**, hydrophobic-π-π stacked
Tyr124	Cl11, hydrophobic-π-alkyl
Tyr341	Cl11, hydrophobic-π-alkyl

Interactions between compound **10** and AChE.

Amino acid	Non-bonding interactions
HOH784	**10**, hydrogen bond-water hydrogen bond; π-donor hydrogen bond
HOH787	H46, hydrogen bond-water hydrogen bond; carbon hydrogen bond
Trp86	N7, electrostatic-π-cation
Trp286	N15, electrostatic-π-cation
Trp86	**10**, hydrophobic-π-π T-shaped
Tyr337	**10**, hydrophobic-π-π T-shaped
Leu76	**10**, hydrophobic-alkyl
Tyr72	C34, hydrophobic-π-alkyl
Tyr72	**10**, hydrophobic-π-alkyl
Trp286	**10**, hydrophobic-π-alkyl
His287	**10**, hydrophobic-π-alkyl
His447	Cl11, hydrophobic-π-alkyl

Interactions between compound **11** and AChE.

Amino acid	Non-bonding interactions
Trp286	H60, hydrogen bond; electrostatic-π-cation; π-donor hydrogen bond
Trp286	H60, hydrogen bond; electrostatic-π-cation; π-donor hydrogen bond

Trp86	**11**, hydrophobic-π-π T-shaped
Trp86	**11**, hydrophobic-π-π T-shaped
Phe338	**11**, hydrophobic-π-π T-shaped
Tyr72	**11**, hydrophobic-π-alkyl
Trp86	Cl11, hydrophobic-π-alkyl
Trp86	Cl11, hydrophobic-π-alkyl
His287	C26, hydrophobic-π-alkyl
His447	Cl11, hydrophobic-π-alkyl

Interactions between compound **12** and AChE.

Amino acid	Non-bonding interactions
Asp74	N25, electrostatic-attractive charge
HOH809	H54, hydrogen bond-water hydrogen bond; conventional hydrogen bond
His447	H47, hydrogen bond-conventional hydrogen bond
Tyr124	H53, hydrogen bond-conventional hydrogen bond
His447	H48, hydrogen bond-carbon hydrogen bond
Tyr124	H50, hydrogen bond-carbon hydrogen bond
Tyr341	N25, electrostatic-π-cation
Trp86	**12**, hydrophobic-π-π stacked
Trp86	**12**, hydrophobic-π-π stacked
Trp86	**12**, hydrophobic-π-π stacked
Trp86	**12**, hydrophobic-π-π stacked
Phe338	**12**, hydrophobic-π-π stacked
His447	**12**, hydrophobic-π-π T-shaped
Ile451	Cl21, hydrophobic-alkyl
Tyr72	C7, hydrophobic-π-alkyl
Tyr72	**12**, hydrophobic-π-alkyl
Trp86	Cl21, hydrophobic-π-alkyl
Tyr124	**12**, hydrophobic-π-alkyl
Tyr133	Cl21, hydrophobic-π-alkyl
Trp286	C7, hydrophobic-π-alkyl
Trp286	**12**, hydrophobic-π-alkyl
Tyr337	C27, hydrophobic-π-alkyl
Tyr341	C27, hydrophobic-π-alkyl

Interactions between compound **13** and AChE.

Amino acid	Non-bonding interactions
Asp74	H50, hydrogen bond; electrostatic-salt bridge; attractive charge
Glu202	N28, electrostatic-attractive charge
HOH869	H50, hydrogen bond-water hydrogen bond; conventional hydrogen bond
Ser203	H57, hydrogen bond-conventional hydrogen bond
Tyr341	N21, electrostatic-π-cation
Trp286	**13**, hydrophobic-π-π stacked
Tyr341	**13**, hydrophobic-π-π stacked
Tyr341	**13**, hydrophobic-π-π stacked
Tyr124	**13**, hydrophobic-π-π T-shaped
Tyr124	**13**, hydrophobic-π-π T-shaped
Trp86	**13**, hydrophobic-π-alkyl
Trp86	**13**, hydrophobic-π-alkyl
Trp86	**13**, hydrophobic-π-alkyl
Trp86	**13**, hydrophobic-π-alkyl

Trp286	C1, hydrophobic-π-alkyl
Trp286	C1, hydrophobic-π-alkyl
Phe295	C30, hydrophobic-π-alkyl
Phe297	C30, hydrophobic-π-alkyl
Tyr337	C11, hydrophobic-π-alkyl
Tyr337	**13**, hydrophobic-π-alkyl
Phe338	C30, hydrophobic-π-alkyl
Tyr341	C1, hydrophobic-π-alkyl
His447	C11, hydrophobic-π-alkyl

Interactions between compound **14** and AChE.

Amino acid	Non-bonding interactions
Asp74	N25, electrostatic-attractive charge
HOH809	H61, hydrogen bond-water hydrogen bond; carbon hydrogen bond
Glu202	H48, hydrogen bond-conventional hydrogen bond
Tyr124	H51, hydrogen bond-carbon hydrogen bond
Asp74	H57, hydrogen bond-carbon hydrogen bond
Tyr124	H62, hydrogen bond-carbon hydrogen bond
Tyr341	N25, electrostatic-π-cation
Trp86	**14**, hydrogen bond-π-donor hydrogen bond
Trp86	**14**, hydrophobic-π-π stacked
Trp86	**14**, hydrophobic-π-π stacked
Tyr337	**14**, hydrophobic-π-π T-shaped
Met85	Cl21, hydrophobic-alkyl
Trp86	C21, hydrophobic-π-alkyl
Trp86	C21, hydrophobic-π-alkyl
Tyr124	**14**, hydrophobic-π-alkyl
Trp286	C7, hydrophobic-π-alkyl
Phe297	C27, hydrophobic-π-alkyl
Phe338	C27, hydrophobic-π-alkyl
Tyr449	Cl21, hydrophobic-π-alkyl

Interactions between compound **15** and AChE.

Amino acid	Non-bonding interactions
Asp74	H55, hydrogen bond; electrostatic-salt bridge; attractive charge
Glu202	N17, electrostatic-attractive charge
His447	H48, hydrogen bond-conventional hydrogen bond
His447	H49, hydrogen bond-carbon hydrogen bond
Trp86	N17, electrostatic-π-cation
Trp86	N17, electrostatic-π-cation
Tyr337	N17, electrostatic-π-cation
Tyr341	N24, electrostatic-π-cation
Trp86	**15**, hydrophobic-π-π stacked
Trp86	**15**, hydrophobic-π-π stacked
Trp86	**15**, hydrophobic-π-π stacked
Trp86	**15**, hydrophobic-π-π stacked
Tyr337	**15**, hydrophobic-π-π T-shaped
Tyr124	**15**, hydrophobic-π-alkyl
Trp286	C7, hydrophobic-π-alkyl

Interactions between compound **16** and AChE.

Amino acid	Non-bonding interactions
Glu202	N17, electrostatic-attractive charge
HOH809	H62, hydrogen bond-water hydrogen bond; carbon hydrogen bond
His447	H49, hydrogen bond-conventional hydrogen bond
His447	H50, hydrogen bond-carbon hydrogen bond
Tyr124	H64, hydrogen bond-carbon hydrogen bond
Trp86	N17, electrostatic-π-cation
Trp86	N17, electrostatic-π-cation
Tyr337	N17, electrostatic-π-cation
Trp86	**16**, hydrophobic-π-π stacked
Trp86	**16**, hydrophobic-π-π stacked
Trp86	**16**, hydrophobic-π-π stacked
Trp86	**16**, hydrophobic-π-π stacked
Tyr337	**16**, hydrophobic-π-π T-shaped
His447	**16**, hydrophobic-π-π T-shaped
Tyr72	**16**, hydrophobic-π-alkyl
Trp286	**16**, hydrophobic-π-alkyl

Interactions between compound **17** and AChE.

Amino acid	Non-bonding interactions
Trp286	N17, electrostatic-π-cation
Trp286	N17, electrostatic-π-cation
His447	H45, hydrophobic-π-sigma
Tyr124	KBK15, other-π-lone pair
Trp286	**17**, hydrophobic-π-π stacked
Trp286	**17**, hydrophobic-π-π stacked
Trp286	**17**, hydrophobic-π-π stacked
Trp286	**17**, hydrophobic-π-π stacked
Tyr124	**17**, hydrophobic-π-π T-shaped
Trp86	**17**, hydrophobic-π-alkyl
Trp86	C7, hydrophobic-π-alkyl
Trp86	**17**, hydrophobic-π-alkyl
Trp86	**17**, hydrophobic-π-alkyl
Trp86	C7, hydrophobic-π-alkyl
Trp86	**17**, hydrophobic-π-alkyl
Tyr337	**17**, hydrophobic-π-alkyl
His447	**17**, hydrophobic-π-alkyl

Interactions between compound **18** and AChE.

Amino acid	Non-bonding interactions
Asp74	N17, electrostatic-attractive charge
HOH708	H50, hydrogen bond-water hydrogen bond; carbon hydrogen bond
Trp286	**18**, hydrophobic-π-π stacked
Trp286	**18**, hydrophobic-π-π stacked
Trp286	**18**, hydrophobic-π-π stacked
Trp286	**18**, hydrophobic-π-π stacked
Tyr341	**18**, hydrophobic-π-π stacked
Tyr124	**18**, hydrophobic-π-π T-shaped
Trp86	**18**, hydrophobic-π-alkyl
Trp86	C7, hydrophobic-π-alkyl
Trp86	**18**, hydrophobic-π-alkyl

Trp86	**18**, hydrophobic-π-alkyl
Trp86	C7, hydrophobic-π-alkyl
Trp86	**18**, hydrophobic-π-alkyl
Trp286	Cl22, hydrophobic-π-alkyl
Tyr337	**18**, hydrophobic-π-alkyl
His447	C7, hydrophobic-π-alkyl

Interactions between compound **19** and AChE.

Amino acid	Non-bonding interactions
Glu202	N17, electrostatic-attractive charge
His447	H54, hydrogen bond-conventional hydrogen bond
Asp74	H61, hydrogen bond-conventional hydrogen bond
His447	H54, hydrogen bond-carbon hydrogen bond
Trp86	N17, electrostatic-π-cation
Trp86	N17, electrostatic-π-cation
Tyr124	**19**, other-π-lone pair
Trp86	**19**, hydrophobic-π-π stacked
Trp86	**19**, hydrophobic-π-π stacked
Trp86	**19**, hydrophobic-π-π stacked
Trp86	**19**, hydrophobic-π-π stacked
Tyr124	**19**, hydrophobic-π-π T-shaped
His447	**19**, hydrophobic-π-π T-shaped
Trp86	Cl22, hydrophobic-π-alkyl
Tyr133	Cl22, hydrophobic-π-alkyl
Trp286	C7, hydrophobic-π-alkyl
Trp286	**19**, hydrophobic-π-alkyl
Trp286	**19**, hydrophobic-π-alkyl

Interactions between compound **20** and AChE.

Amino acid	Non-bonding interactions
Glu202	N1, electrostatic-attractive charge
HOH809	H39, hydrogen bond-water hydrogen bond; carbon hydrogen bond
His447	H29, hydrogen bond-conventional hydrogen bond
Ap74	H37, hydrogen bond-carbon hydrogen bond
Tyr124	H42, hydrogen bond-carbon hydrogen bond
Trp86	N1, electrostatic-π-cation
Trp86	N1, electrostatic-π-cation
Tyr337	N1, electrostatic-π-cation
Trp86	**20**, hydrophobic-π-π stacked
Trp86	**20**, hydrophobic-π-π stacked
Trp86	**20**, hydrophobic-π-π stacked
Trp86	**20**, hydrophobic-π-π stacked
Phe338	**20**, hydrophobic-π-π stacked
Tyr72	C26, hydrophobic-π-alkyl
Trp86	Cl28, hydrophobic-π-alkyl
Tyr124	**20**, hydrophobic-π-alkyl
Tyr341	**20**, hydrophobic-π-alkyl

Interactions between compound **21** and AChE.

Amino acid	Non-bonding interactions
Glu202	N17, electrostatic-attractive charge

Tyr124	N25, hydrogen bond-conventional hydrogen bond
His447	H48, hydrogen bond-conventional hydrogen bond
His447	H49, hydrogen bond-carbon hydrogen bond
Trp86	N17, electrostatic-π-cation
Trp86	N17, electrostatic-π-cation
Tyr337	N17, electrostatic-π-cation
Trp86	**21**, hydrophobic-π-π stacked
Trp86	**21**, hydrophobic-π-π stacked
Trp86	**21**, hydrophobic-π-π stacked
Trp86	**21**, hydrophobic-π-π stacked
Phe338	**21**, hydrophobic-π-π stacked
Tyr337	**21**, hydrophobic-π-π T-shaped
His447	**21**, hydrophobic-π-π T-shaped
Tyr72	**21**, hydrophobic-π-alkyl
Trp86	Cl22, hydrophobic-π-alkyl
Trp286	**21**, hydrophobic-π-alkyl
Tyr337	**21**, hydrophobic-π-alkyl

Interactions between compound **22** and AChE.

Amino acid	Non-bonding interactions
HOH883	H55, hydrogen bond-water hydrogen bond; conventional hydrogen bond
HOH883	H56, hydrogen bond-water hydrogen bond; carbon hydrogen bond
HOH708	H71, hydrogen bond-water hydrogen bond; carbon hydrogen bond
Tyr337	H79, hydrogen bond-conventional hydrogen bond
Tyr124	H80, hydrogen bond-conventional hydrogen bond
Tyr124	H77, hydrogen bond-carbon hydrogen bond
Tyr124	H80, hydrogen bond-carbon hydrogen bond
Trp286	**22**, hydrophobic-π-π stacked
Trp286	**22**, hydrophobic-π-π stacked
Trp286	**22**, hydrophobic-π-π stacked
Tyr72	**22**, hydrophobic-π-π T-shaped
Trp86	**22**, hydrophobic-π-alkyl
Trp86	**22**, hydrophobic-π-alkyl
Trp86	**22**, hydrophobic-π-alkyl
Trp86	C7, hydrophobic-π-alkyl
Trp86	**22**, hydrophobic-π-alkyl
Tyr124	Cl22, hydrophobic-π-alkyl
Phe297	Cl22, hydrophobic-π-alkyl
Tyr337	**22**, hydrophobic-π-alkyl
Tyr341	**22**, hydrophobic-π-alkyl
Phe297	**22**, hydrophobic-π-alkyl

Interactions between compound **23** and AChE.

Amino acid	Non-bonding interactions
Glu202	N17, electrostatic-attractive charge
HOH809	H64, hydrogen bond-water hydrogen bond; carbon hydrogen bond
HOH809	H70, hydrogen bond-water hydrogen bond; carbon hydrogen bond
Ser203	H56, hydrogen bond-conventional hydrogen bond
His447	H56, hydrogen bond-conventional hydrogen bond
Tyr124	H65, hydrogen bond-carbon hydrogen bond
Asp74	H66, hydrogen bond-carbon hydrogen bond

Tyr72	H83, hydrogen bond-carbon hydrogen bond
Tyr72	N35, electrostatic-π-cation
Trp286	H80, hydrogen bond; electrostatic-π-cation; π-donor hydrogen bond
Trp86	**23**, hydrophobic-π-π T-shaped
Trp86	**23**, hydrophobic-π-π T-shaped
Leu76	**23**, hydrophobic-alkyl
Trp86	Cl22, hydrophobic-π-alkyl
Trp86	Cl22, hydrophobic-π-alkyl
Tyr341	**23**, hydrophobic-π-alkyl

List of interactions between aminoquinolines and BChE.

Interactions between compound **1** and BChE.

Amino acid	Non-bonding interactions
Asp70	N25, electrostatic-attractive charge
HOH825	H53, hydrogen bond-water hydrogen bond; conventional hydrogen bond
Ser287	H45, hydrogen bond-conventional hydrogen bond
Ser287	H46, hydrogen bond-carbon hydrogen bond
Asp70	H51, hydrogen bond-carbon hydrogen bond
Trp82	C7, hydrophobic-π-alkyl
Trp82	**1**, hydrophobic-π-alkyl
Trp82	C7, hydrophobic-π-alkyl
Trp82	**1**, hydrophobic-π-alkyl
Trp231	Cl21, hydrophobic-π-alkyl
Trp231	Cl21, hydrophobic-π-alkyl
Phe398	Cl21, hydrophobic-π-alkyl
Leu286	**1**, hydrophobic-π-alkyl

Interactions between compound **2** and BChE.

Amino acid	Non-bonding interactions
Asp70	N26, electrostatic-attractive charge
HOH930	H50, hydrogen bond-water hydrogen bond; carbon hydrogen bond
HOH825	H57, hydrogen bond-water hydrogen bond; conventional hydrogen bond
Ser287	H46, hydrogen bond-conventional hydrogen bond
Trp82	H34, hydrophobic-π-sigma
Phe329	**2**, hydrophobic-π-π T-shaped
Leu286	Cl21, hydrophobic-alkyl
Val288	Cl21, hydrophobic-alkyl
Trp82	C7, hydrophobic-π-alkyl
Trp82	**2**, hydrophobic-π-alkyl
Trp82	**2**, hydrophobic-π-alkyl
Trp82	C7, hydrophobic-π-alkyl
Trp82	**2**, hydrophobic-π-alkyl
Trp231	Cl21, hydrophobic-π-alkyl
Trp231	Cl21, hydrophobic-π-alkyl
His438	**2**, hydrophobic-π-alkyl

Interactions between compound **3** and BChE.

Amino acid	Non-bonding interactions
HOH825	H56, hydrogen bond-water hydrogen bond; conventional hydrogen bond

HOH825	H61, hydrogen bond-water hydrogen bond; carbon hydrogen bond
Ser287	H47, hydrogen bond-conventional hydrogen bond
Trp82	N25, electrostatic-π-cation
Phe329	**3**, hydrophobic-π-π T-shaped
Leu286	Cl21, hydrophobic-alkyl
Val288	Cl21, hydrophobic-alkyl
Trp82	**3**, hydrophobic-π-alkyl
Trp82	**3**, hydrophobic-π-alkyl
Trp231	C21, hydrophobic-π-alkyl
Trp231	C21, hydrophobic-π-alkyl
His438	C7, hydrophobic-π-alkyl
Leu286	**3**, hydrophobic-π-alkyl

Interactions between compound **4** and BChE.

Amino acid	Non-bonding interactions
Asp70	N7, electrostatic-attractive charge
Glu197	N29, electrostatic-attractive charge
Gln119	H37, hydrogen bond-conventional hydrogen bond
Ser287	H37, hydrogen bond-conventional hydrogen bond
Ser198	H65, hydrogen bond-conventional hydrogen bond
Asp70	H35, hydrogen bond-carbon hydrogen bond
Pro285	H38, hydrogen bond-carbon hydrogen bond
Ser287	H38, hydrogen bond-carbon hydrogen bond
Pro285	H39, hydrogen bond-carbon hydrogen bond
Ser198	H42, hydrogen bond-carbon hydrogen bond
Trp82	H63, hydrophobic-π-sigma
Ala277	Cl11, hydrophobic-alkyl
Trp82	**4**, hydrophobic-π-alkyl
Trp82	C25, hydrophobic-π-alkyl
Trp82	**4**, hydrophobic-π-alkyl
Trp82	**4**, hydrophobic-π-alkyl
Trp82	C25, hydrophobic-π-alkyl
His438	**4**, hydrophobic-π-alkyl

Interactions between compound **5** and BChE.

Amino acid	Non-bonding interactions
HOH709	**5**, hydrogen bond-water hydrogen bond; π-donor hydrogen bond
His438	H39, hydrogen bond-conventional hydrogen bond
Gln119	H71, hydrogen bond-conventional hydrogen bond
His438	H41, hydrogen bond-carbon hydrogen bond
Pro285	H44, hydrogen bond-carbon hydrogen bond
Ser287	H45, hydrogen bond-carbon hydrogen bond
Gln119	H73, hydrogen bond-carbon hydrogen bond
Asp70	Cl11, halogen-halogen (Cl, Br, I)
Trp82	N7, electrostatic-π-cation
Trp82	H36, hydrogen bond; electrostatic-π-cation; π-donor hydrogen bond
Trp82	**5**, hydrophobic-π-π T-shaped
Trp82	**5**, hydrophobic-π-π T-shaped
Phe329	**5**, hydrophobic-π-π T-shaped
Tyr332	Cl11, hydrophobic-π-alkyl

Interactions between compound **7** and BChE.

Amino acid	Non-bonding interactions
Ser287	H49, hydrogen bond-conventional hydrogen bond
Gly117	F3, hydrogen bond; halogen-carbon hydrogen bond; halogen (fluorine)
Trp82	N29, electrostatic-π-cation
Trp82	H32, hydrophobic-π-sigma
Phe329	**7**, hydrophobic-π-π T-shaped
Leu286	C2, hydrophobic-alkyl
Val288	C2, hydrophobic-alkyl
Trp82	**7**, hydrophobic-π-alkyl
Trp82	**7**, hydrophobic-π-alkyl
Trp82	**7**, hydrophobic-π-alkyl
Trp231	C2, hydrophobic-π-alkyl
Trp231	C2, hydrophobic-π-alkyl
His438	**7**, hydrophobic-π-alkyl
Leu286	**7**, hydrophobic-π-alkyl

Interactions between compound **8** and BChE.

Amino acid	Non-bonding interactions
Asp70	N15, electrostatic-attractive charge
HOH930	H34, hydrogen bond-water hydrogen bond; carbon hydrogen bond
HOH910	H41, hydrogen bond-water hydrogen bond; carbon hydrogen bond
Ser287	H31, hydrogen bond-conventional hydrogen bond
Asp70	H39, hydrogen bond-conventional hydrogen bond
Asp70	H35, hydrogen bond-carbon hydrogen bond
Asp70	H37, hydrogen bond-carbon hydrogen bond
Phe329	DO280, hydrophobic-π-π T-shaped
Leu286	Cl11, hydrophobic-alkyl
Val288	Cl11, hydrophobic-alkyl
Trp82	C24, hydrophobic-π-alkyl
Trp82	**8**, hydrophobic-π-alkyl
Trp82	**8**, hydrophobic-π-alkyl
Trp82	C24, hydrophobic-π-alkyl
Trp82	**8**, hydrophobic-π-alkyl
Trp231	Cl11, hydrophobic-π-alkyl
Trp231	Cl11, hydrophobic-π-alkyl
His438	**8**, hydrophobic-π-alkyl

Interactions between compound **9** and BChE.

Amino acid	Non-bonding interactions
HOH1031	H46, hydrogen bond-water hydrogen bond; carbon hydrogen bond
Ser287	H45, hydrogen bond-conventional hydrogen bond
Ser287	H46, hydrogen bond-carbon hydrogen bond
Thr284	H49, hydrogen bond-carbon hydrogen bond
Phe329	**9**, hydrophobic-π-π T-shaped
Ala277	**9**, hydrophobic-alkyl
Pro285	**9**, hydrophobic-alkyl
Leu286	Cl11, hydrophobic-alkyl
Trp231	Cl11, hydrophobic-π-alkyl
Trp231	Cl11, hydrophobic-π-alkyl

Phe398	Cl11, hydrophobic-π-alkyl
Leu286	**9**, hydrophobic-π-alkyl

Interactions between compound **10** and BChE.

Amino acid	Non-bonding interactions
HOH897	H45, hydrogen bond-water hydrogen bond; conventional hydrogen bond
HOH930	H91, hydrogen bond-water hydrogen bond; carbon hydrogen bond
Gln119	H53, hydrogen bond-conventional hydrogen bond
Ser287	H54, hydrogen bond-carbon hydrogen bond
Pro285	H55, hydrogen bond-carbon hydrogen bond
Gln119	H90, hydrogen bond-carbon hydrogen bond
Trp231	H60, hydrophobic-π-sigma
Leu286	**10**, hydrophobic-alkyl
Val288	**10**, hydrophobic-alkyl
Leu286	**10**, hydrophobic-alkyl
Trp231	C23, hydrophobic-π-alkyl
Trp231	**10**, hydrophobic-π-alkyl
Trp231	**10**, hydrophobic-π-alkyl
Trp231	C23, hydrophobic-π-alkyl
Trp231	**10**, hydrophobic-π-alkyl
Phe329	**10**, hydrophobic-π-alkyl
Phe329	C34, hydrophobic-π-alkyl
His438	C34, hydrophobic-π-alkyl

Interactions between compound **11** and BChE.

Amino acid	Non-bonding interactions
Ser287	H50, hydrogen bond-carbon hydrogen bond
Ser287	H51, hydrogen bond-carbon hydrogen bond
Trp82	Cl11, hydrophobic-π-alkyl

Interactions between compound **12** and BChE.

Amino acid	Non-bonding interactions
Glu197	N25, electrostatic-attractive charge
His438	H53, hydrogen bond-conventional hydrogen bond
His438	H61, hydrogen bond-carbon hydrogen bond
Val288	C27, hydrophobic-alkyl
Trp82	C7, hydrophobic-π-alkyl
Trp82	**12**, hydrophobic-π-alkyl
Trp82	**12**, hydrophobic-π-alkyl
Trp82	C7, hydrophobic-π-alkyl
Trp82	**12**, hydrophobic-π-alkyl
Trp231	C27, hydrophobic-π-alkyl
Trp231	C27, hydrophobic-π-alkyl
His438	**12**, hydrophobic-π-alkyl

Interactions between compound **13** and BChE.

Amino acid	Non-bonding interactions
HOH825	H57, hydrogen bond-water hydrogen bond; conventional hydrogen bond
Ser287	H50, hydrogen bond-conventional hydrogen bond
Gly117	F2, hydrogen bond; halogen-carbon hydrogen bond; halogen (fluorine)
Trp82	N28, electrostatic-π-cation

Phe329	**13**, hydrophobic-π-π T-shaped
Leu286	C1, hydrophobic-alkyl
Trp82	**13**, hydrophobic-π-alkyl
Trp82	**13**, hydrophobic-π-alkyl
Trp231	C1, hydrophobic-π-alkyl
Trp231	C1, hydrophobic-π-alkyl
His438	**13**, hydrophobic-π-alkyl

Interactions between compound **14** and BChE.

Amino acid	Non-bonding interactions
Asp70	N25, electrostatic-attractive charge
HOH825	H55, hydrogen bond-water hydrogen bond; conventional hydrogen bond
Trp82	H40, hydrophobic-π-sigma
Phe329	**14**, hydrophobic-π-π T-shaped
Leu286	Cl21, hydrophobic-alkyl
Val288	C21, hydrophobic-alkyl
Trp82	**14**, hydrophobic-π-alkyl
Trp82	**14**, hydrophobic-π-alkyl
Trp82	**14**, hydrophobic-π-alkyl
Trp231	Cl21, hydrophobic-π-alkyl
Trp231	Cl21, hydrophobic-π-alkyl
His438	**14**, hydrophobic-π-alkyl

Interactions between compound **15** and BChE.

Amino acid	Non-bonding interactions
Asp70	N24, electrostatic-attractive charge
HOH825	H56, hydrogen bond-water hydrogen bond; conventional hydrogen bond
HOH825	H57, hydrogen bond-water hydrogen bond; carbon hydrogen bond
HOH831	H58, hydrogen bond-water hydrogen bond; carbon hydrogen bond
Ser287	H48, hydrogen bond-conventional hydrogen bond
Ser287	H49, hydrogen bond-carbon hydrogen bond
Asp70	H63, hydrogen bond-carbon hydrogen bond
Trp231	**15**, hydrophobic-π-π T-shaped
Trp231	**15**, hydrophobic-π-π T-shaped
Trp82	C7, hydrophobic-π-alkyl
Trp82	**15**, hydrophobic-π-alkyl
Trp82	C7, hydrophobic-π-alkyl
Trp82	**15**, hydrophobic-π-alkyl
His438	**15**, hydrophobic-π-alkyl
Leu286	**15**, hydrophobic-π-alkyl
Leu286	**15**, hydrophobic-π-alkyl

Interactions between compound **16** and BChE.

Amino acid	Non-bonding interactions
Asp70	N17, electrostatic-attractive charge
HOH910	H49, hydrogen bond-water hydrogen bond; conventional hydrogen bond
Pro285	H52, hydrogen bond-conventional hydrogen bond
Trp82	H39, hydrophobic-π-sigma
Tyr332	**16**, hydrophobic-π-π T-shaped
Trp82	**16**, hydrophobic-π-alkyl
Trp82	**16**, hydrophobic-π-alkyl

Trp82	C7, hydrophobic-π-alkyl
Trp82	**16**, hydrophobic-π-alkyl
His438	**16**, hydrophobic-π-alkyl

Interactions between compound **17** and BChE.

Amino acid	Non-bonding interactions
HOH831	H59, hydrogen bond-water hydrogen bond; carbon hydrogen bond
Ser287	H49, hydrogen bond-conventional hydrogen bond
Gln119	H52, hydrogen bond-conventional hydrogen bond
Ser287	H50, hydrogen bond-carbon hydrogen bond
Trp82	H42, hydrophobic-π-sigma
Phe329	**17**, hydrophobic-π-π T-shaped
Trp82	**17**, hydrophobic-π-alkyl
Trp82	C7, hydrophobic-π-alkyl
Trp231	Cl22, hydrophobic-π-alkyl
Trp231	Cl22, hydrophobic-π-alkyl
His438	**17**, hydrophobic-π-alkyl
His438	**17**, hydrophobic-π-alkyl

Interactions between compound **18** and BChE.

Amino acid	Non-bonding interactions
HOH989	H49, hydrogen bond-water hydrogen bond; conventional hydrogen bond
HOH1024	H49, hydrogen bond-water hydrogen bond; conventional hydrogen bond
Ser287	H52, hydrogen bond-conventional hydrogen bond
Ser198	H59, hydrogen bond-carbon hydrogen bond
Trp82	H40, hydrophobic-π-sigma
Trp231	**18**, hydrophobic-π-π T-shaped
Phe329	**18**, hydrophobic-π-π T-shaped
Trp82	**18**, hydrophobic-π-alkyl
Trp82	C7, hydrophobic-π-alkyl
Trp82	**18**, hydrophobic-π-alkyl
Trp82	**18**, hydrophobic-π-alkyl
Leu286	**18**, hydrophobic-π-alkyl
His438	**18**, hydrophobic-π-alkyl

Interactions between compound **19** and BChE.

Amino acid	Non-bonding interactions
Ser287	H61, hydrogen bond-conventional hydrogen bond
Pro285	H65, hydrogen bond-carbon hydrogen bond
Thr284	H66, hydrogen bond-carbon hydrogen bond
Ser287	H66, hydrogen bond-carbon hydrogen bond
Ser287	H66, hydrogen bond-carbon hydrogen bond
Trp82	**19**, hydrophobic-π-π T-shaped
Trp82	**19**, hydrophobic-π-π T-shaped
Phe329	**19**, hydrophobic-π-π T-shaped
His438	**19**, hydrophobic-π-π T-shaped
His438	**19**, hydrophobic-π-π T-shaped
Trp82	Cl22, hydrophobic-π-alkyl
Trp82	Cl22, hydrophobic-π-alkyl
Leu286	**19**, hydrophobic-π-alkyl

Interactions between compound **20** and BChE.

Amino acid	Non-bonding interactions
Ser287	H29, hydrogen bond-conventional hydrogen bond
Asp70	H40, hydrogen bond-carbon hydrogen bond
Val288	Cl28, hydrophobic-alkyl
Trp82	**20**, hydrophobic-π-alkyl
Trp82	C26, hydrophobic-π-alkyl
Trp82	**20**, hydrophobic-π-alkyl
Trp82	C26, hydrophobic-π-alkyl
Trp231	Cl28, hydrophobic-π-alkyl
Trp231	Cl28, hydrophobic-π-alkyl
His438	**20**, hydrophobic-π-alkyl

Interactions between compound **21** and BChE.

Amino acid	Non-bonding interactions
Asp70	N17, electrostatic-attractive charge
Asp70	H49, hydrogen bond-carbon hydrogen bond
Gln119	H51, hydrogen bond-carbon hydrogen bond
Gly116	H52, hydrogen bond-carbon hydrogen bond
Pro285	H58, hydrogen bond-carbon hydrogen bond
HOH930	**21**, other-π-lone pair
Trp82	**21**, hydrophobic-π-alkyl
Trp82	C7, hydrophobic-π-alkyl
Trp82	**21**, hydrophobic-π-alkyl
Trp82	**21**, hydrophobic-π-alkyl
Trp82	C7, hydrophobic-π-alkyl
Trp82	**21**, hydrophobic-π-alkyl
His438	**21**, hydrophobic-π-alkyl

Interactions between compound **22** and AChE.

Amino acid	Non-bonding interactions
Thr284	H80, hydrogen bond-conventional hydrogen bond
Ser287	H65, hydrogen bond-carbon hydrogen bond
Ser287	H67, hydrogen bond-carbon hydrogen bond
Pro285	H69, hydrogen bond-carbon hydrogen bond
Pro285	H72, hydrogen bond-carbon hydrogen bond
Pro285	H73, hydrogen bond-carbon hydrogen bond
Ser287	H74, hydrogen bond-carbon hydrogen bond
Trp82	N17, electrostatic-π-cation
Trp82	H53, hydrophobic-π-sigma
Trp82	**22**, hydrophobic-π-π T-shaped
Trp82	**22**, hydrophobic-π-π T-shaped
Trp82	**22**, hydrophobic-π-π T-shaped
Pro285	C7, hydrophobic-alkyl
Ile442	Cl22, hydrophobic-alkyl
Trp82	Cl22, hydrophobic-π-alkyl
Trp82	Cl22, hydrophobic-π-alkyl

Interactions between compound **23** and BChE.

Amino acid	Non-bonding interactions
Asp70	N35, electrostatic-attractive charge

HOH930	H78, hydrogen bond-water hydrogen bond; carbon hydrogen bond
HOH930	H79, hydrogen bond-water hydrogen bond; carbon hydrogen bond
HOH989	H80, hydrogen bond-water hydrogen bond; conventional hydrogen bond
HOH806	H81, hydrogen bond-water hydrogen bond; conventional hydrogen bond
Asp70	H81, hydrogen bond-conventional hydrogen bond
Ser198	H60, hydrogen bond-carbon hydrogen bond
Asp70	H78, hydrogen bond-carbon hydrogen bond
Trp231	N17, electrostatic-π-cation
Phe329	**23**, hydrophobic-π-π T-shaped
Phe329	**23**, hydrophobic-π-π T-shaped
Gly116;Gly117	**23**, hydrophobic-amide-π stacked
Gly116;Gly117	**23**, hydrophobic-amide-π stacked
Ala277	**23**, hydrophobic-alkyl
Trp82	**23**, hydrophobic-π-alkyl

Prediction of drug-likeness and blood-brain barrier (BBB) penetration

Table S1. *In silico* determined physicochemical characteristics of the tested aminoquinolines important for compounds to be orally active in humans and their *in silico* determined ability to pass the brain-blood barrier by passive transport.

Compound	M_W	logP	HBD	HBA	RB	PSA	Lipinski's rule	logBB	ADMET_BBB_level
1	369.94	4.27	2	3	6	36.95	√	0.496	1
2	383.96	4.33	2	3	7	36.95	√	0.423	1
3	397.99	4.85	2	3	8	36.95	√	0.603	1
4	426.05	5.74	2	3	10	36.95	– / √	0.885	0
5	454.10	6.63	2	3	12	36.95	–	1.167	0
6	403.49	4.54	2	3	7	36.95	√	0.490	1
7	417.52	4.60	2	3	8	36.95	√	0.509	1
8	383.96	4.55	2	3	7	36.95	√	0.609	1
9	546.24	7.64	1	3	10	28.16	–	1.487	0
10	574.2	8.23	1	3	12	28.16	–	-	4
11	588.32	8.68	1	3	13	28.16	–	-	4
12	397.99	4.75	2	3	7	36.95	√	0.632	1
13	431.55	5.02	2	3	8	36.95	√	0.626	1
14	410.02	5.03	2	3	8	36.95	√	0.731	0
14	363.55	4.14	2	3	7	36.95	√	0.434	1
16	391.60	4.95	2	3	9	36.95		0.791	0
17	417.98	6.07	2	3	5	36.95	– / √	1.184	0
18	417.98	6.07	2	3	5	36.95	– / √	1.184	0
19	482.07	7.35	2	3	6	36.95	– / √	-	4
20	*395.98*	*5.12*	0	3	3	19.37	√	1.100	0
21	410.00	5.41	0	3	4	19.37	√	1.199	0
22	510.17	4.53	2	5	11	43.43	–	0.366	1
23	524.19	4.54	2	5	12	43.43	–	0.466	1
Tacrine	234.73	2.63	1	2	0	38.91	√	0.018	1

In vitro determined antioxidative potential of 4-aminoquinolines

Table S2. The FRAP values of 10 µM and 100 µM 4-aminoquinolines. Determined FRAP values are shown as mean values ± standard error of three independent experiments.

Compound	FRAP (µM)	
	10 µM	100 µM
1	2.4 ± 1.3	20 ± 4
2	19 ± 2	64 ± 5
3	0	38 ± 6
4	1.9 ± 0.5	58 ± 10
5	9.5 ± 1.9	82 ± 4
6	4.6 ± 0.8	25 ± 6
7	7.1 ± 2.1	21 ± 7
8	33 ± 12	62 ± 7
9	3.4 ± 0.0	29 ± 6
10	13 ± 4	90 ± 6
11	5.3 ± 1.4	23 ± 4
12	24.2 ±0.0	37 ± 7
13	19 ± 7	64 ± 5
14	7.6 ± 2.3	86 ± 8
15	9.3 ± 2.2	224 ± 21
16	4.1 ± 0.4	46 ± 4
17	53 ± 4	512 ± 117
18	22 ± 6	300 ± 32
19	336 ± 12	2450 ± 210
20	2.9 ± 1.1	6.3 ± 1.6
21	6.0 ± 2.0	15 ± 5
22	11 ± 3	34 ± 8
23	1.4 ± 0.1	20 ± 5
tacrine	3.4 ± 0.0	15 ± 2
BHT	4800 ± 33	3021 ± 117
Trolox	523 ± 8	5812 ± 68

Chromatographic determination of lipophilicity

Lipophilicity of synthesized compounds was evaluated under reversed-phase thin-layer chromatographic condition using the vertical developing chamber (CAMAG, Muttenz, Switzerland) on 10 × 10 cm aluminium plates covered with octadecyl modified silica RP-18W F254s (Art. 5559, Merck, Darmstadt, Germany). The mobile phase contained organic modifier (methanol, acetone or dioxane), water and hydrochloric acid. The content of hydrochloric acid was kept constant at 5 %v/v while the portion of the organic modifier and water were changed. Methanol was varied in the range from 50-90 %v/v (for compounds **9**, **8**, **11** and **10** in range 70-90 %v/v) with an increment of 5%, acetone was varied in range 40-70 %v/v (compounds **9**, **8**, **11** and **10** in range 60-80 %v/v) with an increment of 5%, and dioxane in range 40-90 %v/v, (compounds **9**, **8**, **11** and **10** in range 50-90%), with an increment of 10%. The chamber was saturated with a mobile phase being used 15 minutes before chromatogram development. The solvent developing distance was 4.5 cm. Detection of individual zones was performed under UV light (254 and 360 nm). All experiments were performed at ambient temperature (22±2 °C). Retention parameters were calculated as previously described [7]. The log$D$ values of the investigated substances were determined by the interpolation procedure. Standard substances with known log$P$ and pK_a values were chromatographed simultaneously with investigated compounds on RP-18W F254s stationary phase with mobile phase methanol/water/hydrochloric acid 70/25/5 %v/v, pH = 0.5.

Equations (S1) and (S2) for monoacidic and diacidic base, respectively, were used to obtain log$D$ values of the standard compounds at pH = 0.5:

$$\log D = \log P - \log(1+10^{(\mathrm{pK}a\ -\ \mathrm{p}H)}) \qquad \text{(S1)}$$

$$\log D = \log P - \log(1+10^{(\mathrm{p}Ka1\ -\ \mathrm{p}H)} + 10^{(\mathrm{pK}a1\ +\ \mathrm{pka2}\ -\ 2\mathrm{p}H)}) \qquad \text{(S2)}$$

where log$P$ values correspond to the neutral form of the weak base. For diacidic bases pK_{a1} > pK_{a2}. Log$D$ values of the investigated substances were determined from the equation of the calibration curve obtained by plotting their R_M values against log$D$ values of standard substances [79].

Multivariate statistical analysis and modelling

Multivariate statistical analysis and modelling were performed by a PLS Toolbox software package (v. 5.7 Eigenvectors Inc. Manson, WA USA) for MATLAB (v. 7.8.0 R2009) (MathWorks, Natick, MA, USA).

To obtain a data overview, principal component analysis (PCA) was performed on both chromatographic data and calculated structural descriptors by using a singular value decomposition

algorithm (SVD) and a 0.95 confidence level for Q and T^2 Hotelling limits for outliers. Before the PCA, autoscaling was performed [7].

Partial least square regression was performed by the SIMPLS algorithm without forcing orthogonality constrains to the model which is supposed to condense Y-block variance into the first few latent variables. The quality of the regression fits is given by R^2_{cal}, R^2_{CV}, R^2_{pred}, *RMSEC, RMSECV,* and *RMSEP* [7]. The entire set of 191 molecular descriptors was used in modelling dependent variables, lipophilicity and biological activity. The number of variables in the final models was reduced after double PLS regression by removing variables with VIP scores lower than 1 which were considered as insignificant. The final PLS models were done by leaving one out cross-validation procedure. The prediction power of the obtained QSRR and QSAR models were tested on a set of randomly selected compounds (**2**, **5**, **9**, **13**, **17** and **21**).

Principal Component Analysis

The PCA performed on chromatographic data resulted in a two-component model that explains 97.25% of the total variance. The first principal component (PC1) accounted for 94.73% of data variance, and the second 2.52%. All compounds are inside of the Hotelling T^2 ellipse (**Figure S1A**). Generally, the order of the compounds along the PC1 axis follows the trend of increasing their lipophilicity values. In the upper left part of the score graph compounds **1-5**, **12**, **14**, and **15** which all have chlorine on C(7), were grouped. Compounds **1** – **5** form one small series of compounds with an increasing number of methylene groups in the spacer and methyleneadamantane moiety on terminal nitrogen. Compounds **12** and **14** have a branched spacer and compound **23** has the longest spacer between the C(4)-amino group on aminoquinoline and piperazine and the spacer between piperazine and adamantane. More lipophilic compounds **6** and **7** with trifluoromethyl substituent on C(7) position instead of chlorine, **6** and **7,** with unbranched spacer, so as compound **13** with branched spacer is positioned in the lower left part of the score graph. Close to them are compounds **8** (with ethyleneadamantane group on terminal nitrogen), **15**, **16** (with branched spacer and without substituents on C(7) position), compounds **17** and **18** (with benzene ring in the spacer), so as to compound **21** (with piperazine in the spacer and with the short chain). Compounds **20** and **22** with piperazine and longer spacer are isolated in the upper left part of the score graph, while compound **19** with naphthalene ring is singled out in the lower right part of the score graph. The addition of second ethyleneadamantane moiety drastically reduced the lipophilicity of the compounds **9**, **10**, and **11** which are located on the extreme right part of the score graph with positive values of PC1.

The graph of loadings (**Figure S1B**) indicate that chromatographic systems are ordered along PC2 axis. Mobile phases containing methanol have positive values of PC2, while mobile phases containing acetone and dioxane have negative values of PC2. PCA performed on reduced number of

calculated structural descriptors resulted in a four-component model that explains 95.25% of the total variance. The PC1 accounted for 42.69%, the PC2 for 24.54%, the PC3 for 8.27%, and the PC4 for 7.04% of data variance.

The score plot of the first two principal components obtained after PCA performed on the calculated structural descriptors (**Figure S1A**) revealed a similar arrangement of compounds as the graph of scores on retention data. Compound **22** lay outside the Hotelling T^2 ellipse, next to the border, while the compound **23** is on the very boundary of the ellipse. These compounds have the highest positive values of PC2. Opposite to them are compounds **17**, **18** and **19** with aromatic structures in the spacer with the highest negative values of PC2. The group with the highest positive PC1 values consists of compounds **9**, **10**, and **11** with two ethylenedamantane groups in their structure. Other compounds form a group which have a negative value of PC1 (except compound **5**) and PC2 values around zero. The graph of loadings (**Figure S1B**) indicates that the grouping of compounds around zero PC2 is influenced mainly by the descriptors which encode dipole and polar characteristics of compounds. Physicochemical descriptors which encode nonpolar characteristics of investigated compounds, together with experimentally determined log*D*, and topological descriptors are in the upper right part of the graph, influencing the position of most lipophilic compounds **9**, **10**, and **11**. Quantum-chemical descriptors HOMO, LUMO, PISA, and physico-chemical descriptors which encode permeability processes (QPPMDCK, QPPCaco, QPlogKp) are discriminative for grouping compounds with aromatic structures in the spacer (**17**, **18**, **19**). On the other hand, physicochemical descriptors which encode polar characteristics of compounds (accptHB, SP), so as quantum-chemical descriptor IP(eV) are discriminative for the position of compounds **22** and **23** with the piperazine in the spacer.

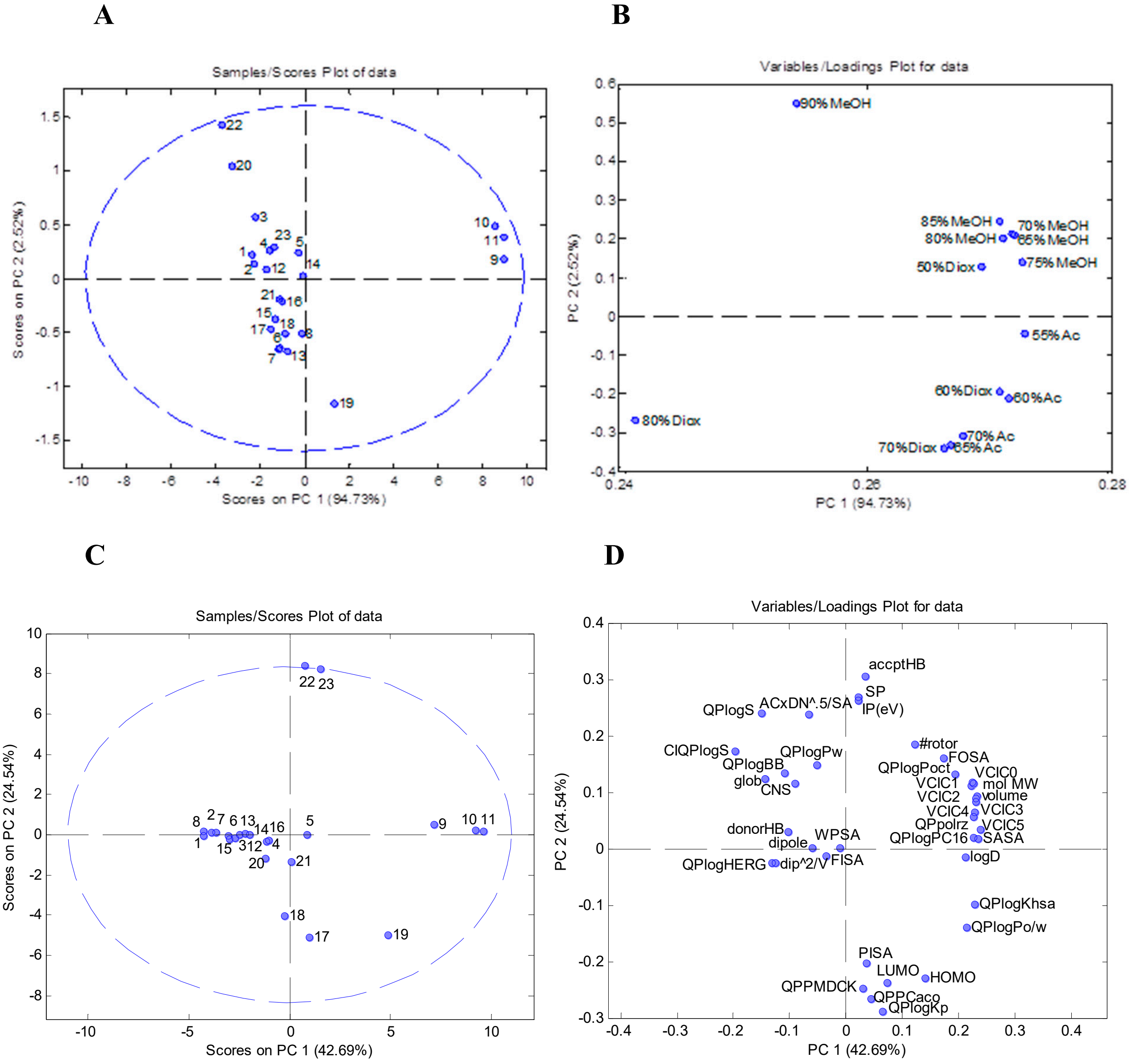

Figure S1. PC1–PC2 score plots of retention parameters and descriptors (A and C respectively) and factor loadings (B and D respectively).

Quantitative Structure-Property Relationship (QSPR)

Lipophilicity is one of the most important parameters in Quantitative Structure-Property /Activity Relationships (QSPR/ QSAR) which influences passive diffusion of drugs through biological membranes allowing the drug to reach the site of action. It affects pharmacodynamics, pharmacokinetics, and toxicological aspects of a drug candidate.

The thin-layer chromatography (TLC) method as a quick, simple and efficient method, has become one of the most commonly used procedures in the determination of lipophilicity. Relative lipophilicity parameters, determined by TLC method (R_M^0), are widely applied in QSPR analysis which correlates retention data R_M or R_M^0 and different structural descriptors of compounds [8].

The series of investigated compounds were chromatographed on RP-18 silica with mobile phase containing different portions of organic modifier (methanol, acetone or dioxane), water and hydrochloric acid. The highest selectivity provided methanol and acetone.

The dependence of the R_F values on the composition of the mobile phase are given in Tables S1-S3 (Supplementary material 3, SI3). The statistical details of retention and corresponding R_M^0 are summarized in Tables S4-S6 (SI3).

Mobile phase MeOH/H_2O/HCl, 70:25:5 (vol %), was used in the determination of the partition coefficients of the investigated compounds, $logD_{exp}$ at p*H* 0.5 (SI3 Table S7).

Increasing the number of methylene groups in the spacer led to the increase of R_M^0 and $logD_{exp}$ values in a series of compounds **1** - **5**. Introduction of the voluminous and strong electron-withdrawing CF_3-C(7) group on quinoline ring led to the increasing lipophilicity of compounds **6** and **7** in comparison to compounds **1** and **2**. Compounds **9**, **10**, and **11** are the most lipophilic due to the presence of two lipophilic ethyleneadamantane moiety, and their R_M^0 and $logD_{exp}$ values increased in the order **9** < **10** < **11**. Derivatives with branched spacer, series **12** – **16** exhibited higher values of lipophilicity compared to the unbranched analogues. Also, within the series, H-C(7) analogues, **15**, and **16** have lower lipophilicity in comparison to their Cl-C(7) analogues **12** and **14**, respectively. The introduction of aromatic rings in the spacer between aminoquinoline and adamantane part resulted in increasing lipophilicity, while the introduction of piperazine moiety in compounds **20** – **23** have the opposite effect.

In QSPR study PLS regression was used to obtain information regarding the nature of the intermolecular forces involved in passing the investigated molecules through the cell membrane. Experimentally determined values of lipophilicity R_M^0 and $logD_{exp}$ were correlated with calculated structural descriptors [9] obtained for studied compounds in their neutral form (SI3). The statistical performances of derived PLS models and the most contributing descriptors (VIP>1) are given in **Table S3** in decreasing order with the sign of their regression coefficients. All obtained models are with good predictive abilities and include similar descriptors which belong to several groups. Ring descriptors are used in the description of ring systems, topological descriptors encode different types of branching and the descriptors from the group of physico-chemical encode permeation abilities and solubility of compounds. The best obtained PLS model correlates $logD_{exp.}$ values with ring fusion degree (RF), ring bridge count (RBC), topological charge indexes of order 9 and 3 (TCIO9, TCIO3), valence connectivity index chi-3 (VCIC3), in a positive manner, and predicted water/gas partition coefficient (QPlogPw), normalized number of ring systems (Nnrs), and eccentric (ECC) in a negative manner.

Table S3. The statistical performances of QSPR models connecting the most contributing molecular descriptors with molecular lipophilicity[a]

Dependent variable / organic modifier	Statistical performance of the model	Structural descriptors included in PLS model[b]
R_M^0(MeOH)	RMSEC = 0.307, RMSECV = 0.388, RMSEP = 0.350<br>R^2cal = 0.921, R^2_{CV} = 0.874, R^2_{pred} = 0.927<br>PLS1: = 52.08% and 88.50%<br>PLS2: = 33.82% and 3.58%	TCIO9 (+), QPlogPo/w (+), QplogPw (+), #stars (+), CIQPlogS (-), TCIO3 (+), VCIC5 (+), VCIC4 (+), Q (+), CIX4 (+), SCIX4 (+), CIX5 (+), RDSRI (+), VCIC3 (+), C (+), VCIC2 (+), 2Z (+), H (+), QPlogHERG (+)
R_M^0(Acetone)	RMSEC = 0. 209, RMSECV = 0.306, RMSEP = 0.265<br>R^2cal = 0.890, R^2_{CV} = 0.768, R^2_{pred} = 0.856<br>PLS1: = 46.24% and 84.78%<br>PLS2: = 26.54% and 4.20%	RBC (+), VCIC5 (+), VCIC4 (+), QPlogKhsa (+), VCIC3 (+), VCIC2 (+), C (+), QPlogPo/w (+), IP(eV) (+), VCIC1 (+), CIQPlogS (-)
R_M^0(Dioxane)	RMSEC = 0.137, RMSECV = 0.173, RMSEP = 0.175<br>R^2cal = 0.899, R^2_{CV} = 0.841, R^2_{pred} = 0.968<br>PLS1: = 75.17% and 69.45%<br>PLS2: = 12.89% and 20.48%	RF (+), Nnrs (-), PE -S-OPLS (-), QPlogBB (-),CIQPlogS (-), LUMO (+), HOMO (+), #stars (+),QPlogPo/w (+), RBC (+),TCIO9 (+),TF-S-OPLS (-)
$logD_{exp.}$	RMSEC = 0.968, RMSECV = 1.231, RMSEP = 0.927<br>R^2cal = 0.968, R^2_{CV} = 0.942, R^2_{pred} = 0.981<br>PLS1: = 82.02% and 82.35%<br>PLS2: = 10.46% and 14.44%	RF (+), QPlogPw (-), RBC (+), Nnrs (-), TCIO9 (+), VCIC5 (+), QPlogKhsa (+), ECC (-),VCIC4 (+), TCIO3 (+),QPlogPo/w (+), QPlogKhsa (+)

[a]The details of the obtained PLS models and corresponding graphics that illustrate the contribution of a structural descriptor to molecular lipophilicity are provided in Graphics S1-S4.
[b]For abbreviation definitions and a complete list of molecular descriptors see SI3.

References

1. Koska, J., Spassov, V.Z., Maynard, A.J., Yan, L., Austin, N., Flook, P.K., Venkatachalam, C.M. Fully automated molecular mechanics based induced fit protein−ligand docking method. J *Chem Inf Model* **2008**, *48(10)*, 1965–1973.
2. Cheung, J., Rudolph, M.J., Burshteyn, F., Cassidy, M.S., Gary, E.N., Love, J., Franklin, M.C., Height, J.J. Structures of human acetylcholinesterase in complex with pharmacologically important ligands, *J Med Chem* **2012**, *55*, 10282−10286.
3. Nicolet, Y., Lockridge, O., Masson, P., Fontecilla-Camps, J.C., Nachon, F. Crystal structure of human butyrylcholinesterase and of its complexes with substrate and products, *J Biol Chem* **2003**, 278, 41141-41147.
4. Xu, Y., Colletier, J-P., Weik, M., Jiang, H., Moult, J., Silman, I., Sussman, J.L. Flexibility of aromatic residues in the active-site gorge of acetylcholinesterase: x-ray versus molecular dynamics. *Biophys J* **2008**, *95,* 2500–2511.
5. Šinko, G. Assessment of scoring functions and in silico parameters for AChE-ligand interactions as a tool for predicting inhibition potency. *Chem Bio Interact* **2019**, *308*, 216–223.
6. Rosenberry, T., Brazzolotto, X., Macdonald, I., Wandhammer, M., Trovaslet-Leroy, M., Darvesh, S., Nachon, F. Comparison of the binding of reversible inhibitors to human butyrylcholinesterase and acetylcholinesterase: a crystallographic, kinetic and calorimetric study. *Molecules* **2017**, *22(12)*, 2098.
7. Aleksic, I., Jeremic, J., Milivojevic, D., Ilic-Tomic, T., Šegan, S., Zlatović, M., Opsenica, D. M., Senerovic, L. N-benzyl derivatives of long-chained 4-amino-7-chloro-quionolines as inhibitors of pyocyanin production in Pseudomonas aeruginosa. *ACS Chem Biol* **2019**, *14(12)*, 2800-2809.
8. Dąbrowska, M., Starek, M., Chłon-Rzepa, G., Zagorska, A., Komsta, Ł. Jankowska, A., Slusarczyk, M., Pawłowski, M. Estimation of the lipophilicity of purine-2,6-dione-based TRPA1 antagonists and PDE4/7 inhibitors with analgesic activity. *Bioorg Med Chem Lett* **2021**, **49**, 128318
9. Todeschini, R., Consonni, V. Handbook of molecular descriptors, methods and principles in medicinal chemistry, 11th ed.; Mannhold, R., Kubinyi, H. Timmerman, H. Wiley-vch, Weinheim, Germany, **2000**.
